# The Transcriptome of Human Endometrial Mesenchymal Stem Cells Under TGFβR Inhibition Reveals Improved Potential for Cell-Based Therapies

**DOI:** 10.3389/fcell.2018.00164

**Published:** 2018-12-04

**Authors:** Shanti Gurung, Sarah Williams, James A. Deane, Jerome A. Werkmeister, Caroline E. Gargett

**Affiliations:** ^1^The Ritchie Centre, Hudson Institute of Medical Research, Melbourne, VIC, Australia; ^2^Department of Obstetrics and Gynaecology, Faculty of Medicine, Nursing and Health Sciences, Monash University, Melbourne, VIC, Australia; ^3^Monash Bioinformatics Platform, Monash University, Melbourne, VIC, Australia

**Keywords:** human endometrial MSC, RNA-sequencing, TGF-βR, culture expansion, angiogenesis, anti-fibrosis

## Abstract

Mesenchymal stem/stromal cells (MSCs) are multipotent cells with favorable properties for cell therapies and regenerative medicine. Human endometrium harbors a small population of perivascular, clonogenic MSCs (eMSCs) identified by the SUSD2 marker. As for other MSCs, eMSCs require extensive *in vitro* expansion to generate clinically relevant numbers of cells, resulting in spontaneous differentiation, replicative senescence and cell death, decreasing therapeutic potency. We previously demonstrated that A83-01, a TGF-β receptor inhibitor, maintained eMSC clonogenicity, promoted proliferation, prevented apoptosis and maintained MSC function *in vitro*. Here we compare the transcriptome of passaged eMSCs from six women cultured with and without A83-01 for 7 days. We identified 1206 differentially expressed genes (DEG) using a false discovery rate cut-off at 0.01 and fold change >2. Significant enrichment of genes involved in anti-inflammatory responses, angiogenesis, cell migration and proliferation, and collagen fibril and extracellular matrix organization were revealed. TGF-β, Wnt and Akt signaling pathways were decreased. Anti-fibrotic and anti-apoptotic genes were induced, and fibroblast proliferation and myofibroblast related genes were downregulated. We found increased MSC potency genes (*TWIST1*, *TWIST2*, *JAG1, LIFR*, and *SLIT2*) validating the enhanced potency of A83-01-treated eMSCs, and importantly no pluripotency gene expression. We also identified eMSCs’ potential for secreting exosomes, possibly explaining their paracrine properties. Angiogenic and cytokine protein arrays confirmed the angiogenic, anti-fibrotic and immunomodulatory phenotype of A83-01-treated eMSCs, and increased angiogenic activity was functionally demonstrated *in vitro*. eMSCs culture expanded with A83-01 have enhanced clinically relevant properties, suggesting their potential for cell-therapies and regenerative medicine applications.

## Introduction

Mesenchymal stem/stromal cells (MSCs) have been isolated from most postnatal adult tissues (Crisan et al., 2008), including perivascular MSCs in human endometrium (Schwab and Gargett, 2007; Masuda et al., 2012; Lv et al., 2014). MSCs from various sources have undergone clinical trials for treating many diseases, exploiting their regenerative and immunomodulatory functions. Systemic delivery and/or repeated administration requires large cell doses and most clinical trials use culture-expanded MSCs (von Bahr et al., 2012). During the culture process, MSCs undergo replicative senescence and spontaneous differentiation reducing the population of potent cells and diminishing efficacy *in vivo* (Gurung et al., 2015b; Samsonraj et al., 2015; Barragan et al., 2016).

Human endometrium (uterine lining) regenerates 4–10 mm mucosal tissue each month without scar formation for 400–500 cycles during a woman’s reproductive life (Jabbour et al., 2006), likely mediated by endometrial MSCs (eMSCs) and epithelial progenitor cells. eMSCs are enriched using CD140b/CD146 or SUSD2 surface markers (Schwab and Gargett, 2007; Masuda et al., 2012). Like other MSCs, eMSCs require culture expansion to generate sufficient numbers for clinical use. Transforming growth factor-b (TGF-β) is a pleiotropic cytokine signaling through the SMAD canonical pathway and crosstalks with other intracellular signaling pathways; Wnt, MAPK, Notch and JNK, independently of SMADs. TGF-β influences many biological functions including differentiation, angiogenesis, extracellular matrix (ECM) generation, fibrosis, immune surveillance, apoptosis, tissue homeostasis and repair (Massague, 2012).

To address issues associated with culture-expanded MSCs, we showed that long-term cultured eMSCs treated with a small molecule, A83-01 (Activin-like Kinase 4/5/7 inhibitor), targeting the TGF-β receptor (TGF-βR), retain their MSC properties; clonogenicity, multipotency, surface marker phenotype and proliferation with minimal senescence and apoptosis, and enhanced survival *in vivo* (Gurung et al., 2015b, Gurung et al., 2018). Here, we characterized the transcriptome of passage 6 (P6) human SUSD2^+^ eMSCs cultured with and without A83-01 for 7 days. The differential gene expression profile confirmed that A83-01 targets the TGF-βR and several non-canonical pathways. A83-01-treated eMSCs have angiogenic, anti-fibrotic and immunomodulatory properties, and maintain MSC potency genes without pluripotency gene expression, desirable properties of MSCs for treating many clinical conditions.

## Materials and Methods

### Human Endometrial Tissue Samples, SUSD2^+^ eMSC Isolation and Culture

This study was carried out in accordance with the ethical guidelines according to the National Health and Medical Research Council (NHMRC) of Australia’s National Statement on Ethical Conduct in Human Research. Human ethics approval was obtained from the Monash Health and Monash University Human Research Ethics committees (09270B). All subjects gave written informed consent in accordance with the Declaration of Helsinki.

**FIGURE 1:**
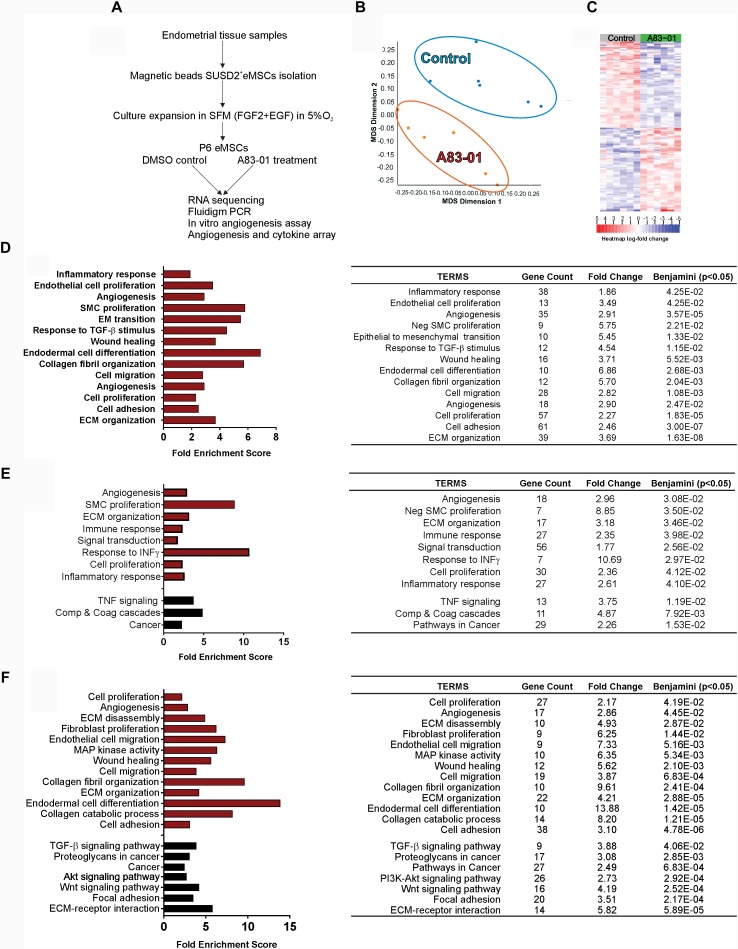
Transcriptome of A83-01 treated P6 eMSCs. **(A)** Schematic showing study design and experimental steps before RNA sequencing, and subsequent validation and functional assays. **(B)** Principle Component Analysis demonstrating clear separation of gene transcripts between A83-01-treated and control eMSCs. Each dot represents an experimental replicate. **(C)** Heat map showing the top 200 up- and down-regulated DEGs with FDR <0.01 and >2 fold change (*n* = 6). **(D)** GO analysis of biological processes of all DEGs using DAVID 6.8. A83-01 altered the expression of genes involved in the inflammatory response, angiogenesis, and ECM and collagen fibril organization. **(E,F)** GO and KEGG analysis of functional annotations and pathways of upregulated **(E)** and downregulated **(F)** genes. Red bars (GO) and black bars (KEGG). Fold enrichment change were analyzed by Benjamini–Hochberg (*p* < 0.05).

SUSD2^+^ eMSCs were obtained from eight healthy pre-menopausal women undergoing endometrial biopsy for non-endometrial pathologies who were not taking exogenous hormones for 3 months prior to surgery, using our published protocols for isolation and culture (Masuda et al., 2012; Gurung et al., 2015b). The cells were cultured in serum free medium [with bFGF and EGF (SFM)] and at passage 6 (P6) they were treated with 0.01% DMSO (vehicle control) or 1 μM A83-01 for 7 days with media changed every 48 h (Figure 1A). At the end of 7 days, cells were collected and half were pelleted for RNA extraction and the other half were lysed for protein array from both treated and control groups. Similarly, the condition media of the last 48 h were collected for functional assays and protein array studies.

### RNA Sequencing and Data Analysis

RNA was extracted using PureLink RNA mini Kit (Ambion, Invitrogen) including treatment with RNase-free DNase (Qiagen); RIN > 9.1 μg of ribosomal depleted RNA was processed using the Illumina TruSeq Poly-A mRNA Library Pro Kit protocol 15031047 RevD (Illumina, Hayward, CA, United States) to generate indexed cDNA libraries for each sample. Libraries were verified by Bioanalyzer. A single equimolar pool was prepared and sequenced with 50 base pair single-end reads (∼50–70 million reads/sample) with Illumina HiSeq3000. Image analysis, raw nucleotide base calling, conversion from bcl to fastq format, and quality filtering used Illumina CASAVA v1.8.2 software (Illumina, Hayward, CA, United States) with >97% of reads having quality >Q30.

RNA-sequenced reads were mapped against the Human Reference Genome GRCh38 and Ensembl 87 gene annotation using STAR (v2.5.2) (Dobin et al., 2013) with the RNAsik pipeline and manipulated with samtools (v1.3.1) (Li et al., 2009) and picard tools (v2.8.2) (Anders et al., 2015). From mapped reads, raw read counts/gene were quantified using htseq-count (v0.6.1p1), filtered to ≥ 10 reads in at least one sample for subsequent analysis and differential expression calculated across paired A83-01-treated and control samples using voom (Law et al., 2014) and limma (v3.26.9) (Ritchie et al., 2015) via the degust interface. Differentially expressed genes (DEGs) were defined as FDR significance threshold of *p* < 0.01 and absolute log change of 1 cutoff (>2-fold change). Functional annotation of the DEGs was performed by KEGG and gene ontology (GO) analysis using DAVID 6.8 (Huang da et al., 2009).

### Transcriptional Validation Using Fluidigm PCR

Ninety-five candidate DEGs were selected for validation based on GO and KEGG analysis and the literature. cDNA was prepared (50 ng/μl) and loaded to pre-primed 96.96 Dynamic Array Integrated Fluidic Circuit (Fluidigm), amplified and detected using TaqMan assays. Target gene expression was normalized to β-actin and relative gene expression and fold change was calculated using the 2^-ΔΔCT^ method.

### Angiogenesis Assays

#### Scratch Assay

P6 eMSCs treated with and without A83-01 were grown to confluency on fibronectin-coated 6-well plates in SFM in 5% O_2_, scratched with a 200 μl pipette tip and the same media was replaced. The covered area was analyzed at 0, 24, and 48 h using ImageJ software.

#### Matrigel Tube Forming Assay

μ-slides (Ibidi) were coated with 10 μl growth factor-reduced Matrigel at 37°C for 1 h. P6 control and A83-01-pre-treated eMSCs (16,000 cells/well) were seeded on the Matrigel in their respective media. Images were captured at 3, 16, and 24 h at X 4 magnification using Olympus BX41 microscope (Olympus).

### Human Cytokines and Angiogenesis-Related Antibody Array

The secretion of cytokines in the condition medium (CM) was measured using Human XL cytokine Array Kit (R&D Systems) while the angiogenic-related proteins were measured in the cell lysate using Human Angiogenesis Array Kit (R&D Systems) following the manufacturer’s instructions. Arrays were imaged by Quantity One software with the ChemiDocTM XRS+ system. Protein signals were quantified using ImageJ software.

### Statistical Analyses

Biological pathways from GO-TERM/Biological Processes and KEGG analysis were considered significant with Benjamini–Hochberg test *p* < 0.05 in DAVID 6.8. Differences between two groups were analyzed with Wilcoxon matched-pair signed rank test, groups over time were analyzed using 2-way ANOVA with Sidak’s multiple comparisons test and more than two groups were analyzed using one-way ANOVA with Turkey’s multiple comparisons test using GraphPad Prism 7 and considered statistically significant at *p* < 0.05.

## Results

### A83-01 Markedly Alters the Transcriptome of Cultured eMSCs

To determine the gene profile of A83-01-treated eMSCs, we undertook global transcriptome profiling of SUSD2^+^ eMSCs isolated from six donors expanded with or without A83-01 at P6 for 7 days using RNA sequencing (Figure 1A). We chose P6 to represent substantial culture of eMSCs, which comprise a mixture of differentiated SUSD2^-^ fibroblasts and diminished numbers of SUSD2^+^ eMSCs (Gurung et al., 2015b). Fastq, metadata spreadsheet and Table of Counts have been deposited in the National Centre for Biotechnology Information Gene Expression Omnibus/Sequence Read Archive with GEO accession no. GSE115137. Principal Component Analysis and heat map of the top 200 up- and downregulated genes clustered the samples into two distinct groups based on treatment with or without A83-01 (Figures 1B,C). A total of 21,830 transcripts were detected in the P6 eMSCs (NCBI account). There were 1206 DEGs (609 upregulated/Supplementary Table S1 and 597 downregulated/Supplementary Table S2) in eMSCs treated with A83-01 with >2-fold change and false discovery rate (FDR) <0.01, indicating marked changes in the transcriptome of A83-01-treated eMSCs.

To explore the biological relevance of the DEGs, we performed gene set enrichment analysis to functionally classify the genes on biological processes. GO analysis revealed enrichment of inflammatory response, endothelial cell proliferation, angiogenesis, wound healing, cell adhesion, collagen fibril and ECM organization, cell migration and proliferation pathways (Figure 1D and Supplementary Table S3). GO and KEGG functional enrichment analysis of upregulated DEGs revealed additional pathways enriched for ECM organization, response to IFN-γ, TNF signaling and complement and coagulation cascades (Figure 1E and Supplementary Table S4). Analysis of downregulated genes showed enrichment for fibroblast proliferation, collagen catabolic process, and TGF-β, Wnt and Akt signaling pathway genes in A83-01-treated eMSCs (Figure 1F and Supplementary Table S4), suggesting that A83-01 induced anti-inflammatory, growth, proliferation and angiogenesis genes and prevented spontaneous differentiation of eMSCs into ECM-secreting fibroblasts.

### A83-01 Enhances the Angiogenic Properties of eMSCs

#### A83-01 Promotes Angiogenic Signaling in eMSCs

**FIGURE 2:**
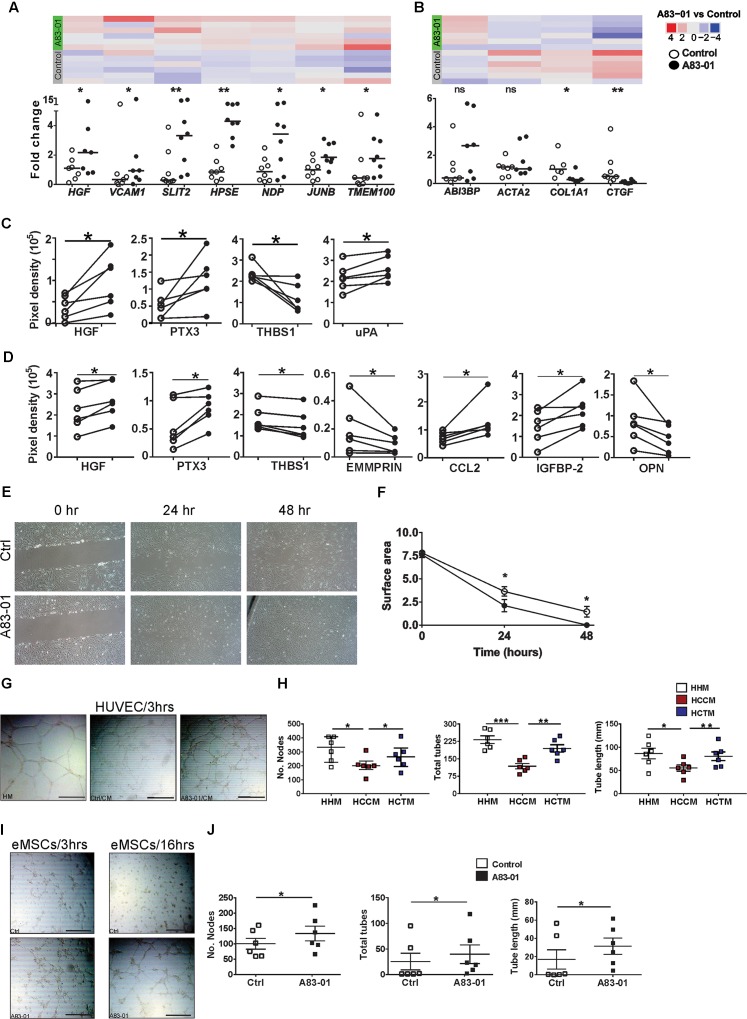
A83-01 treated-eMSCs secrete angiogenic factors and enhance angiogenesis. **(A,B)** Validation of angiogenesis-related genes in A83-01-treated versus control eMSCs using Fluidigm qPCR. Data points from each individual donor (*n* = 6–8) are presented; black circles, A83-01-treated; white circles, vehicle controls, bars are medians. The corresponding heat map of RNA-seq is from six independent donor eMSCs (original set). Angiogenesis-related proteins in **(C)** cell-lysates and **(D)** CM in A83-01-treated and control eMSCs. (*n* = 6, graphs are symbols and line to demonstrate corresponding pairs of A83-01 treated (black circles) and control (white circles) eMSCs. **(E)** Representative images of A83-01-treated and control eMSCs using *in vitro* scratch test at 0, 24, and 48 h assessing cell migration and proliferation and **(F)** graph of wound closure. Data are presented as means ± SEM, (*n* = 6). **(G)** Representative images showing enhanced tube formation by HUVECs incubated with HM, CM from A83-01-treated or control eMSCs (scale bar: 500 μm) 3 h after cell seeding. **(H)** Graphs show quantification of number of nodes, tubes and total tube length. Data are medians with interquartile range *n* = 6. **(I)** Representative Images of enhanced tube formation following culture of eMSCs with A83-01 and control 3 and 16 h after seeding in Matrigel (scale bar: 500 μm). **(J)** Graphs show quantification of number of nodes, tubes and total tube length. Data are median with interquartile range, *n* = 6. ^∗^*p* < 0.03, ^∗∗^*P* < 0.002, ^∗∗∗^*P* < 0.0002. HHM: HUVECs with HUVEC media (HM), HCCM: HUVECs with control eMSC conditioned media, HACM, HUVECs with A83-01 treated-eMSC CM.

**FIGURE 3:**
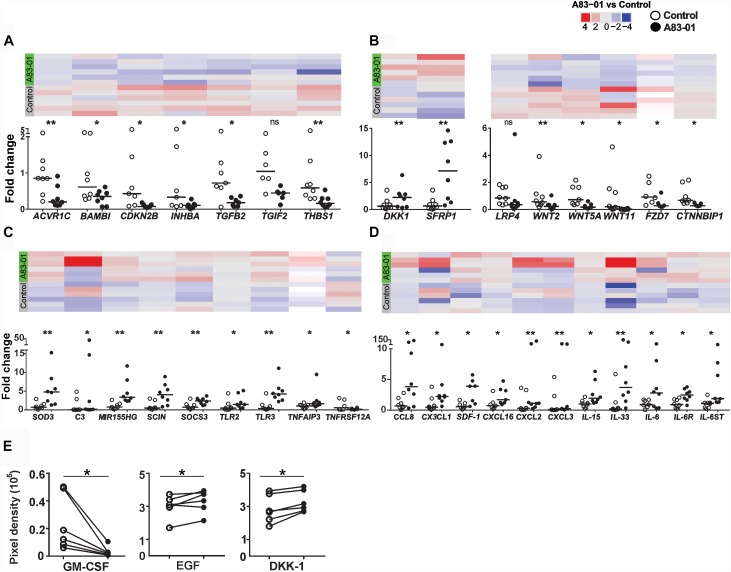
Validation of DEGs in A83-01-treated and untreated-eMSCs in patient samples. **(A)** TGF-β, **(B)** Wnt pathways, and **(C,D)** cytokines and their receptor-related gene expression in A83-01-treated and control P6 eMSCs were validated using Fluidigm qPCR. Black circles, A83-01-treated, white circles, vehicle controls. Individual data points are plotted, bars are medians. Panels above each graph show the heat map of the corresponding genes by RNA seq. **(E)** Cytokine proteins were determined in the CM of A83-01-treated and control eMSCs using a human XL cytokine array kit. (Graphs are symbols and line to demonstrate corresponding pairs) [^∗^*p* < 0.03, ^∗∗^*p* < 0.002, (*n* = 6–8)].

Angiogenesis is important in regenerative medicine. A83-01 altered angiogenesis genes in eMSCs, upregulating 18 pro-angiogenesis and downregulating 17 angiogenesis inhibitor genes (Figures 1D–F), conferring an angiogenic phenotype. *SFRP1*, an inhibitor/modulator of Wnt/Frizzled pathway, was highly expressed and the most significantly upregulated gene in A83-01-treated eMSCs (Supplementary Table S1). SFRP1 plays a vital role in vessel formation and maturation, enhancing MSC contribution to vascular cells during neovascularization and vessel maturation (Dufourcq et al., 2008). Many other pro-angiogenic genes were also upregulated including *HGF*, *VCAM1*, *PGF*, *HPSE* (Supplementary Table S4). In contrast, angiogenesis inhibitory genes were downregulated including *THBS1*, *CTGF*, *COL4A1*, and *COL4A2* (Supplementary Table S4). Using Fluidigm qPCR in the original and two extra samples (*n* = 8) for several pro-angiogenic and anti-angiogenic genes we confirmed upregulation of *HGF*, *VCAM1*, *SLIT2*, *HPSE*, *NDP*, *JUNB*, *TMEM100* (Figure 2A) and *SFRP1* (Figure 3B) and downregulation of *COL1A1* and *CTGF* (Figure 2B) suggesting that A83-01 promotes angiogenic signaling in human eMSCs.

#### A83-01-Treated eMSCs Secrete Increased Pro-angiogenic and Reduced Anti-angiogenic Factors

Having demonstrated that A83-01 modulated numerous angiogenesis genes we then sought to identify pro-angiogenic and anti-angiogenic proteins produced by A83-01-treated and control eMSCs. Using a human angiogenesis antibody array (55 proteins) we detected 15 proteins in eMSC lysates. Pro-angiogenic proteins HGF, PTX3 and uPA were increased in A83-01-treated eMSCs and the anti-angiogenic protein THBS1 was decreased (Figure 2C). Both HGF and PTX3 were also secreted in greater amount into CM of A83-01-treated compared to control eMSCs, while THBS1 secretion decreased (Figure 2D). This suggests A83-01 promotes pro-angiogenic and inhibits anti-angiogenic protein production and secretion by eMSCs.

### A83-01 Treated-eMSCs Have Increased Migratory Capacity

A key property of angiogenic cells is to migrate through tissues to initiate new blood vessels. To determine whether the increased angiogenic gene and protein profile of A83-01-treated eMSCs influenced eMSC migratory function, we assessed wound closure *in vitro*. Confluent eMSC cultures showed greater wound closure in the presence of A83-01 compared to control at 24 and 48 h (Figures 2E,F) indicating that A83-01-treated eMSCs have increased migratory and proliferative capacity.

### Pro-angiogenic Factors Secreted by A83-01-Treated eMSCs Promote Endothelial Cell Tube Formation

To further investigate functional angiogenic effects of A83-01 on eMSCs, we assessed the effect of CM from A83-01-treated and control eMSCs on human umbilical vessel endothelial cells (HUVECs) in a 3D tube-forming angiogenesis assay. HUVECs cultured in endothelial growth medium served as positive control. HUVECs formed tubes within 3 h (Figure 2G). Tube formation was more extensive in CM from A83-01-treated eMSCs showing increased number of nodes, tubes and tube lengths (Figure 2H) than untreated control eMSCs, suggesting the identified secreted pro-angiogenic factors (Figure 2D) have functional effects. In addition, the tube formation ability of CM from A83-01-treated eMSCs was comparable to that of HUVEC media (Figure 2H). Next, to determine if eMSCs themselves formed vessels as previously reported (Masuda et al., 2012), control and A83-01-treated eMSCs were assessed in the 3D tube-forming assay (Figure 2I). A83-01-treated eMSCs formed vessels at 3 h, with increased numbers of nodes, total tubes and tube length compared to the control by 16 h (Figures 2I,J). This shows that A83-01-treated eMSCs not only secreted factors promoting angiogenesis but also self-contribute to angiogenesis. Overall, our findings from RNA-seq, Fluidigm qPCR, protein and *in vitro* angiogenesis assays reinforce that A83-01-treated eMSCs may have pro-angiogenic effects when applied *in vivo* for regenerative therapy.

### A83-01 Treated-eMSCs Have an Anti-fibrotic Gene Profile

Another target of MSC-therapy is fibrosis to promote scarless wound healing. Upon tissue injury, autocrine production of TGF-β directs MSC differentiation into myofibroblasts, cells primarily responsible for fibrosis. We mined the RNA-seq data for fibrosis-related genes. DEGs between A83-01 treated and control eMSCs involved pathways regulating ECM organization, collagen catabolism, and fibril organization processes (Figures 1D–F). Table 1 shows downregulation of pro-fibrotic and myofibroblast genes including *ELN*, *CTGF*, *COL1A1*, *FN1*, *MMP9*, and *MMP2*, and an upregulation of anti-fibrotic-related genes including *HGF*, *CCL2*, *FBLN1*, *VWA1*, *TGFB3*, and *MIR155HG* in A83-01-treated eMSCs. Transcripts for the proteasome (cathepsins, calpains, thrombospondin, metalloproteinases, and integrins) required for activating TGF-β by cleaving latency-related peptide were also reduced (Table 1). We validated several key fibrosis-related genes in A83-01-treated eMSCs confirming an increase in anti-fibrotic *HGF* and a reduction in pro-fibrotic *COL1A1* and *CTGF* by Fluidigm qPCR (Figures 2A,B). A83-01-treated-eMSC CM also contained increased anti-fibrotic proteins HGF, PTX3, CCL2, and IGFBP-2 (Figures 2C,D) and decreased pro-fibrotic proteins EMMPRIN and OPN (Figure 2D). The pro-angiogenic and anti-fibrotic gene and protein profile of A83-01-treated eMSCs suggests their potential in promoting scarless healing could be harnessed for regenerative medicine purposes.

**Table 1:** Differentially regulated fibrosis-related genes in A83-01-treated versus control eMSCs.

Pro-Fibrotic genes	Fold change log_2_		FDR (<0.01)	Ave Expr
	Control	A83-01		
*ELN*	0	-5.00	2.16E-05	2.35
*GREM1*	0	-2.13	3.73E-05	7.07
*CXCR4*	0	-4.99	4.62E-05	-0.79
*IGF2*	0	-2.18	7.29E-05	4.92
*TGFB2*	0	-2.30	8.00E-05	2.77
*EDN1*	0	-2.84	8.15E-05	0.05
*LRRC17*	0	-2.85	1.32E-04	6.47
*MGP*	0	-4.10	1.34E-04	3.42
*PDGFC*	0	-1.83	1.37E-04	6.29
*FN1*	0	-2.38	1.46E-04	12.03
*MMP2*	0	-1.75	3.61E-04	11.21
*COL1A1*	0	-1.39	4.83E-04	14.07
*MIR199A2*	0	-1.88	5.73E-04	-1.98
*TNC*	0	-2.44	7.23E-04	9.13
*THBS1*	0	-2.45	9.42E-04	10.16
*COMP*	0	-5.57	6.77E-04	-0.94
*LOX*	0	-1.89	1.04E-03	8.46
*PDGFB*	0	-1.66	1.56E-03	2.19
*NOX4*	0	-2.56	1.60E-03	1.55
*CDH2*	0	-2.40	2.34E-03	4.43
*CTGF*	0	-3.36	3.99E-03	7.56
*SPP1*	0	-2.50	1.48E-02	1.28
*ACTA2*	0	-1.41	2.62E-02	8.15
*SERPINE1*	0	-1.33	4.35E-02	7.55
*MMP9*	0	-1.27	7.55E-02	-2.35
*COL4A2*	0	-1.32	7.96E-04	11.53
*COL4A1*	0	-1.82	6.54E-04	10.89
*COL11A1*	0	-4.02	7.33E-05	1.91
*COL10A1*	0	-2.70	2.16E-05	2.35
*CXCL12*	0	1.81	1.73E-02	7.78
*HGF*	0	1.21	9.64E-03	7.86
*CCL2*	0	1.67	1.63E-02	6.49
*FGF7*	0	1.66	4.52E-04	5.74
*FBLN1*	0	1.56	7.06E-04	10.42
*VWA1*	0	1.60	2.42E-04	3.46
*CRISPLD2*	0	2.22	5.55E-04	5.07
*IL6*	0	1.81	1.20E-02	4.45
*TGFB3*	0	1.74	1.40E-02	3.42
*MIR155HG*	0	2.05	7.52E-05	2.19
**Genes for Latent TGF-β activating enzymes**
*CTSL*	0	1.12	4.40E-03	8.98
*CTSW*	0	1.70	7.26E-02	-3.86
*CAPN6*	0	-2.00	4.62E-05	1.51
*CAPNS2*	0	-3.98	6.04E-04	-4.41
*CAPN9*	0	-1.28	1.31E-01	-4.64
*THBS1*	0	-2.45	9.42E-04	10.16
*MMP3*	0	-1.73	1.99E-03	8.35
*MMP10*	0	-4.80	7.04E-05	5.73
*MMP2*	0	-1.75	3.61E-04	11.21
*MMP15*	0	-1.84	5.77E-04	1.52
*ITGB3*	0	-1.46	1.73E-03	4.06
*ITGB5*	0	-1.59	5.68E-06	8.80
*ITGA1*	0	-1.92	4.93E-04	7.09
*ITGA4*	0	-1.62	5.68E-04	6.32

### A83-01 Treatment of eMSCs Enhances Immunomodulatory Properties

The pleiotropism of TGF-β signaling in eMSCs was demonstrated by >1200 DEGs and pathway analysis in control versus A83-01-treated eMSCs, and by validating DEGs in TGF-β and Wnt signaling pathways (Figures 3A,B). As expected, TGF-β signaling pathway genes were down regulated in A83-01-treated eMSCs. To explore further, the inflammatory and immune response phenotype of A83-01-treated and control eMSCs was investigated by gene profiling. *SOD3* (superoxide dismutase protein 3), an antioxidant enzyme coding transcript was the most significantly upregulated gene in A83-01-treated eMSCs compared to controls (Supplementary Table S1) and validated with Fluidigm qPCR (Figure 3C). *SOD2* was similarly upregulated in A83-01-treated cells (Supplementary Table S1), while *SOD1* was highly expressed although not differentially regulated. Several cytokines or chemokines and their receptors were among the differentially upregulated genes in the A83-01-treated eMSCs compared to controls, including the chemokine transcripts *CXCL12*, *CXCL16*, and *CCL8* (Supplementary Table S1). Many inflammatory and immune response genes were upregulated in eMSCs after A83-01 treatment: interleukin and interleukin-related genes *IL-15*, *IL-33*, and *IL-6ST* and their corresponding receptors *IL-15RA*, *IL-1R1*, and *IL-6R* (Supplementary Tables S3, S4); *TNF* and the TNF-related genes *PTGS2*, *JUNB*, *SOCS3*, and *TNFAIP3*; and interferon-gamma related genes *IFI6*, *IFI44L*, *IFITM3*, and *OAS1* (Supplementary Table S3). Other highly expressed and significantly upregulated genes were *PLA2G4A*, *PTGS2*/*COX*-2, and *PTGES*, all required for prostaglandin (PG) E2 synthesis (Supplementary Tables S1, S3, S4). Likewise, genes for innate immune system proteins, Toll-like receptors (TLR), were expressed by eMSCs; *TLR 1*, *2*, *3*, *4*, *5*, and *6*, and *TLR2* and *TLR3* were significantly upregulated following A83-01 treatment (NCBI account, Figure 3D). Together, these data suggest A83-01-treated eMSCs have a major role in modulating inflammatory responses.

The complement cascade interacts with the immune system (Kolev et al., 2014). We found 11 upregulated transcripts for complement cascade genes, including *C2*, *CFB*, *CFD*, *CFI*, and *C3* (Supplementary Table S4). Transcripts of plasma fluid-based major complement-regulatory proteins I and clusterin were also upregulated. Highly expressed, although not differentially regulated, were fluid-based regulators *CFH*, *FHL1*, *SERPING1* (C1 inhibitor), *CLU* (Clusterin), S protein and membrane-based complement regulators, *CD46*, *CD55*, and *CD59* (NCBI account). Factors significantly downregulated were tissue factor, coagulation factor 13A1 and platelet agonist, *COL1A1* responsible for thrombus formation. In addition to decreased expression of prothrombotic factors, the haemostatic regulators, tissue factor pathway-inhibitor (TFPI) and tissue plasminogen activator (*tPA*) were upregulated. Collectively, this gene profile suggests that A83-01-treated eMSCs will contribute to complement-mediated tissue homeostasis and tissue repair. We also validated some of the above mentioned immune, inflammatory, chemotaxis and complement cascade-response genes using Fluidigm qPCR including *C3*, *SCOS3*, *TLR2/3*, *TNFAIP3, SDF-1, IL-15, IL-33*, and *CXCL16* (Figures 3C,D).

Next, we investigated the protein profiles using Human XL Cytokine Array (105 cytokines) in the CM collected from control and A83-01-treated eMSC cultures. CCL2, GM-CSF, IGFBP-2 (IGF binding protein 2), DKK-1 and EGF increased following A83-01 treatment (Figure 3E), supporting the pleiotropic effects of blocking TGF-β signaling in eMSCs by A83-01 and confirming these eMSCs have a broad immunomodulatory phenotype.

### Differential Expression of Exosome Biogenesis and Cargo Transcripts in A83-01-Treated eMSCs

The MSC therapies exert their therapeutic effect by a paracrine mechanism, secreting biological factors (Tolar et al., 2010; Le Blanc and Davies, 2015) as well as differentiating *in vivo* (Kunter et al., 2006; Aslam et al., 2009; Pierro et al., 2013; Park et al., 2018). MSC-derived exosomes as the paracrine effector of MSCs has recently gained momentum in regenerative medicine (Bian et al., 2014; Phinney and Pittenger, 2017). Membrane bound exosomes carrying protein, lipid and microRNA cargo released by MSCs promote wound healing (Bian et al., 2014; Teng et al., 2015; Phinney and Pittenger, 2017). We investigated the expression of exosome-related genes in culture-expanded eMSCs. Supplementary Table S5 shows >100 of the topmost exosomal-marker genes listed in the ExoCarta database were identified in our P6 eMSCs. Supplementary Table S6 shows 81 microRNAs (exosomal cargo) expressed in control and A83-01-treated eMSCs. Of these, 11 miRNAs were significantly enhanced and 4 decreased in A83-01-treated-eMSCs. To confirm the synthesis of exosomes in eMSCs as opposed to plasma membrane releasing extracellular vesicles, we looked for endosome-associated protein markers and identified expression of 11 Annexins, 19 Rab GTPases, 4 SNAREs and flotillin (Supplementary Table S5). We also identified high expression of multivesicular endosome biogenesis protein transcripts, including the endosomal-sorting complex required for transport machinery; *VPS35*, *VPS26A*, *VPS28*, *ALIX*/*PDCD6IP*, and *TSG101* (tetraspanin) (Supplementary Table S5). Similarly, transcripts of proteins required for exosome sorting (*COPA*, *CLTC*), trafficking (*KIF23*/*14*), release (*NSF*, *VAT1*), and recognizing and uptake (*CD44*, *ALCAM*) by target cells were highly expressed. Most importantly we found high expression for classic-exosome markers, CD63, *CD9*, *CD81*, and *CD82*. eMSCs in both groups expressed high levels of major histocompatibility complex I (A, B, C, E, F, and G) transcripts (NCBI account, Supplementary Table S5), exosomal cargo that protects against natural killer cell spontaneous cytolytic activity. Although not differentially expressed between control and A83-01-treated eMSCs, exosome marker expression levels were very high (Supplementary Table S5) indicating eMSCs may secrete exosomes and A83-01 treatment may not adversely affect their potential biogenesis, trafficking and release.

### MSC Potency and Proliferation Genes Are Enriched in A83-01-Treated eMSCs

**FIGURE 4:**
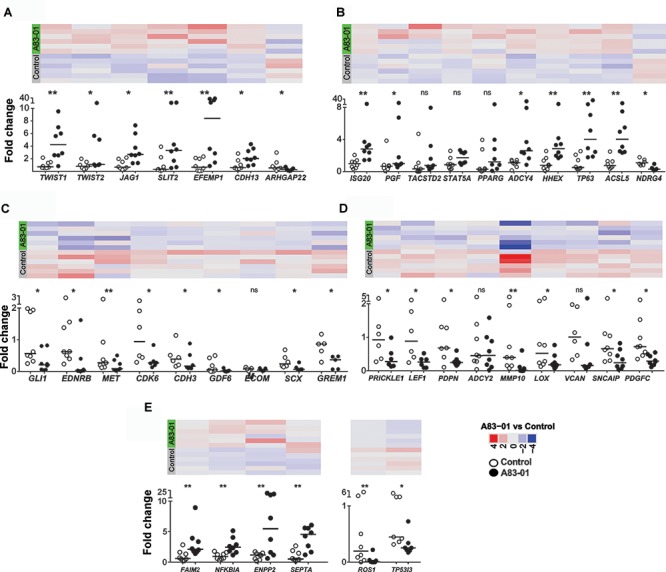
Differentially expressed genes (DEGs) confirmed by Fluidigm qPCR. **(A)** MSC potency, **(B)** proliferation, **(C)** anti-proliferation, **(D)** fibroblast and **(E)** apoptosis-related genes were validated in control and A83-01 treated P6 eMSCs. Data points (black circles, A83-01-treated, white circles, control) and medians are shown. Panels above each graph shows the heat map of the corresponding genes in RNA seq (^∗^*p* < 0.03, ^∗∗^*p* < 0.002, *n* = 6–8).

Previously we demonstrated A83-01-treated eMSCs had enhanced MSC properties (Gurung et al., 2015b). We mined the RNA-seq data for surface marker transcripts associated with high potency MSCs. A83-01-treated eMSCs have four–eightfold more transcripts than controls for 6 of 8 reported MSC potency-related genes; *TWIST1*, *TWIST2*, *JAG1*, *SLIT2*, *EFEMP1*, and *CDH13* confirming improved potency at the transcriptional level (Figure 4A and Table 2) as shown previously by increased colony forming efficiency (Gurung et al., 2015b).

**Table 2:** Gene profile of MSC potency in A83-01-treated versus control eMSCs.

MSC potency markers	Fold change log_2_ (A83-01)	FDR (<0.01)	Ave Expr
*TWIST1*	1.648	1.39E-03	5.15
*TWIST2*	1.298	1.01E-03	6.77
*JAG1*	1.758	3.81E-04	4.82
*SLIT2*	2.055	1.89E-05	4.33
*EFEMP1*	3.763	4.62E-05	1.06
*TNFRSF21*	1.235	7.36E-03	8.42
*CDH13*	1.373	1.28E-05	7.35
*ARHGAP20*	1.856	1.29E-04	2.66
**MSC positive markers**			
*THY1/CD90*	0.05	7.59E-01	9.38
*NT5E/CD73*	0.65	1.61E-02	9.01
*ENG/CD105*	0.22	1.37E-01	7.85
*ITGA6/CD49f*	0.67	5.66E-02	7.38
*ALCAM/CD166*	-0.60	1.49E-02	7.71
*CD44*	-0.11	4.71E-01	10.57
*NES*	-0.44	5.36E-02	8.59
*FZD9*	-0.70	2.00E-01	-1.10
*CD271*			nd
*SSEA-3/4*			nd
**Pluripotency-related genes**
*POU5F1B/OCT4B*	0.06	9.72E-01	-4.84
*NANOG*			nd
*SOX2*			nd
**MSC negative markers**	
*CD34*	-0.99	1.58E-01	2.78
*PTPRC/CD45*	0.00	9.98E-01	-1.57
*CD14*	1.74	8.31E-03	0.62
*CD79A*	-0.53	4.59E-01	-2.58
*HLA-DRA*	0.79	4.62E-01	-5.55
*HLA-DRB1*	0.29	7.52E-01	-4.25
*HLA-DRB5*	0.87	3.56E-01	-4.69
*HLA-DRB6*	1.25	2.92E-01	-5.23

*nd, none detected.*

Chromosomal abnormalities and malignant transformation of cultured MSCs have been reported. To address this critical safety issue, we searched for transcripts involved in telomerase function. We found high transcript levels of important telomere stability genes *TERC* (Telomerase RNA Component), *TERF1* and *2* (Telomeric Repeat Binding Factor 1 and 2), *TINF2* (TERF1 Interacting Nuclear Factor 2), *TERF2IP* (TERF2 Interacting Protein), *TNKS* (Tankyrase) and *POT1* (Protection of Telomere 1) which were not inhibited upon A83-01 treatment (NCBI account). These transcripts encode for proteins regulating telomere length and protect cells from chromosomal damage, indicating the stability of our cultured eMSCs. *hTERT* telomerase was very lowly or not expressed, suggesting low tumourigenic potential. We also considered expression of the pluripotency factors *OCT4B*, *NANOG*, and *SOX2* after A83-01 treatment (Table 2). These transcripts were not detected or were at extremely low levels in both control and A83-01-treated eMSCs. Mesodermal markers (*SNAI1*, *SOX17*) or the endodermal marker (E-cadherin) involved in mesenchymal epithelial transition were not upregulated. Together these data infer genetic stability and little likelihood of transformation into an immortal state, providing confidence in the safety of cultured-expanded A83-01-treated eMSCs for future clinical use.

Cell proliferation genes including, *ISG20*, *PGF*, and *ACSL5* were upregulated in A83-01-treated eMSCs (Figure 4B) while the anti-proliferative genes *GLI*, *EDNRB*, and *GREM1* were inhibited (Figure 4C), confirming differences in the proliferative phenotype between A83-01-treated and control eMSCs reported previously (Gurung et al., 2015b). We also showed downregulation of several fibroblast-related genes; *PRICKLE1*, *SNCAIP*, and *PDGFC* (Figure 4D), and pro-apoptotic genes including *ROS1* and *TP53I3* and several increased anti-apoptotic genes; *FAIM2*, *NFKBIA*, *ENPP2*, and *SEPTA* in A83-01 treated-eMSCs compared to control (Figure 4E). Taken together, this gene profile suggests that A83-01 confers a proliferative and anti-apoptotic phenotype during culture expansion of eMSCs, ensuring substantial capacity for scale up necessary for clinical translation.

### A83-01 Has no Effect on ISCT MSC Surface Marker Genes

While eMSCs have the standard MSC surface marker phenotype recommended by the International Society for Cellular Therapy (ISCT), we previously found they were also present on cultured endometrial fibroblasts and were not sensitive to changes in culture conditions (Rajaraman et al., 2013). To determine if A83-01 modulated MSC phenotypic markers, we compared expression levels of *CD90*, *CD73*, and *CD105* and found transcripts highly and similarly expressed in both groups (Table 2). As expected the negative ISCT markers *CD34*, *CD45*, *CD14*, *CD79a*, *HLA-DRA*, *HLA-DRB1*, *HLA-DRB5*, and *HLA-DRB6* had low transcript levels in both A83-01-treated and control eMSCs (Table 2). We also searched for MSC-defining marker transcripts previously suggested for purifying MSCs from bone marrow and other sources (Lv et al., 2014). *CD49f*, *CD166*, *CD44*, and *NES* transcripts were highly and similarly expressed in A83-01-treated and control eMSCs (Table 2). *CD271*, *SSEA3/4*, and *FZD9* transcripts were either not detected or expressed at very low levels. Our data further confirms that the standard MSC markers, also expressed by stromal fibroblasts (Bianco et al., 2013), are poor defining markers of the tissue MSC phenotype. Mesodermal differentiation markers for osteogenesis (*RUNX1*) and chondrogenesis (*COL10A1*) were downregulated by A83-01 treatment but there was no difference in the adipogenic marker, *FABP4*. Downregulation of mesodermal lineage markers suggest that A83-01-treated eMSCs are higher up in the hierarchical model of MSC differentiation.

### Comparative Analysis of Fibroblast-Related Genes in A83-01-Treated eMSCs With the Published Literature

A recent microarray study comparing lineage-associated genes between freshly isolated and FAC-sorted CD140b^+^CD146^+^ eMSCs and CD140b^+^CD146^-^ endometrial fibroblasts and subsequent long-term cultures highlighted the spontaneous differentiation of eMSCs to fibroblasts during prolonged culture (Barragan et al., 2016; Gargett and Gurung, 2016). Extensive culture of enriched eMSCs led to downregulation of 81% of eMSC lineage genes and upregulation of 55% of fibroblasts-related genes showing gene profile similarity for eMSCs and fibroblast cultures (Barragan et al., 2016). Comparison of this list of endometrial fibroblast-associated genes in eMSCs with our SUSD2^+^ eMSCs cultured in A83-01 medium showed significant downregulation of 19 fibroblast-related genes and presence of many genes common to freshly isolated CD140b^+^CD146^+^ eMSCs, (Table 3), indicating that A83-01-treated eMSCs are similar to their primary tissue counterparts. This comparison further supports the hypothesis that eMSCs differentiate into stromal fibroblasts during culture, losing their potency and that A83-01 prevents this process and maintains an undifferentiated MSC-state.

**Table 3:** Comparison of fibroblast-related genes in our study to Barragan et al. (2016).

		Fold change log_2_		Barragan et al., 2016,	
Gene description	Gene	(Control vs. A83-01)		FACS sorted	Late culture
Adenylate cyclas+A3:F39e 2 (brain)	*ADCY2*	-1.09	Down	Down	NS
Calsyntenin 2	*CLSTN2*	-3.05	Down	Down	NS
CD24	*CD24*	-3.87	Down	Down	Down
Deiodinase, iodothyronine, type II	*DIO2*	-2.47	Down	Down	NS
Fibroblast growth factor 10	*FGF10*	-1.57	Down	Down	Down
Hedgehog interacting protein	*HHIP*	-2.34	Down	Down	Down
Insulin-like growth factor 1 (somatomedin C)	*IGF1*	-1.53	Down	Down	Down
Insulin-like growth factor 2 (somatomedin A)	*IGF2*	-2.18	Down	Down	NS
Lysyl oxidase	*LOX*	-1.89	Down	Down	Up
Leucine rich repeat containing 17	*LRRC17*	-2.85	Down	Down	NS
Matrix metallopeptidase 10 (stromelysin 2)	*MMP10*	-4.8	Down	Down	Up
Wingless-type MMTV integration site family member 2	*WNT2*	-2.32	Down	Down	NS
Wingless-type MMTV integration site family, member 5A	*WNT5A*	-1.96	Down	Down	Up
Roundabout, axon guidance receptor, homolog 2 (Drosophila)	*ROBO2*	-1.19	Down	Down	NS
Secreted frizzled-related protein 4	*SFRP4*	-1.69	Down	Down	Down
Synuclein, alpha interacting protein	*SNCAIP*	-1.95	Down	Down	NS
Solute carrier family 27 member 6	*SLC27A6*	-1.48	Down	Down	Down
Podoplanin	*PDPN*	-1.41	Down	Down	NS
Platelet derived growth factor C	*PDGFC*	-1.83	Down	Down	Up
Calbindin 2	*CALB2*	0.02	NS	Down	NS
Fibroblast growth factor 9	*FGF9*	-0.5	NS	Down	NS
Fanconi anemia, complementation group L	*FANCL*	0.41	NS	Down	NS
Gap junction protein, alpha 1, 43 kDa	*GJA1*	-0.04	NS	Down	NS
Integrin, beta-like 1 (with EGF-like repeat domains)	*ITGBL1*	0.46	NS	Down	NS
Phospholipase A2 receptor 1, 180 kDa	*JAZF1*	0.17	NS	Down	NS
Lymphocyte cytosolic protein 1 (L-plastin)	*LCP1*	0.99	NS	Down	NS
Leucine carboxyl methyltransferase 1	*LCMT1*	0.01	NS	Down	NS
Melanoma associated antigen (mutated) 1-like 1	*MUM1L1*	-0.85	NS	Down	NS
5′-nucleotidase, ecto (CD73)	*NT5E*	0.65	NS	Down	NS
Pregnancy-associated plasma protein A	*PAPPA*	-0.75	NS	Down	NS
Platelet derived growth factor D	*PDGFD*	-0.09	NS	Down	NS
Transmembrane protein 45A	*TMEM45A*	-0.08	NS	Down	NS
Microfibrillar-associated protein 4	*MFAP4*	0.33	NS	Down	Up
DEAD (Asp-Glu-Ala-Asp) box polypeptide 60-like	*DDX60L*	0.32	NS	Down	NS
Carboxymethylenebutenolidase homolog	*CMBL*	1.01	UP	Down	NS
Membrane metalloendopeptidase	*MME*	1.01	UP	Down	Up
Fibroblast growth factor 7	*FGF7*	1.66	UP	Down	NS

*Down, downregualted; Up, upregulated; NS, not significant*.

## Discussion

Our RNA-sequencing analysis of late passage eMSCs cultured with and without A83-01, a TGF-β receptor inhibitor identified numerous DEGs, generating a transcriptome profile that serves as a reference for culture-expanded MSCs. Our study revealed a unique profile of DEGs in A83-01-treated eMSCs associated with MSC potency, angiogenesis, anti-fibrosis, chemotaxis and immune-regulation, supporting our previous findings that A83-01 maintains functionally undifferentiated eMSCs by inhibiting TGF-βR signaling (Gurung et al., 2015b). Using multiple approaches we provide further molecular and functional evidence that A83-01-treated eMSCs have greater angiogenic potential, improved anti-fibrotic and immunoregulatory properties than control cultures. Our RNA-seq analysis also generated important evidence that eMSCs may be capable of producing exosomes and their unique cargo, suggesting their potential exploitation in generating an “off the shelf” product for regenerative medicine. Finally, we demonstrated a distinct difference at the transcriptional level between eMSCs and fibroblasts, suggesting that the simple use of a small molecule during culture expansion generates MSCs with a transcriptome and some properties vital for their potential use in cell-based therapies. It also provides molecular knowledge increasing understanding of possible MSC functions *in vivo* and their potential therapeutic mechanisms.

Tissue repair without fibrosis is an important goal of regenerative medicine, for which neo-vascularisation and deposition of appropriate ECM are key components. MSCs secrete a wide range of factors favoring healing without fibrosis (Tolar et al., 2010). Our data on A83-01-treated eMSC supports their potential capacity for tissue regeneration by promoting appropriate deposition and organization of ECM and collagen fibrils. A high collagen I/III ratio is a hallmark of fibrosis. Herein, we showed that A83-01 downregulated *COL1A1* (also COL1A1) maintaining a low collagen I/III ratio favoring scarless healing. Although TGF-β is the primary driver of fibrosis, scarless healing depends on the relative expression of its isomers; predominant TGF-β3 with decreasing TGF-β1/2 for minimizing scars (Jones et al., 2006). No change in *TGF*-β*1* expression but downregulated *TGF*-β*2* and upregulated *TGF*-β*3* suggest that A83-01-treated eMSCs have a TGF-β profile supporting scarless healing. Other factors associated with minimal fibrosis are CXC ligands *CXCL12*, *SDF1*, *CXCL2/3/16* and *CCL8* and, fibrosis regulators *HGF*, *MMPs*, *COL1A1*, *FN1*, and *CTGF*. These factors are favorably regulated in A83-01-treated eMSCs compared to control. Similarly, all components of PGE2 synthesis, which prevents myofibroblast differentiation and exerts anti-fibrotic effects were upregulated following A83-01 treatment. Deficiency of PGE2 promotes fibrogenesis in various *in vitro* models and in pulmonary, renal and cardiovascular fibrosis (Dackor et al., 2011; Schippers et al., 2017). MSCs isolated from chronic allergen-induced asthma in mice, and in obliterative bronchiolitis of post-lung transplantation patients with classic tissue fibrosis, lack PGE2 synthesis (Stumm et al., 2011; Walker et al., 2012). Taken together, our data provide further evidence that A83-01-treated eMSCs have the hallmark transcriptome that may promote scarless tissue repair, reminiscent of their *in vivo* function.

Gene profiling revealed that eMSCs have an angiogenic phenotype. HGF is a potent mitogen for hepatocytes and endothelial cells, promoting angiogenesis, in addition to its anti-fibrotic properties (Bussolino et al., 1992). VCAM1, a cell adhesion molecule facilitating vessel growth and HPSE (heparanase) improved ischemic parameters in rats and mice (Hu et al., 2015) were additional angiogenic factors upregulated by A83-01. Our gene profiling combined with multiplex protein and *in vitro* angiogenesis assays, demonstrated that A83-01-treated eMSCs had an angiogenic phenotype, secreting increased pro-angiogenic and decreased anti-angiogenic proteins. Not only did A83-01-treated eMSC CM increased HUVEC tube formation, but A83-01-treated eMSCs themselves formed tubes. Taken together, the pro-angiogenic phenotype and properties of A83-01-treated eMSCs highlight their potential beneficial use in regenerative medicine.

A wide variety of cytokines were induced in A83-01-treated eMSCs. Cytokines regulate cell rolling, trafficking and leucocyte recruitment, activation and differentiation (Bachmann et al., 2006). Although cytokines have been classified into pro-inflammatory and anti-inflammatory categories, they have additional functions depending upon the target cells, cytokine concentration and microenvironment (Cavaillon, 2001). For example, IL-33 (alarmin), one of the most upregulated genes in A83-01-treated eMSCs, alerts the immune system to tissue damage but also activates endothelial cells and induces angiogenesis (Choi et al., 2009). Similarly, genes involved in the TNF signaling pathway are vital for recruiting immune cells and in anti-inflammatory signaling. *TNFAIP3*, upregulated in A83-01-treated eMSCs, is a potent anti-inflammatory signaling molecule that suppresses inflammatory signals through inhibition of NF-kB. IFN-γ signaling provokes synthesis of chemokines and inducible nitric oxide synthase in MSCs (Ren et al., 2008). This dual action promotes T-cell chemotaxis toward MSCs, while MSC-secreted nitric oxide suppresses their responses leading to immunosuppression. Several Toll-like receptors expressed by MSCs (Delarosa et al., 2012) are surface proteins of innate immune cells that recognize pathogens. A83-01 enhanced eMSC *TLR2* and *TLR3* but not *TLR4* expressions. Depending on which TLR is activated, MSCs can change into a pro- or anti-inflammatory phenotype. TLR2/4 primed MSCs mediate pro-inflammatory processes, inducing secretion of GM-CSF which maintains macrophages in an M1 pro-inflammatory phenotype while activation of MSCs through TLR3 increases PGE2 and IDO synthesis, priming them to an anti-inflammatory phenotype (Wang et al., 2014; Le Blanc and Davies, 2015). In A83-01-treated eMSCs, in addition to the favorable differential expression of TLRs, GM-CSF secretion was inhibited and transcriptome data shows an increase in transcripts involved in PGE2 synthesis compared to the controls thus indicating an environment favorable for conversion of M1 pro-inflammatory to M2 anti-inflammatory macrophages. TLR3 signaling in MSCs enhances potency through induction of immunosuppressive T regulatory (Treg) cells by direct cell contact with naïve CD4 cells (Rashedi et al., 2017). TGF-β signaling induces naïve CD4 cell differentiation into CD4^+^Foxp3^+^Tregs, conducive in maintaining immune tolerance (Chen and Konkel, 2010). TGF-β1 is the most important TGF-β isoform in maintaining and regulating homeostasis of Tregs (Marie et al., 2005). A83-01 did not inhibit eMSC’s autocrine secretion of TGF-β1, therefore their immunosuppressive effect in regulating Tregs via TGF-β1 likely remains intact. A83-01-treated eMSCs also upregulated transcripts of immune regulators, *SOCS3* and *SOD3*. SOCS3 inhibits Th17 cell differentiation into Tregs while SOD3, a potent antioxidant, protects cells from free radicals and activated oxygen species (Nightingale et al., 2012). SOD3 scavenges reactive oxygen species, protecting tissues from damage and dampens the inflammatory cascade (Nightingale et al., 2012). Overall our findings provide important insight into how A83-01 modulates eMSCs to promote intrinsic anti-inflammatory, antioxidant and immunomodulatory properties, highly desirable for their use in clinical translation.

The complement system has been recently linked to the rapid clearance of infused MSCs due to Instant Blood Mediated Inflammatory Reaction (IBMIR) (Moll et al., 2012). Genes involved in activation of complement and coagulation regulators were upregulated in A83-01-treated eMSCs compared to control. Plasma- and membrane-bound regulators of complement genes were also differentially expressed. The gene profiles of the complement and coagulation pathways suggest that with the overall balance of the activators, inhibitors and regulators, A83-01-treated eMSCs would likely escape IBMIR if used intravenously in an allogeneic or autologous setting. Thereby, intravenously administered A83-01-treated eMSCs are potentially self-protected and may survive and engraft thus making them therapeutically useful.

Maintenance of MSC potency and self-renewal during culture expansion is vital to maintain the MSC phenotype, potency and prolong their efficacy *in vivo* for regenerative medicine purposes (Chen et al., 2014). Similar to previous studies based on functional assays and hierarchical clustering of fresh eMSCs and endometrial fibroblast, our transcriptome and pathway analyses further demonstrated high expression of the classic MSC genes; *CD90*, *CD73*, and *CD105* (Gurung et al., 2015a; Barragan et al., 2016) and herein we provided further evidence that eMSCs undergo culture-induced differentiation into fibroblasts thereby losing potency, similar to other sources of MSCs (Chen et al., 2014; Turinetto et al., 2016). An exciting finding in our study was upregulation of MSC potency-related genes (Samsonraj et al., 2015), an important advance for bringing eMSCs into the clinic. The downregulation of fibroblast-related genes in A83-01-treated eMSCs and the absence of pluripotency-related transcripts, as well as our previous study where no macroscopic masses were generated when these cells were transplanted sub-renally in immunocompromised mice (Gurung et al., 2018) indicates their safety, important as there are reports describing MSC instability and spontaneous transformation during culture expansion (Rosland et al., 2009).

Accumulating evidence in the MSC field suggests that membrane-enclosed extracellular-vesicles may exert the MSC paracrine effect. EVs carry important cargo containing proteins, microRNAs, bioactive lipids and other regulatory molecules (Wang et al., 2017; Blazquez et al., 2018). Of interest are the 40–100 nm exosomes (Colombo et al., 2014), which have emerged as important intercellular communicators. Our comprehensive RNA-seq analysis of eMSCs identified transcripts/markers of the top 100 exosome genes listed in Exocarta (Simpson et al., 2012). Furthermore, we identified typical exosome cargo transcripts including microRNAs. Of importance was the expression of *HLA-G* exosomal cargo, a non-classical HLA transcript, whose gene product has strong immunosuppressive properties (Ding et al., 2016). Although *HLA-G* expression is restricted to the maternal-fetal interface, preventing embryo rejection during implantation, its expression has been identified in umbilical cord stroma-derived MSCs but not bone marrow or placental-derived MSCs (Ding et al., 2016). The strong immunosuppressive activity of HLA-G indicates the potential utility of eMSC in allogeneic therapy. Our data on exosomes from A83-01-treated eMSCs opens the way to examine their potential use in regenerative medicine as an “off the shelf” cell-derived therapy.

## Conclusion

Our data comprehensively describes the gene expression profiles of human eMSCs cultured with and without a small molecule TGF-βR inhibitor, A83-01. We showed A83-01 enhanced angiogenic properties of eMSCs as well as potentially maintaining their potency. The transcriptional and protein profiles of A83-01-treated eMSCs also suggest they will have enhanced anti-fibrotic, anti-inflammatory and immunoregulatory properties over and above those we have previously shown *in vivo* and *in vitro* for untreated eMSCs (Ulrich et al., 2014; Darzi et al., 2018). For the first time, we showed eMSCs may be a source of exosomes for cell communication. These studies suggest the transcriptome and enhanced properties of A83-01-treated eMSCs increase their utility for future cell and regenerative therapies.

## Author Contributions

SG: experimental design, collection and assembly of data, data analysis and interpretation, manuscript writing, and final approval of manuscript. SW: assembly of data, data analysis and interpretation, and final approval of manuscript. JD: assisted with data collection, manuscript editing, and final approval of manuscript. JW: study design, manuscript editing, and final approval of manuscript. CG: conception and design, financial support, manuscript editing, and final approval of manuscript.

## Conflict of Interest Statement

The authors declare that the research was conducted in the absence of any commercial or financial relationships that could be construed as a potential conflict of interest.
