# Supplementary material for: The Transcriptome of Human Endometrial Mesenchymal Stem Cells Under TGFβR Inhibition Reveals Improved Potential for Cell-Based Therapies
Source: Front Cell Dev Biol. 2018 Dec 4;6:164. doi: 10.3389/fcell.2018.00164 (PMC6288489; doi:10.3389/fcell.2018.00164)
Supplement: Supplementary file 1 [file Data_Sheet_1.docx]

Supplementary Material

**Transcriptome sequencing of human endometrial mesenchymal stem cells under TGF-βR inhibition reveals a potential for cell-based therapy**

**Shanti. Gurung^1,2^, Sarah. Williams^3^, James. A. Deane^1,2^, Jerome. A. Werkmeister^1,2^, Caroline. E. Gargett^1,2^,^*^**

*** Correspondence:**Caroline E.Gargett
caroline.gargett@hudson.org.au

**Supplementary table 1**

**Genes significantly (FDR<0.01) differentially upregulated (Fold change >2) in control and A83-01-treated eMSCs compared to control**

| EnsemblGeneID | Gene | Fold change log_2_ | | FDR | Ave Expr |
| --- | --- | --- | --- | --- | --- |
|  |  | **Control** | **A83-01** |  |  |
| ENSG00000109610 | *SOD3* | 0 | 2.24 | 1.65E-06 | 5.21 |
| ENSG00000104332 | *SFRP1* | 0 | 2.93 | 1.65E-06 | 8.86 |
| ENSG00000168079 | *SCARA5* | 0 | 6.54 | 5.68E-06 | 0.34 |
| ENSG00000108381 | *ASPA* | 0 | 3.01 | 6.68E-06 | 1.48 |
| ENSG00000160801 | *PTH1R* | 0 | 1.92 | 9.26E-06 | 1.03 |
| ENSG00000140945 | *CDH13* | 0 | 1.37 | 1.28E-05 | 7.35 |
| ENSG00000188783 | *PRELP* | 0 | 2.61 | 1.65E-05 | 5.22 |
| ENSG00000111913 | *FAM65B* | 0 | 2.57 | 1.73E-05 | 4.24 |
| ENSG00000107738 | *C10orf54* | 0 | 1.41 | 1.89E-05 | 5.95 |
| ENSG00000145147 | *SLIT2* | 0 | 2.05 | 1.89E-05 | 4.33 |
| ENSG00000133321 | *RARRES3* | 0 | 2.25 | 2.16E-05 | 3.69 |
| ENSG00000197971 | *MBP* | 0 | 2.13 | 2.59E-05 | 4.10 |
| ENSG00000168062 | *BATF2* | 0 | 1.72 | 2.99E-05 | 2.47 |
| ENSG00000163975 | *MELTF* | 0 | 1.89 | 3.09E-05 | 2.72 |
| ENSG00000155093 | *PTPRN2* | 0 | 1.64 | 3.12E-05 | 2.63 |
| ENSG00000105825 | *TFPI2* | 0 | 3.89 | 3.18E-05 | 6.93 |
| ENSG00000167779 | *IGFBP6* | 0 | 2.12 | 3.41E-05 | 8.63 |
| ENSG00000171873 | *ADRA1D* | 0 | 2.06 | 3.41E-05 | 4.10 |
| ENSG00000100342 | *APOL1* | 0 | 1.88 | 3.41E-05 | 6.02 |
| ENSG00000160932 | *LY6E* | 0 | 1.10 | 3.53E-05 | 9.56 |
| ENSG00000197142 | *ACSL5* | 0 | 2.15 | 3.53E-05 | 3.36 |
| ENSG00000069702 | *TGFBR3* | 0 | 2.65 | 3.53E-05 | 4.27 |
| ENSG00000159640 | *ACE* | 0 | 1.66 | 3.53E-05 | 3.29 |
| ENSG00000148671 | *ADIRF* | 0 | 2.83 | 3.58E-05 | 3.23 |
| ENSG00000028137 | *TNFRSF1B* | 0 | 2.77 | 4.62E-05 | 2.01 |
| ENSG00000115380 | *EFEMP1* | 0 | 3.76 | 4.62E-05 | 1.06 |
| ENSG00000147465 | *STAR* | 0 | 3.56 | 4.62E-05 | 0.66 |
| ENSG00000253304 | *TMEM200B* | 0 | 1.16 | 4.62E-05 | 4.47 |
| ENSG00000271447 | *MMP28* | 0 | 3.99 | 4.62E-05 | -1.01 |
| ENSG00000185052 | *SLC24A3* | 0 | 2.22 | 4.83E-05 | 0.54 |
| ENSG00000229847 | *EMX2OS* | 0 | 1.13 | 5.13E-05 | 4.63 |
| ENSG00000172296 | *SPTLC3* | 0 | 3.72 | 5.20E-05 | -1.27 |
| ENSG00000135472 | *FAIM2* | 0 | 1.64 | 5.65E-05 | 3.48 |
| ENSG00000134470 | *IL15RA* | 0 | 1.96 | 5.83E-05 | 2.98 |
| ENSG00000168477 | *TNXB* | 0 | 3.75 | 5.92E-05 | 5.27 |
| ENSG00000080031 | *PTPRH* | 0 | 1.84 | 6.29E-05 | 2.22 |
| ENSG00000136960 | *ENPP2* | 0 | 2.73 | 6.38E-05 | 0.31 |
| ENSG00000109819 | *PPARGC1A* | 0 | 2.82 | 6.44E-05 | 0.81 |
| ENSG00000237125 | *HAND2-AS1* | 0 | 1.30 | 6.89E-05 | 4.52 |
| ENSG00000151748 | *SAV1* | 0 | 1.23 | 7.04E-05 | 6.00 |
| ENSG00000173376 | *NDNF* | 0 | 4.15 | 7.33E-05 | 0.49 |
| ENSG00000184371 | *CSF1* | 0 | 1.81 | 7.52E-05 | 5.40 |
| ENSG00000234883 | *MIR155HG* | 0 | 2.05 | 7.52E-05 | 2.19 |
| ENSG00000104361 | *NIPAL2* | 0 | 1.04 | 7.90E-05 | 5.53 |
| ENSG00000162882 | *HAAO* | 0 | 1.85 | 8.15E-05 | -0.01 |
| ENSG00000074410 | *CA12* | 0 | 3.41 | 8.18E-05 | 1.46 |
| ENSG00000107984 | *DKK1* | 0 | 1.29 | 8.30E-05 | 5.16 |
| ENSG00000164342 | *TLR3* | 0 | 2.64 | 9.28E-05 | 1.04 |
| ENSG00000134352 | *IL6ST* | 0 | 1.15 | 9.43E-05 | 8.83 |
| ENSG00000158473 | *CD1D* | 0 | 3.01 | 9.83E-05 | -1.43 |
| ENSG00000079257 | *LXN* | 0 | 1.57 | 1.03E-04 | 5.59 |
| ENSG00000122035 | *RASL11A* | 0 | 2.02 | 1.14E-04 | 3.05 |
| ENSG00000196616 | *ADH1B* | 0 | 4.08 | 1.14E-04 | 2.80 |
| ENSG00000100307 | *CBX7* | 0 | 1.34 | 1.14E-04 | 4.59 |
| ENSG00000102524 | *TNFSF13B* | 0 | 1.78 | 1.14E-04 | 1.04 |
| ENSG00000077092 | *RARB* | 0 | 1.74 | 1.16E-04 | 2.03 |
| ENSG00000197442 | *MAP3K5* | 0 | 1.24 | 1.21E-04 | 6.40 |
| ENSG00000165092 | *ALDH1A1* | 0 | 1.62 | 1.21E-04 | 6.55 |
| ENSG00000121797 | *CCRL2* | 0 | 2.26 | 1.26E-04 | -0.82 |
| ENSG00000137727 | *ARHGAP20* | 0 | 1.86 | 1.29E-04 | 2.66 |
| ENSG00000119535 | *CSF3R* | 0 | 3.83 | 1.30E-04 | -2.70 |
| ENSG00000005059 | *MCUB* | 0 | 1.25 | 1.31E-04 | 3.69 |
| ENSG00000188641 | *DPYD* | 0 | 1.11 | 1.32E-04 | 5.16 |
| ENSG00000172935 | *MRGPRF* | 0 | 1.14 | 1.35E-04 | 6.62 |
| ENSG00000118849 | *RARRES1* | 0 | 3.04 | 1.37E-04 | 1.94 |
| ENSG00000168016 | *TRANK1* | 0 | 1.37 | 1.37E-04 | 5.89 |
| ENSG00000168899 | *VAMP5* | 0 | 1.13 | 1.37E-04 | 5.06 |
| ENSG00000120149 | *MSX2* | 0 | 1.47 | 1.38E-04 | -0.16 |
| ENSG00000166292 | *TMEM100* | 0 | 2.22 | 1.39E-04 | 4.35 |
| ENSG00000184922 | *FMNL1* | 0 | 2.16 | 1.41E-04 | 3.53 |
| ENSG00000128284 | *APOL3* | 0 | 1.67 | 1.41E-04 | 4.27 |
| ENSG00000120915 | *EPHX2* | 0 | 1.16 | 1.44E-04 | 1.71 |
| ENSG00000184349 | *EFNA5* | 0 | 1.68 | 1.44E-04 | 3.48 |
| ENSG00000137033 | *IL33* | 0 | 4.29 | 1.45E-04 | 5.85 |
| ENSG00000143382 | *ADAMTSL4* | 0 | 2.48 | 1.46E-04 | 4.68 |
| ENSG00000185885 | *IFITM1* | 0 | 1.61 | 1.47E-04 | 7.07 |
| ENSG00000157168 | *NRG1* | 0 | 1.96 | 1.49E-04 | 5.37 |
| ENSG00000156486 | *KCNS2* | 0 | 3.68 | 1.56E-04 | -0.01 |
| ENSG00000183242 | *WT1-AS* | 0 | 2.48 | 1.57E-04 | -0.46 |
| ENSG00000185201 | *IFITM2* | 0 | 1.20 | 1.61E-04 | 7.48 |
| ENSG00000164236 | *ANKRD33B* | 0 | 2.06 | 1.62E-04 | 0.39 |
| ENSG00000267121 | *CTD-2020K17.1* | 0 | 2.61 | 1.63E-04 | 1.01 |
| ENSG00000088881 | *EBF4* | 0 | 1.06 | 1.63E-04 | 3.83 |
| ENSG00000166949 | *SMAD3* | 0 | 1.02 | 1.69E-04 | 6.85 |
| ENSG00000128340 | *RAC2* | 0 | 1.04 | 1.75E-04 | 5.05 |
| ENSG00000142089 | *IFITM3* | 0 | 1.22 | 1.75E-04 | 9.27 |
| ENSG00000165030 | *NFIL3* | 0 | 1.06 | 1.84E-04 | 4.78 |
| ENSG00000115457 | *IGFBP2* | 0 | 2.03 | 1.85E-04 | 8.18 |
| ENSG00000144152 | *FBLN7* | 0 | 1.27 | 1.85E-04 | 3.03 |
| ENSG00000101197 | *BIRC7* | 0 | 4.58 | 1.85E-04 | -3.45 |
| ENSG00000136040 | *PLXNC1* | 0 | 1.36 | 1.85E-04 | 2.21 |
| ENSG00000127920 | *GNG11* | 0 | 1.60 | 1.85E-04 | 8.46 |
| ENSG00000025434 | *NR1H3* | 0 | 1.32 | 1.85E-04 | 4.40 |
| ENSG00000196639 | *HRH1* | 0 | 1.16 | 1.86E-04 | 4.37 |
| ENSG00000184785 | *SMIM10* | 0 | 1.15 | 1.86E-04 | 4.14 |
| ENSG00000164107 | *HAND2* | 0 | 1.18 | 2.04E-04 | 5.61 |
| ENSG00000259275 | *RP11-522B15.3* | 0 | 1.37 | 2.09E-04 | 2.36 |
| ENSG00000134443 | *GRP* | 0 | 2.49 | 2.14E-04 | 1.12 |
| ENSG00000173083 | *HPSE* | 0 | 2.29 | 2.17E-04 | 0.48 |
| ENSG00000154102 | *C16orf74* | 0 | 1.29 | 2.18E-04 | 2.07 |
| ENSG00000004468 | *CD38* | 0 | 4.12 | 2.25E-04 | -1.78 |
| ENSG00000165171 | *WBSCR27* | 0 | 1.77 | 2.30E-04 | 0.87 |
| ENSG00000151322 | *NPAS3* | 0 | 1.19 | 2.32E-04 | 2.54 |
| ENSG00000170425 | *ADORA2B* | 0 | 2.58 | 2.32E-04 | 2.95 |
| ENSG00000004776 | *HSPB6* | 0 | 1.86 | 2.32E-04 | 5.34 |
| ENSG00000179403 | *VWA1* | 0 | 1.60 | 2.42E-04 | 3.46 |
| ENSG00000154175 | *ABI3BP* | 0 | 1.70 | 2.44E-04 | 3.20 |
| ENSG00000163053 | *SLC16A14* | 0 | 1.48 | 2.45E-04 | 2.19 |
| ENSG00000100505 | *TRIM9* | 0 | 1.57 | 2.50E-04 | 0.28 |
| ENSG00000041353 | *RAB27B* | 0 | 2.94 | 2.58E-04 | 1.93 |
| ENSG00000137818 | *RPLP1* | 0 | 1.10 | 2.58E-04 | 8.92 |
| ENSG00000147576 | *ADHFE1* | 0 | 1.47 | 2.61E-04 | 1.43 |
| ENSG00000160712 | *IL6R* | 0 | 1.45 | 2.72E-04 | 3.67 |
| ENSG00000172201 | *ID4* | 0 | 2.13 | 2.72E-04 | -0.82 |
| ENSG00000126561 | *STAT5A* | 0 | 1.08 | 2.72E-04 | 2.80 |
| ENSG00000152804 | *HHEX* | 0 | 1.73 | 2.74E-04 | 0.44 |
| ENSG00000169418 | *NPR1* | 0 | 2.74 | 2.78E-04 | -1.27 |
| ENSG00000117266 | *CDK18* | 0 | 2.39 | 2.84E-04 | 1.05 |
| ENSG00000075651 | *PLD1* | 0 | 1.35 | 2.93E-04 | 4.79 |
| ENSG00000142046 | *TMEM91* | 0 | 1.07 | 2.93E-04 | 2.37 |
| ENSG00000159713 | *TPPP3* | 0 | 1.86 | 2.99E-04 | 1.44 |
| ENSG00000179604 | *CDC42EP4* | 0 | 1.05 | 3.00E-04 | 6.21 |
| ENSG00000182118 | *FAM89A* | 0 | 1.43 | 3.01E-04 | 1.42 |
| ENSG00000133687 | *TMTC1* | 0 | 1.77 | 3.05E-04 | 6.08 |
| ENSG00000164920 | *OSR2* | 0 | 2.64 | 3.25E-04 | 6.06 |
| ENSG00000013293 | *SLC7A14* | 0 | 1.81 | 3.28E-04 | 0.73 |
| ENSG00000131094 | *C1QL1* | 0 | 1.66 | 3.28E-04 | 1.47 |
| ENSG00000112297 | *AIM1* | 0 | 1.43 | 3.32E-04 | 5.01 |
| ENSG00000253276 | *CCDC71L* | 0 | 1.38 | 3.35E-04 | 6.14 |
| ENSG00000110881 | *ASIC1* | 0 | 1.17 | 3.35E-04 | 2.72 |
| ENSG00000161249 | *DMKN* | 0 | 3.41 | 3.36E-04 | -0.30 |
| ENSG00000106258 | *CYP3A5* | 0 | 2.03 | 3.36E-04 | 0.75 |
| ENSG00000183762 | *KREMEN1* | 0 | 1.41 | 3.37E-04 | 4.15 |
| ENSG00000151090 | *THRB* | 0 | 1.20 | 3.39E-04 | 4.93 |
| ENSG00000204991 | *SPIRE2* | 0 | 1.47 | 3.50E-04 | 3.43 |
| ENSG00000090539 | *CHRD* | 0 | 1.34 | 3.54E-04 | 4.14 |
| ENSG00000005379 | *TSPOAP1* | 0 | 1.11 | 3.56E-04 | -0.59 |
| ENSG00000116667 | *C1orf21* | 0 | 1.41 | 3.56E-04 | 5.09 |
| ENSG00000094963 | *FMO2* | 0 | 2.13 | 3.60E-04 | 0.77 |
| ENSG00000135218 | *CD36* | 0 | 2.19 | 3.60E-04 | 3.30 |
| ENSG00000154262 | *ABCA6* | 0 | 1.54 | 3.61E-04 | 5.51 |
| ENSG00000004399 | *PLXND1* | 0 | 1.04 | 3.62E-04 | 7.07 |
| ENSG00000100906 | *NFKBIA* | 0 | 1.32 | 3.64E-04 | 6.15 |
| ENSG00000236562 | *RP11-396M11.1* | 0 | 1.79 | 3.64E-04 | -0.07 |
| ENSG00000261625 | *RP11-554A11.4* | 0 | 1.72 | 3.65E-04 | 2.25 |
| ENSG00000100739 | *BDKRB1* | 0 | 1.58 | 3.65E-04 | 3.83 |
| ENSG00000182179 | *UBA7* | 0 | 1.21 | 3.79E-04 | 6.05 |
| ENSG00000101384 | *JAG1* | 0 | 1.76 | 3.81E-04 | 4.82 |
| ENSG00000122176 | *FMOD* | 0 | 1.37 | 3.91E-04 | 6.77 |
| ENSG00000113594 | *LIFR* | 0 | 1.23 | 3.91E-04 | 3.83 |
| ENSG00000048052 | *HDAC9* | 0 | 1.77 | 3.99E-04 | 3.50 |
| ENSG00000182853 | *VMO1* | 0 | 2.33 | 4.03E-04 | -0.43 |
| ENSG00000184937 | *WT1* | 0 | 1.70 | 4.18E-04 | 3.06 |
| ENSG00000134245 | *WNT2B* | 0 | 3.69 | 4.22E-04 | 1.67 |
| ENSG00000219607 | *PPP1R3G* | 0 | 1.41 | 4.31E-04 | 0.90 |
| ENSG00000139364 | *TMEM132B* | 0 | 2.73 | 4.31E-04 | 5.74 |
| ENSG00000122121 | *XPNPEP2* | 0 | 2.54 | 4.33E-04 | -0.50 |
| ENSG00000112096 | *SOD2* | 0 | 2.16 | 4.33E-04 | 7.81 |
| ENSG00000090376 | *IRAK3* | 0 | 2.50 | 4.34E-04 | -0.34 |
| ENSG00000068976 | *PYGM* | 0 | 2.08 | 4.35E-04 | -0.64 |
| ENSG00000169071 | *ROR2* | 0 | 1.25 | 4.39E-04 | 3.07 |
| ENSG00000132205 | *EMILIN2* | 0 | 1.97 | 4.52E-04 | 5.10 |
| ENSG00000128016 | *ZFP36* | 0 | 1.48 | 4.52E-04 | 7.57 |
| ENSG00000140285 | *FGF7* | 0 | 1.66 | 4.52E-04 | 5.74 |
| ENSG00000169116 | *PARM1* | 0 | 2.05 | 4.53E-04 | 3.24 |
| ENSG00000007237 | *GAS7* | 0 | 1.55 | 4.57E-04 | 4.84 |
| ENSG00000196154 | *S100A4* | 0 | 1.07 | 4.59E-04 | 8.40 |
| ENSG00000126709 | *IFI6* | 0 | 1.05 | 4.70E-04 | 7.67 |
| ENSG00000099822 | *HCN2* | 0 | 1.42 | 4.74E-04 | 0.29 |
| ENSG00000124145 | *SDC4* | 0 | 1.12 | 4.75E-04 | 6.99 |
| ENSG00000006747 | *SCIN* | 0 | 1.85 | 4.76E-04 | -0.68 |
| ENSG00000166979 | *EVA1C* | 0 | 2.39 | 4.91E-04 | 1.77 |
| ENSG00000184292 | *TACSTD2* | 0 | 2.44 | 5.12E-04 | -0.27 |
| ENSG00000076770 | *MBNL3* | 0 | 1.37 | 5.14E-04 | 3.65 |
| ENSG00000112414 | *ADGRG6* | 0 | 1.07 | 5.18E-04 | 5.52 |
| ENSG00000132622 | *HSPA12B* | 0 | 1.29 | 5.19E-04 | 1.42 |
| ENSG00000132470 | *ITGB4* | 0 | 2.28 | 5.21E-04 | 2.23 |
| ENSG00000163661 | *PTX3* | 0 | 1.35 | 5.28E-04 | 5.56 |
| ENSG00000162433 | *AK4* | 0 | 1.42 | 5.30E-04 | 0.35 |
| ENSG00000184557 | *SOCS3* | 0 | 1.49 | 5.43E-04 | 6.07 |
| ENSG00000168356 | *SCN11A* | 0 | 2.27 | 5.43E-04 | -0.16 |
| ENSG00000103196 | *CRISPLD2* | 0 | 2.22 | 5.55E-04 | 5.07 |
| ENSG00000171517 | *LPAR3* | 0 | 2.76 | 5.56E-04 | 4.31 |
| ENSG00000139567 | *ACVRL1* | 0 | 2.06 | 5.57E-04 | 4.84 |
| ENSG00000171729 | *TMEM51* | 0 | 1.24 | 5.64E-04 | 5.05 |
| ENSG00000231389 | *HLA-DPA1* | 0 | 1.18 | 5.70E-04 | 2.15 |
| ENSG00000150510 | *FAM124A* | 0 | 1.39 | 5.72E-04 | 3.74 |
| ENSG00000139899 | *CBLN3* | 0 | 1.04 | 5.74E-04 | 3.87 |
| ENSG00000164035 | *EMCN* | 0 | 2.31 | 5.74E-04 | -1.96 |
| ENSG00000003436 | *TFPI* | 0 | 1.37 | 5.74E-04 | 7.60 |
| ENSG00000150594 | *ADRA2A* | 0 | 1.46 | 5.81E-04 | 3.39 |
| ENSG00000119922 | *IFIT2* | 0 | 1.50 | 5.81E-04 | 5.30 |
| ENSG00000106100 | *NOD1* | 0 | 1.01 | 5.82E-04 | 2.76 |
| ENSG00000249242 | *TMEM150C* | 0 | 1.79 | 5.82E-04 | 1.19 |
| ENSG00000117425 | *PTCH2* | 0 | 1.57 | 5.82E-04 | 1.43 |
| ENSG00000111077 | *TNS2* | 0 | 1.24 | 5.88E-04 | 7.12 |
| ENSG00000171223 | *JUNB* | 0 | 1.02 | 5.89E-04 | 8.54 |
| ENSG00000143320 | *CRABP2* | 0 | 1.90 | 5.93E-04 | 8.45 |
| ENSG00000271930 | *RP11-44N12.5* | 0 | 2.16 | 6.04E-04 | -1.53 |
| ENSG00000203797 | *DDO* | 0 | 1.52 | 6.05E-04 | 1.03 |
| ENSG00000104047 | *DTWD1* | 0 | 1.06 | 6.21E-04 | 5.41 |
| ENSG00000150977 | *RILPL2* | 0 | 1.16 | 6.22E-04 | 5.40 |
| ENSG00000185187 | *SIGIRR* | 0 | 1.12 | 6.50E-04 | 3.70 |
| ENSG00000153902 | *LGI4* | 0 | 1.94 | 6.83E-04 | -1.34 |
| ENSG00000124479 | *NDP* | 0 | 2.47 | 6.88E-04 | -0.63 |
| ENSG00000136826 | *KLF4* | 0 | 1.73 | 6.99E-04 | 4.66 |
| ENSG00000125266 | *EFNB2* | 0 | 1.18 | 7.00E-04 | 5.91 |
| ENSG00000169218 | *RSPO1* | 0 | 4.51 | 7.00E-04 | -2.52 |
| ENSG00000105246 | *EBI3* | 0 | 2.12 | 7.00E-04 | 0.66 |
| ENSG00000077942 | *FBLN1* | 0 | 1.56 | 7.06E-04 | 10.42 |
| ENSG00000006740 | *ARHGAP44* | 0 | 1.43 | 7.07E-04 | 1.01 |
| ENSG00000188015 | *S100A3* | 0 | 1.38 | 7.13E-04 | 2.71 |
| ENSG00000183346 | *C10orf107* | 0 | 1.39 | 7.18E-04 | 2.26 |
| ENSG00000131979 | *GCH1* | 0 | 1.88 | 7.19E-04 | 0.16 |
| ENSG00000247317 | *RP11-273G15.2* | 0 | 1.02 | 7.22E-04 | 1.84 |
| ENSG00000185338 | *SOCS1* | 0 | 1.36 | 7.36E-04 | 4.63 |
| ENSG00000167034 | *NKX3-1* | 0 | 1.85 | 7.37E-04 | -0.24 |
| ENSG00000120162 | *MOB3B* | 0 | 2.93 | 7.54E-04 | 1.77 |
| ENSG00000124440 | *HIF3A* | 0 | 1.98 | 7.62E-04 | -1.44 |
| ENSG00000168490 | *PHYHIP* | 0 | 1.50 | 7.67E-04 | 0.56 |
| ENSG00000106633 | *GCK* | 0 | 1.37 | 7.71E-04 | -0.56 |
| ENSG00000177875 | *CCDC184* | 0 | 2.02 | 7.71E-04 | 1.10 |
| ENSG00000136999 | *NOV* | 0 | 2.47 | 7.78E-04 | 5.07 |
| ENSG00000117318 | *ID3* | 0 | 1.14 | 7.78E-04 | 5.87 |
| ENSG00000176485 | *PLA2G16* | 0 | 1.05 | 7.87E-04 | 3.25 |
| ENSG00000061918 | *GUCY1B3* | 0 | 1.63 | 7.87E-04 | 3.53 |
| ENSG00000013619 | *MAMLD1* | 0 | 1.16 | 7.99E-04 | 3.10 |
| ENSG00000147862 | *NFIB* | 0 | 1.95 | 8.07E-04 | -0.13 |
| ENSG00000073756 | *PTGS2* | 0 | 3.67 | 8.07E-04 | 2.97 |
| ENSG00000162407 | *PLPP3* | 0 | 1.50 | 8.07E-04 | 5.01 |
| ENSG00000178031 | *ADAMTSL1* | 0 | 3.74 | 8.12E-04 | 1.04 |
| ENSG00000119640 | *ACYP1* | 0 | 1.13 | 8.20E-04 | 3.98 |
| ENSG00000087510 | *TFAP2C* | 0 | 1.27 | 8.30E-04 | 7.25 |
| ENSG00000239887 | *C1orf226* | 0 | 1.48 | 8.30E-04 | 0.92 |
| ENSG00000204323 | *SMIM5* | 0 | 2.69 | 8.30E-04 | -3.65 |
| ENSG00000196878 | *LAMB3* | 0 | 1.86 | 8.30E-04 | 2.22 |
| ENSG00000173267 | *SNCG* | 0 | 1.71 | 8.37E-04 | 0.21 |
| ENSG00000148344 | *PTGES* | 0 | 3.95 | 8.51E-04 | 4.67 |
| ENSG00000105855 | *ITGB8* | 0 | 1.58 | 8.51E-04 | 4.17 |
| ENSG00000164237 | *CMBL* | 0 | 1.01 | 8.52E-04 | 5.70 |
| ENSG00000181444 | *ZNF467* | 0 | 1.15 | 8.54E-04 | 0.78 |
| ENSG00000172183 | *ISG20* | 0 | 1.32 | 8.54E-04 | 0.90 |
| ENSG00000165272 | *AQP3* | 0 | 1.90 | 8.76E-04 | 2.04 |
| ENSG00000150540 | *HNMT* | 0 | 1.00 | 8.85E-04 | 5.85 |
| ENSG00000168917 | *SLC35G2* | 0 | 1.14 | 8.85E-04 | 3.43 |
| ENSG00000145808 | *ADAMTS19* | 0 | 1.07 | 8.91E-04 | 4.62 |
| ENSG00000138944 | *KIAA1644* | 0 | 2.27 | 8.97E-04 | 1.32 |
| ENSG00000137959 | *IFI44L* | 0 | 1.55 | 9.14E-04 | 3.46 |
| ENSG00000154822 | *PLCL2* | 0 | 1.48 | 9.29E-04 | 2.56 |
| ENSG00000163734 | *CXCL3* | 0 | 2.13 | 9.37E-04 | -0.45 |
| ENSG00000185745 | *IFIT1* | 0 | 1.06 | 9.42E-04 | 6.62 |
| ENSG00000197457 | *STMN3* | 0 | 1.01 | 9.42E-04 | 5.63 |
| ENSG00000003096 | *KLHL13* | 0 | 2.51 | 9.43E-04 | 0.70 |
| ENSG00000143850 | *PLEKHA6* | 0 | 1.12 | 9.43E-04 | 5.52 |
| ENSG00000054219 | *LY75* | 0 | 2.50 | 9.43E-04 | -2.76 |
| ENSG00000243649 | *CFB* | 0 | 1.90 | 9.43E-04 | -0.14 |
| ENSG00000271605 | *MILR1* | 0 | 1.06 | 9.57E-04 | 1.18 |
| ENSG00000105835 | *NAMPT* | 0 | 1.34 | 9.73E-04 | 6.71 |
| ENSG00000161921 | *CXCL16* | 0 | 1.05 | 9.73E-04 | 2.38 |
| ENSG00000135899 | *SP110* | 0 | 1.10 | 9.86E-04 | 2.01 |
| ENSG00000105639 | *JAK3* | 0 | 1.41 | 9.89E-04 | 2.99 |
| ENSG00000143226 | *FCGR2A* | 0 | 2.42 | 9.92E-04 | -1.64 |
| ENSG00000081041 | *CXCL2* | 0 | 3.01 | 9.96E-04 | 0.28 |
| ENSG00000134508 | *CABLES1* | 0 | 2.35 | 9.99E-04 | -1.96 |
| ENSG00000233608 | *TWIST2* | 0 | 1.30 | 1.01E-03 | 6.77 |
| ENSG00000139832 | *RAB20* | 0 | 1.67 | 1.02E-03 | 2.16 |
| ENSG00000166924 | *NYAP1* | 0 | 1.93 | 1.04E-03 | 0.82 |
| ENSG00000130822 | *PNCK* | 0 | 1.77 | 1.04E-03 | -0.98 |
| ENSG00000100767 | *PAPLN* | 0 | 2.37 | 1.05E-03 | 2.79 |
| ENSG00000259485 | *CTD-2147F2.1* | 0 | 2.95 | 1.05E-03 | -3.87 |
| ENSG00000138792 | *ENPEP* | 0 | 1.31 | 1.05E-03 | 0.14 |
| ENSG00000101224 | *CDC25B* | 0 | 1.08 | 1.06E-03 | 6.81 |
| ENSG00000164330 | *EBF1* | 0 | 1.18 | 1.10E-03 | 2.21 |
| ENSG00000184156 | *KCNQ3* | 0 | 1.45 | 1.12E-03 | 0.64 |
| ENSG00000072041 | *SLC6A15* | 0 | 4.24 | 1.13E-03 | 0.34 |
| ENSG00000133863 | *TEX15* | 0 | 3.12 | 1.14E-03 | -2.21 |
| ENSG00000160808 | *MYL3* | 0 | 2.12 | 1.15E-03 | -2.70 |
| ENSG00000235374 | *SSR4P1* | 0 | 1.37 | 1.15E-03 | 0.16 |
| ENSG00000148175 | *STOM* | 0 | 1.18 | 1.15E-03 | 8.19 |
| ENSG00000181885 | *CLDN7* | 0 | 1.35 | 1.17E-03 | 0.83 |
| ENSG00000133055 | *MYBPH* | 0 | 2.46 | 1.19E-03 | -0.28 |
| ENSG00000158270 | *COLEC12* | 0 | 2.96 | 1.21E-03 | 6.75 |
| ENSG00000129682 | *FGF13* | 0 | 1.77 | 1.22E-03 | -2.77 |
| ENSG00000235663 | *SAPCD1-AS1* | 0 | 2.13 | 1.23E-03 | -1.42 |
| ENSG00000197956 | *S100A6* | 0 | 1.07 | 1.23E-03 | 11.14 |
| ENSG00000168398 | *BDKRB2* | 0 | 1.27 | 1.23E-03 | 8.38 |
| ENSG00000235863 | *B3GALT4* | 0 | 1.02 | 1.24E-03 | 2.99 |
| ENSG00000138356 | *AOX1* | 0 | 2.23 | 1.25E-03 | 3.94 |
| ENSG00000236830 | *CBR3-AS1* | 0 | 1.15 | 1.27E-03 | 1.02 |
| ENSG00000117643 | *MAN1C1* | 0 | 1.98 | 1.27E-03 | 4.44 |
| ENSG00000137642 | *SORL1* | 0 | 1.68 | 1.27E-03 | 0.24 |
| ENSG00000035664 | *DAPK2* | 0 | 1.20 | 1.30E-03 | 2.81 |
| ENSG00000114378 | *HYAL1* | 0 | 1.90 | 1.30E-03 | 0.54 |
| ENSG00000138678 | *GPAT3* | 0 | 1.50 | 1.31E-03 | 2.40 |
| ENSG00000258376 | *RP4-647C14.2* | 0 | 1.50 | 1.36E-03 | 0.52 |
| ENSG00000243004 | *AC005062.2* | 0 | 2.53 | 1.37E-03 | -2.64 |
| ENSG00000122691 | *TWIST1* | 0 | 1.65 | 1.39E-03 | 5.15 |
| ENSG00000143867 | *OSR1* | 0 | 1.18 | 1.40E-03 | 3.56 |
| ENSG00000116711 | *PLA2G4A* | 0 | 1.21 | 1.41E-03 | 3.47 |
| ENSG00000196460 | *RFX8* | 0 | 2.23 | 1.42E-03 | 0.16 |
| ENSG00000248144 | *ADH1C* | 0 | 1.40 | 1.42E-03 | -0.59 |
| ENSG00000105643 | *ARRDC2* | 0 | 1.68 | 1.42E-03 | 3.86 |
| ENSG00000248161 | *RP11-499E18.1* | 0 | 1.32 | 1.42E-03 | 0.27 |
| ENSG00000175147 | *TMEM51-AS1* | 0 | 1.76 | 1.43E-03 | -0.53 |
| ENSG00000137767 | *SQRDL* | 0 | 1.16 | 1.44E-03 | 5.34 |
| ENSG00000107099 | *DOCK8* | 0 | 2.38 | 1.47E-03 | -2.15 |
| ENSG00000163520 | *FBLN2* | 0 | 1.32 | 1.48E-03 | 7.93 |
| ENSG00000142910 | *TINAGL1* | 0 | 1.37 | 1.48E-03 | 1.31 |
| ENSG00000085831 | *TTC39A* | 0 | 2.64 | 1.48E-03 | -2.10 |
| ENSG00000115107 | *STEAP3* | 0 | 1.03 | 1.48E-03 | 4.37 |
| ENSG00000189129 | *PLAC9* | 0 | 1.74 | 1.48E-03 | 2.66 |
| ENSG00000266010 | *GATA6-AS1* | 0 | 1.77 | 1.51E-03 | -1.33 |
| ENSG00000198829 | *SUCNR1* | 0 | 2.44 | 1.52E-03 | -0.54 |
| ENSG00000275178 | *RP11-4B16.3* | 0 | 2.20 | 1.56E-03 | -2.52 |
| ENSG00000124570 | *SERPINB6* | 0 | 1.08 | 1.56E-03 | 7.96 |
| ENSG00000121900 | *TMEM54* | 0 | 1.03 | 1.56E-03 | 2.37 |
| ENSG00000166546 | *BEAN1* | 0 | 1.67 | 1.58E-03 | 1.62 |
| ENSG00000080224 | *EPHA6* | 0 | 2.03 | 1.58E-03 | -0.47 |
| ENSG00000118503 | *TNFAIP3* | 0 | 1.44 | 1.59E-03 | 2.95 |
| ENSG00000034239 | *EFCAB1* | 0 | 1.72 | 1.59E-03 | 0.74 |
| ENSG00000136305 | *CIDEB* | 0 | 1.11 | 1.59E-03 | 2.12 |
| ENSG00000149451 | *ADAM33* | 0 | 1.17 | 1.59E-03 | 7.90 |
| ENSG00000250305 | *KIAA1456* | 0 | 1.52 | 1.59E-03 | 1.86 |
| ENSG00000135604 | *STX11* | 0 | 2.42 | 1.59E-03 | -0.78 |
| ENSG00000132561 | *MATN2* | 0 | 2.25 | 1.60E-03 | 3.36 |
| ENSG00000141574 | *SECTM1* | 0 | 2.25 | 1.60E-03 | 5.83 |
| ENSG00000166960 | *CCDC178* | 0 | 2.23 | 1.60E-03 | -2.23 |
| ENSG00000132554 | *RGS22* | 0 | 1.65 | 1.61E-03 | -0.76 |
| ENSG00000010030 | *ETV7* | 0 | 1.15 | 1.61E-03 | 0.86 |
| ENSG00000141448 | *GATA6* | 0 | 1.28 | 1.61E-03 | 2.11 |
| ENSG00000164136 | *IL15* | 0 | 1.10 | 1.61E-03 | 3.19 |
| ENSG00000146054 | *TRIM7* | 0 | 1.89 | 1.62E-03 | -0.87 |
| ENSG00000179921 | *GPBAR1* | 0 | 1.90 | 1.62E-03 | -2.33 |
| ENSG00000008118 | *CAMK1G* | 0 | 1.82 | 1.64E-03 | 3.52 |
| ENSG00000161149 | *TUBA3FP* | 0 | 1.11 | 1.64E-03 | 0.86 |
| ENSG00000146555 | *SDK1* | 0 | 1.49 | 1.65E-03 | 4.40 |
| ENSG00000198355 | *PIM3* | 0 | 1.11 | 1.66E-03 | 5.62 |
| ENSG00000105717 | *PBX4* | 0 | 1.67 | 1.66E-03 | -0.45 |
| ENSG00000112769 | *LAMA4* | 0 | 1.17 | 1.66E-03 | 8.24 |
| ENSG00000111728 | *ST8SIA1* | 0 | 1.26 | 1.68E-03 | 3.54 |
| ENSG00000226237 | *GAS1RR* | 0 | 1.29 | 1.68E-03 | 0.20 |
| ENSG00000137273 | *FOXF2* | 0 | 1.89 | 1.68E-03 | -3.27 |
| ENSG00000176438 | *SYNE3* | 0 | 1.01 | 1.70E-03 | 4.04 |
| ENSG00000132639 | *SNAP25* | 0 | 2.35 | 1.71E-03 | 3.46 |
| ENSG00000204261 | *PSMB8-AS1* | 0 | 1.11 | 1.73E-03 | 3.13 |
| ENSG00000160886 | *LY6K* | 0 | 1.68 | 1.75E-03 | 2.31 |
| ENSG00000187950 | *OVCH1* | 0 | 2.26 | 1.78E-03 | -2.58 |
| ENSG00000162881 | *OXER1* | 0 | 1.73 | 1.80E-03 | -2.25 |
| ENSG00000116016 | *EPAS1* | 0 | 1.56 | 1.80E-03 | 6.46 |
| ENSG00000073282 | *TP63* | 0 | 2.64 | 1.80E-03 | 0.04 |
| ENSG00000130052 | *STARD8* | 0 | 1.03 | 1.80E-03 | 2.86 |
| ENSG00000225361 | *PPP1R26-AS1* | 0 | 1.44 | 1.80E-03 | 0.03 |
| ENSG00000134802 | *SLC43A3* | 0 | 1.07 | 1.81E-03 | 3.59 |
| ENSG00000089199 | *CHGB* | 0 | 2.38 | 1.81E-03 | 0.49 |
| ENSG00000152270 | *PDE3B* | 0 | 2.03 | 1.83E-03 | -0.59 |
| ENSG00000089127 | *OAS1* | 0 | 1.24 | 1.83E-03 | 4.40 |
| ENSG00000203685 | *STUM* | 0 | 3.42 | 1.83E-03 | -1.89 |
| ENSG00000113532 | *ST8SIA4* | 0 | 2.11 | 1.85E-03 | 1.94 |
| ENSG00000112303 | *VNN2* | 0 | 4.79 | 1.90E-03 | -3.11 |
| ENSG00000140459 | *CYP11A1* | 0 | 1.94 | 1.91E-03 | -1.14 |
| ENSG00000178695 | *KCTD12* | 0 | 2.28 | 1.92E-03 | 6.09 |
| ENSG00000129595 | *EPB41L4A* | 0 | 1.92 | 1.96E-03 | -2.11 |
| ENSG00000135127 | *BICDL1* | 0 | 2.39 | 1.96E-03 | -2.16 |
| ENSG00000204228 | *HSD17B8* | 0 | 1.03 | 1.97E-03 | 1.68 |
| ENSG00000237523 | *LINC00857* | 0 | 1.12 | 1.97E-03 | 2.35 |
| ENSG00000221869 | *CEBPD* | 0 | 1.94 | 2.01E-03 | 5.10 |
| ENSG00000260244 | *RP11-588K22.2* | 0 | 2.13 | 2.04E-03 | 0.50 |
| ENSG00000197766 | *CFD* | 0 | 2.58 | 2.05E-03 | -0.04 |
| ENSG00000183807 | *FAM162B* | 0 | 1.32 | 2.09E-03 | 2.25 |
| ENSG00000189058 | *APOD* | 0 | 1.13 | 2.10E-03 | 7.09 |
| ENSG00000159403 | *C1R* | 0 | 1.34 | 2.14E-03 | 8.82 |
| ENSG00000186868 | *MAPT* | 0 | 2.30 | 2.18E-03 | 0.52 |
| ENSG00000224167 | *RP3-522D1.1* | 0 | 1.77 | 2.19E-03 | -2.75 |
| ENSG00000248290 | *TNXA* | 0 | 2.19 | 2.24E-03 | -4.71 |
| ENSG00000182866 | *LCK* | 0 | 3.13 | 2.26E-03 | -3.48 |
| ENSG00000169715 | *MT1E* | 0 | 1.01 | 2.26E-03 | 4.22 |
| ENSG00000075884 | *ARHGAP15* | 0 | 3.78 | 2.27E-03 | -4.35 |
| ENSG00000205403 | *CFI* | 0 | 1.37 | 2.30E-03 | 5.69 |
| ENSG00000255366 | *RP11-1134I14.8* | 0 | 2.86 | 2.30E-03 | -3.45 |
| ENSG00000166833 | *NAV2* | 0 | 1.18 | 2.34E-03 | 3.95 |
| ENSG00000047648 | *ARHGAP6* | 0 | 1.13 | 2.34E-03 | 4.04 |
| ENSG00000272341 | *RP1-151F17.2* | 0 | 1.15 | 2.37E-03 | 2.04 |
| ENSG00000134955 | *SLC37A2* | 0 | 1.15 | 2.37E-03 | 1.61 |
| ENSG00000104043 | *ATP8B4* | 0 | 1.98 | 2.37E-03 | 4.47 |
| ENSG00000206384 | *COL6A6* | 0 | 1.90 | 2.42E-03 | -0.38 |
| ENSG00000238150 | *AC008753.3* | 0 | 1.09 | 2.43E-03 | -0.21 |
| ENSG00000260552 | *RP11-49I11.1* | 0 | 1.28 | 2.43E-03 | -0.29 |
| ENSG00000164619 | *BMPER* | 0 | 1.57 | 2.44E-03 | 1.81 |
| ENSG00000223722 | *RP11-467L13.5* | 0 | 1.34 | 2.47E-03 | 0.37 |
| ENSG00000176406 | *RIMS2* | 0 | 1.71 | 2.54E-03 | -0.87 |
| ENSG00000174292 | *TNK1* | 0 | 1.68 | 2.61E-03 | -0.56 |
| ENSG00000119986 | *AVPI1* | 0 | 1.39 | 2.61E-03 | 3.54 |
| ENSG00000203943 | *SAMD13* | 0 | 1.25 | 2.62E-03 | 0.11 |
| ENSG00000164116 | *GUCY1A3* | 0 | 1.87 | 2.62E-03 | 1.52 |
| ENSG00000092929 | *UNC13D* | 0 | 1.65 | 2.64E-03 | 1.92 |
| ENSG00000125848 | *FLRT3* | 0 | 1.45 | 2.65E-03 | -0.07 |
| ENSG00000267272 | *LINC01140* | 0 | 2.24 | 2.65E-03 | -0.15 |
| ENSG00000197635 | *DPP4* | 0 | 1.30 | 2.68E-03 | 6.59 |
| ENSG00000270607 | *RP11-359E10.1* | 0 | 1.61 | 2.68E-03 | -0.85 |
| ENSG00000169297 | *NR0B1* | 0 | 4.37 | 2.71E-03 | -3.33 |
| ENSG00000075643 | *MOCOS* | 0 | 1.16 | 2.76E-03 | 0.80 |
| ENSG00000231064 | *MIR92B* | 0 | 1.19 | 2.79E-03 | 0.02 |
| ENSG00000204396 | *VWA7* | 0 | 1.39 | 2.84E-03 | -0.19 |
| ENSG00000272321 | *KB-1517D11.4* | 0 | 3.84 | 2.84E-03 | -2.52 |
| ENSG00000108001 | *EBF3* | 0 | 1.99 | 2.84E-03 | 0.52 |
| ENSG00000270069 | *MIR222HG* | 0 | 1.21 | 2.85E-03 | 3.61 |
| ENSG00000254139 | *CTD-2339F6.1* | 0 | 2.86 | 2.86E-03 | -4.89 |
| ENSG00000060656 | *PTPRU* | 0 | 1.26 | 2.88E-03 | 4.72 |
| ENSG00000162692 | *VCAM1* | 0 | 1.39 | 2.90E-03 | 1.71 |
| ENSG00000236453 | *AC003092.1* | 0 | 2.62 | 2.92E-03 | 0.11 |
| ENSG00000103723 | *AP3B2* | 0 | 2.08 | 2.93E-03 | -2.04 |
| ENSG00000188404 | *SELL* | 0 | 3.16 | 3.04E-03 | -5.06 |
| ENSG00000182397 | *DNM1P46* | 0 | 1.20 | 3.04E-03 | -0.94 |
| ENSG00000145681 | *HAPLN1* | 0 | 2.87 | 3.05E-03 | 0.57 |
| ENSG00000078579 | *FGF20* | 0 | 2.37 | 3.05E-03 | 0.14 |
| ENSG00000239332 | *LINC01119* | 0 | 1.35 | 3.07E-03 | 1.64 |
| ENSG00000136720 | *HS6ST1* | 0 | 1.11 | 3.07E-03 | 4.74 |
| ENSG00000006210 | *CX3CL1* | 0 | 2.08 | 3.11E-03 | 4.56 |
| ENSG00000166165 | *CKB* | 0 | 1.05 | 3.13E-03 | 2.17 |
| ENSG00000127241 | *MASP1* | 0 | 1.07 | 3.27E-03 | 8.05 |
| ENSG00000117594 | *HSD11B1* | 0 | 3.76 | 3.27E-03 | 1.23 |
| ENSG00000153898 | *MCOLN2* | 0 | 1.66 | 3.27E-03 | 1.72 |
| ENSG00000135298 | *ADGRB3* | 0 | 2.32 | 3.28E-03 | -1.51 |
| ENSG00000198400 | *NTRK1* | 0 | 2.14 | 3.28E-03 | -1.32 |
| ENSG00000132635 | *PCED1A* | 0 | 1.01 | 3.28E-03 | 6.39 |
| ENSG00000167157 | *PRRX2* | 0 | 1.92 | 3.28E-03 | 2.69 |
| ENSG00000179431 | *FJX1* | 0 | 1.16 | 3.29E-03 | 6.47 |
| ENSG00000165078 | *CPA6* | 0 | 2.56 | 3.29E-03 | -2.89 |
| ENSG00000155962 | *CLIC2* | 0 | 1.04 | 3.29E-03 | 4.38 |
| ENSG00000129467 | *ADCY4* | 0 | 1.76 | 3.34E-03 | 3.50 |
| ENSG00000173406 | *DAB1* | 0 | 2.55 | 3.36E-03 | -3.67 |
| ENSG00000027644 | *INSRR* | 0 | 1.29 | 3.36E-03 | -0.70 |
| ENSG00000128536 | *CDHR3* | 0 | 1.26 | 3.43E-03 | 2.18 |
| ENSG00000177453 | *NIM1K* | 0 | 1.25 | 3.44E-03 | 0.08 |
| ENSG00000168010 | *ATG16L2* | 0 | 1.06 | 3.44E-03 | 5.14 |
| ENSG00000156218 | *ADAMTSL3* | 0 | 1.74 | 3.44E-03 | -2.53 |
| ENSG00000089692 | *LAG3* | 0 | 1.89 | 3.46E-03 | 3.54 |
| ENSG00000228314 | *CYP4F29P* | 0 | 1.61 | 3.52E-03 | -4.04 |
| ENSG00000165188 | *RNF183* | 0 | 2.27 | 3.53E-03 | -3.86 |
| ENSG00000163815 | *CLEC3B* | 0 | 2.09 | 3.53E-03 | 1.45 |
| ENSG00000184232 | *OAF* | 0 | 1.10 | 3.72E-03 | 7.11 |
| ENSG00000157303 | *SUSD3* | 0 | 1.20 | 3.74E-03 | 0.91 |
| ENSG00000109339 | *MAPK10* | 0 | 1.14 | 3.79E-03 | 3.43 |
| ENSG00000203709 | *C1orf132* | 0 | 1.35 | 3.79E-03 | 1.56 |
| ENSG00000125730 | *C3* | 0 | 2.54 | 3.81E-03 | 1.59 |
| ENSG00000116396 | *KCNC4* | 0 | 1.04 | 3.86E-03 | 1.20 |
| ENSG00000188037 | *CLCN1* | 0 | 1.92 | 3.89E-03 | -3.03 |
| ENSG00000112319 | *EYA4* | 0 | 3.03 | 3.89E-03 | -3.55 |
| ENSG00000240694 | *PNMA2* | 0 | 1.07 | 3.91E-03 | -1.42 |
| ENSG00000167536 | *DHRS13* | 0 | 1.25 | 3.94E-03 | 1.62 |
| ENSG00000145283 | *SLC10A6* | 0 | 2.33 | 3.94E-03 | -4.55 |
| ENSG00000101017 | *CD40* | 0 | 1.23 | 3.95E-03 | 1.33 |
| ENSG00000197381 | *ADARB1* | 0 | 1.05 | 3.96E-03 | 3.03 |
| ENSG00000168497 | *SDPR* | 0 | 1.00 | 3.97E-03 | 4.59 |
| ENSG00000183873 | *SCN5A* | 0 | 1.63 | 4.00E-03 | 0.31 |
| ENSG00000258498 | *DIO3OS* | 0 | 1.84 | 4.02E-03 | -3.57 |
| ENSG00000251301 | *RP11-81H14.2* | 0 | 2.82 | 4.11E-03 | -3.75 |
| ENSG00000146678 | *IGFBP1* | 0 | 3.38 | 4.11E-03 | -3.17 |
| ENSG00000275212 | *RP3-446N13.5* | 0 | 2.21 | 4.13E-03 | -1.20 |
| ENSG00000134326 | *CMPK2* | 0 | 1.27 | 4.13E-03 | 2.05 |
| ENSG00000249684 | *RP11-423H2.3* | 0 | 1.81 | 4.20E-03 | -1.08 |
| ENSG00000219693 | *FGF7P8* | 0 | 2.47 | 4.20E-03 | -3.69 |
| ENSG00000183837 | *PNMA3* | 0 | 1.93 | 4.29E-03 | -3.46 |
| ENSG00000029534 | *ANK1* | 0 | 1.15 | 4.29E-03 | 2.05 |
| ENSG00000138821 | *SLC39A8* | 0 | 1.19 | 4.31E-03 | 3.74 |
| ENSG00000179855 | *GIPC3* | 0 | 1.48 | 4.33E-03 | 0.79 |
| ENSG00000167077 | *MEI1* | 0 | 1.21 | 4.40E-03 | 0.01 |
| ENSG00000010438 | *PRSS3* | 0 | 1.06 | 4.40E-03 | 2.68 |
| ENSG00000135047 | *CTSL* | 0 | 1.12 | 4.40E-03 | 8.98 |
| ENSG00000105711 | *SCN1B* | 0 | 1.12 | 4.42E-03 | 3.48 |
| ENSG00000109794 | *FAM149A* | 0 | 1.16 | 4.44E-03 | 0.16 |
| ENSG00000112149 | *CD83* | 0 | 1.04 | 4.46E-03 | 2.18 |
| ENSG00000177989 | *ODF3B* | 0 | 1.12 | 4.56E-03 | -0.37 |
| ENSG00000241684 | *ADAMTS9-AS2* | 0 | 1.38 | 4.63E-03 | -0.80 |
| ENSG00000145335 | *SNCA* | 0 | 1.98 | 4.64E-03 | 1.65 |
| ENSG00000105852 | *PON3* | 0 | 2.12 | 4.70E-03 | -3.02 |
| ENSG00000240207 | *RP11-379F4.4* | 0 | 1.27 | 4.71E-03 | -0.67 |
| ENSG00000204136 | *GGTA1P* | 0 | 2.00 | 4.71E-03 | -3.06 |
| ENSG00000275491 | *RP5-1009E24.8* | 0 | 2.03 | 4.73E-03 | -2.67 |
| ENSG00000162840 | *MT2P1* | 0 | 1.17 | 4.75E-03 | 4.55 |
| ENSG00000139835 | *GRTP1* | 0 | 1.43 | 4.83E-03 | -0.20 |
| ENSG00000226674 | *TEX41* | 0 | 3.11 | 4.88E-03 | -4.08 |
| ENSG00000132170 | *PPARG* | 0 | 1.40 | 4.89E-03 | -0.35 |
| ENSG00000223478 | *RP11-545E17.3* | 0 | 1.16 | 4.91E-03 | 0.86 |
| ENSG00000166278 | *C2* | 0 | 1.86 | 4.91E-03 | 1.98 |
| ENSG00000068078 | *FGFR3* | 0 | 1.13 | 4.94E-03 | 0.35 |
| ENSG00000079385 | *CEACAM1* | 0 | 3.36 | 4.97E-03 | 1.19 |
| ENSG00000115919 | *KYNU* | 0 | 3.65 | 4.97E-03 | 0.86 |
| ENSG00000132623 | *ANKEF1* | 0 | 1.08 | 4.99E-03 | 0.24 |
| ENSG00000184113 | *CLDN5* | 0 | 2.71 | 5.07E-03 | -4.91 |
| ENSG00000126803 | *HSPA2* | 0 | 1.03 | 5.07E-03 | 6.31 |
| ENSG00000237886 | *NALT1* | 0 | 1.04 | 5.09E-03 | 1.42 |
| ENSG00000155367 | *PPM1J* | 0 | 1.48 | 5.10E-03 | -0.88 |
| ENSG00000115594 | *IL1R1* | 0 | 2.50 | 5.11E-03 | 6.80 |
| ENSG00000215910 | *C1orf167* | 0 | 2.19 | 5.13E-03 | -2.10 |
| ENSG00000169247 | *SH3TC2* | 0 | 1.41 | 5.13E-03 | 1.92 |
| ENSG00000123095 | *BHLHE41* | 0 | 1.07 | 5.17E-03 | 2.06 |
| ENSG00000205221 | *VIT* | 0 | 2.25 | 5.17E-03 | -0.97 |
| ENSG00000068831 | *RASGRP2* | 0 | 1.48 | 5.22E-03 | 2.80 |
| ENSG00000141338 | *ABCA8* | 0 | 1.06 | 5.25E-03 | 3.50 |
| ENSG00000187260 | *WDR86* | 0 | 2.57 | 5.25E-03 | -0.22 |
| ENSG00000137496 | *IL18BP* | 0 | 1.32 | 5.26E-03 | 2.32 |
| ENSG00000242861 | *RP11-285F7.2* | 0 | 1.40 | 5.28E-03 | 1.57 |
| ENSG00000183742 | *MACC1* | 0 | 2.03 | 5.33E-03 | -3.88 |
| ENSG00000101188 | *NTSR1* | 0 | 2.43 | 5.42E-03 | -0.18 |
| ENSG00000230266 | *XXYLT1-AS2* | 0 | 1.67 | 5.43E-03 | 0.54 |
| ENSG00000196139 | *AKR1C3* | 0 | 1.24 | 5.43E-03 | 3.37 |
| ENSG00000128573 | *FOXP2* | 0 | 1.10 | 5.43E-03 | 1.87 |
| ENSG00000165646 | *SLC18A2* | 0 | 2.58 | 5.50E-03 | -4.14 |
| ENSG00000108387 | *SEPT4* | 0 | 1.78 | 5.50E-03 | 0.32 |
| ENSG00000198520 | *C1orf228* | 0 | 1.29 | 5.53E-03 | 0.65 |
| ENSG00000160318 | *CLDND2* | 0 | 1.24 | 5.58E-03 | -0.47 |
| ENSG00000164318 | *EGFLAM* | 0 | 1.77 | 5.59E-03 | 1.43 |
| ENSG00000164929 | *BAALC* | 0 | 2.05 | 5.65E-03 | -0.09 |
| ENSG00000133107 | *TRPC4* | 0 | 1.15 | 5.65E-03 | 3.11 |
| ENSG00000073737 | *DHRS9* | 0 | 3.82 | 5.72E-03 | -4.45 |
| ENSG00000270062 | *RP11-248J18.3* | 0 | 1.15 | 5.77E-03 | -0.49 |
| ENSG00000111181 | *SLC6A12* | 0 | 3.01 | 5.79E-03 | -3.66 |
| ENSG00000271133 | *CTA-293F17.1* | 0 | 1.13 | 5.82E-03 | -0.06 |
| ENSG00000147408 | *CSGALNACT1* | 0 | 1.74 | 5.82E-03 | 2.86 |
| ENSG00000123689 | *G0S2* | 0 | 2.59 | 5.83E-03 | 1.19 |
| ENSG00000228784 | *LINC00954* | 0 | 1.90 | 5.86E-03 | -0.47 |
| ENSG00000141738 | *GRB7* | 0 | 1.23 | 5.88E-03 | 0.32 |
| ENSG00000132199 | *ENOSF1* | 0 | 1.01 | 5.88E-03 | 5.05 |
| ENSG00000103888 | *CEMIP* | 0 | 2.09 | 5.94E-03 | 6.62 |
| ENSG00000167103 | *PIP5KL1* | 0 | 1.42 | 5.95E-03 | 0.91 |
| ENSG00000234695 | *AC002076.10* | 0 | 3.03 | 6.04E-03 | -4.84 |
| ENSG00000144681 | *STAC* | 0 | 1.46 | 6.05E-03 | -1.28 |
| ENSG00000025039 | *RRAGD* | 0 | 1.79 | 6.08E-03 | -0.77 |
| ENSG00000151572 | *ANO4* | 0 | 1.40 | 6.10E-03 | 3.83 |
| ENSG00000253958 | *CLDN23* | 0 | 1.16 | 6.11E-03 | 2.54 |
| ENSG00000273677 | *WT1-AS_2* | 0 | 2.44 | 6.17E-03 | -5.20 |
| ENSG00000163884 | *KLF15* | 0 | 1.14 | 6.18E-03 | -2.10 |
| ENSG00000262655 | *SPON1* | 0 | 1.67 | 6.18E-03 | 1.89 |
| ENSG00000073350 | *LLGL2* | 0 | 1.23 | 6.18E-03 | -0.06 |
| ENSG00000108950 | *FAM20A* | 0 | 2.25 | 6.21E-03 | 5.96 |
| ENSG00000106351 | *AGFG2* | 0 | 1.11 | 6.27E-03 | 3.91 |
| ENSG00000171992 | *SYNPO* | 0 | 1.22 | 6.28E-03 | 4.11 |
| ENSG00000227036 | *LINC00511* | 0 | 1.55 | 6.28E-03 | -0.10 |
| ENSG00000232973 | *CYP1B1-AS1* | 0 | 2.25 | 6.36E-03 | -2.95 |
| ENSG00000105371 | *ICAM4* | 0 | 1.22 | 6.46E-03 | 0.24 |
| ENSG00000132958 | *TPTE2* | 0 | 2.14 | 6.59E-03 | -2.79 |
| ENSG00000251209 | *LINC00923* | 0 | 1.44 | 6.68E-03 | -2.66 |
| ENSG00000173578 | *XCR1* | 0 | 3.15 | 6.70E-03 | -5.14 |
| ENSG00000119630 | *PGF* | 0 | 1.72 | 6.75E-03 | 1.10 |
| ENSG00000183570 | *PCBP3* | 0 | 1.34 | 6.76E-03 | 0.08 |
| ENSG00000197769 | *MAP1LC3C* | 0 | 1.50 | 6.81E-03 | -2.16 |
| ENSG00000271947 | *RP11-439M11.1* | 0 | 1.41 | 6.81E-03 | -1.93 |
| ENSG00000169122 | *FAM110B* | 0 | 1.02 | 6.90E-03 | 3.55 |
| ENSG00000105514 | *RAB3D* | 0 | 1.21 | 6.93E-03 | 3.98 |
| ENSG00000140284 | *SLC27A2* | 0 | 2.75 | 6.97E-03 | -3.34 |
| ENSG00000152580 | *IGSF10* | 0 | 1.31 | 7.02E-03 | 3.20 |
| ENSG00000280237 | *MIR4697HG* | 0 | 3.09 | 7.02E-03 | -5.23 |
| ENSG00000185215 | *TNFAIP2* | 0 | 1.09 | 7.04E-03 | 6.00 |
| ENSG00000274718 | *RP11-346C4.3* | 0 | 1.43 | 7.29E-03 | -0.54 |
| ENSG00000261076 | *RP11-179B15.6* | 0 | 1.95 | 7.30E-03 | -1.80 |
| ENSG00000145632 | *PLK2* | 0 | 1.00 | 7.36E-03 | 7.58 |
| ENSG00000146072 | *TNFRSF21* | 0 | 1.24 | 7.36E-03 | 8.42 |
| ENSG00000221887 | *HMSD* | 0 | 1.06 | 7.37E-03 | 1.31 |
| ENSG00000105963 | *ADAP1* | 0 | 1.37 | 7.43E-03 | -2.41 |
| ENSG00000100298 | *APOBEC3H* | 0 | 2.59 | 7.47E-03 | -1.79 |
| ENSG00000007933 | *FMO3* | 0 | 1.40 | 7.55E-03 | -0.86 |
| ENSG00000167995 | *BEST1* | 0 | 1.21 | 7.57E-03 | 1.68 |
| ENSG00000214093 | *RP11-247I13.3* | 0 | 1.00 | 7.61E-03 | -0.23 |
| ENSG00000170214 | *ADRA1B* | 0 | 1.41 | 7.62E-03 | -2.35 |
| ENSG00000163576 | *EFHB* | 0 | 1.06 | 7.65E-03 | -0.78 |
| ENSG00000079101 | *CLUL1* | 0 | 3.00 | 7.68E-03 | -4.63 |
| ENSG00000261672 | *RP11-475B2.1* | 0 | 1.23 | 7.76E-03 | -1.72 |
| ENSG00000229644 | *NAMPTP1* | 0 | 1.07 | 7.76E-03 | 5.11 |
| ENSG00000184060 | *ADAP2* | 0 | 1.12 | 7.83E-03 | 0.26 |
| ENSG00000128262 | *POM121L9P* | 0 | 2.52 | 7.92E-03 | -1.97 |
| ENSG00000153291 | *SLC25A27* | 0 | 1.10 | 7.94E-03 | 3.13 |
| ENSG00000137819 | *PAQR5* | 0 | 2.01 | 8.05E-03 | 1.15 |
| ENSG00000170579 | *DLGAP1* | 0 | 1.52 | 8.19E-03 | -1.01 |
| ENSG00000205517 | *RGL3* | 0 | 1.40 | 8.20E-03 | 0.93 |
| ENSG00000255007 | *CTD-2589M5.4* | 0 | 2.52 | 8.20E-03 | -4.86 |
| ENSG00000170458 | *CD14* | 0 | 1.74 | 8.31E-03 | 0.62 |
| ENSG00000277511 | *CTD-2095E4.5* | 0 | 1.24 | 8.37E-03 | -0.58 |
| ENSG00000213943 | *KRT18P17* | 0 | 1.67 | 8.39E-03 | -2.26 |
| ENSG00000108342 | *CSF3* | 0 | 3.70 | 8.44E-03 | -1.18 |
| ENSG00000261786 | *RP4-555D20.2* | 0 | 2.28 | 8.48E-03 | -3.74 |
| ENSG00000184160 | *ADRA2C* | 0 | 1.75 | 8.53E-03 | 6.22 |
| ENSG00000053524 | *MCF2L2* | 0 | 1.36 | 8.60E-03 | -0.95 |
| ENSG00000084674 | *APOB* | 0 | 2.28 | 8.64E-03 | -4.91 |
| ENSG00000237429 | *RP1-159A19.4* | 0 | 1.62 | 8.75E-03 | -2.56 |
| ENSG00000279778 | *RP11-60A14.1* | 0 | 1.49 | 8.78E-03 | -2.05 |
| ENSG00000260001 | *TGFBR3L* | 0 | 1.28 | 8.86E-03 | -0.61 |
| ENSG00000082074 | *FYB* | 0 | 2.94 | 8.90E-03 | -4.10 |
| ENSG00000185090 | *MANEAL* | 0 | 1.03 | 9.01E-03 | 0.13 |
| ENSG00000273183 | *RP11-5C23.2* | 0 | 1.40 | 9.06E-03 | -2.03 |
| ENSG00000134533 | *RERG* | 0 | 3.13 | 9.08E-03 | -5.05 |
| ENSG00000260302 | *RP11-973H7.1* | 0 | 1.66 | 9.18E-03 | -3.05 |
| ENSG00000151136 | *BTBD11* | 0 | 1.70 | 9.28E-03 | -3.55 |
| ENSG00000099998 | *GGT5* | 0 | 1.79 | 9.32E-03 | 3.58 |
| ENSG00000231671 | *LINC01307* | 0 | 3.89 | 9.39E-03 | -4.79 |
| ENSG00000257433 | *RP1-197B17.3* | 0 | 1.06 | 9.49E-03 | 0.26 |
| ENSG00000166596 | *CFAP52* | 0 | 1.69 | 9.54E-03 | -3.13 |
| ENSG00000161544 | *CYGB* | 0 | 1.41 | 9.54E-03 | 5.17 |
| ENSG00000108700 | *CCL8* | 0 | 3.87 | 9.61E-03 | -1.52 |
| ENSG00000167748 | *KLK1* | 0 | 2.29 | 9.61E-03 | -3.58 |
| ENSG00000172817 | *CYP7B1* | 0 | 1.33 | 9.64E-03 | 0.20 |
| ENSG00000104368 | *PLAT* | 0 | 1.43 | 9.64E-03 | 9.90 |
| ENSG00000019991 | *HGF* | 0 | 1.21 | 9.64E-03 | 7.86 |
| ENSG00000221866 | *PLXNA4* | 0 | 1.51 | 9.64E-03 | -4.42 |
| ENSG00000279673 | *RP11-185E8.2* | 0 | 1.55 | 9.66E-03 | -0.63 |
| ENSG00000123119 | *NECAB1* | 0 | 2.15 | 9.72E-03 | -4.25 |
| ENSG00000167780 | *SOAT2* | 0 | 1.71 | 9.83E-03 | -1.43 |
| ENSG00000282851 | *BISPR* | 0 | 1.02 | 9.83E-03 | -0.53 |
| ENSG00000134343 | *ANO3* | 0 | 1.22 | 9.89E-03 | -3.50 |
|  |  |  |  |  |  |

**Supplementary table 2**

**Genes significantly (FDR<0.01) differentially downregulated (Fold change >2) in control and A83-01-treated eMSCs compared to control**

| EnsemblGeneID | Gene | Fold change log_2_ | | FDR | Ave Expr |  |
| --- | --- | --- | --- | --- | --- | --- |
|  |  | **Control** | **A83-01** |  |  |  |
| ENSG00000087253 | *LPCAT2* | 0 | -2.34 | 2.21E-06 | 5.64 |  |
| ENSG00000158258 | *CLSTN2* | 0 | -3.05 | 5.68E-06 | 4.33 |  |
| ENSG00000120594 | *PLXDC2* | 0 | -2.03 | 5.68E-06 | 5.78 |  |
| ENSG00000082781 | *ITGB5* | 0 | -1.59 | 5.68E-06 | 8.80 |  |
| ENSG00000135048 | *TMEM2* | 0 | -1.88 | 5.68E-06 | 6.50 |  |
| ENSG00000170373 | *CST1* | 0 | -5.66 | 5.68E-06 | 3.95 |  |
| ENSG00000124225 | *PMEPA1* | 0 | -4.85 | 5.68E-06 | 2.71 |  |
| ENSG00000147041 | *SYTL5* | 0 | -3.30 | 5.68E-06 | 4.45 |  |
| ENSG00000076716 | *GPC4* | 0 | -2.52 | 6.68E-06 | 1.79 |  |
| ENSG00000129116 | *PALLD* | 0 | -2.71 | 6.68E-06 | 7.87 |  |
| ENSG00000139973 | *SYT16* | 0 | -4.45 | 6.68E-06 | 0.10 |  |
| ENSG00000110693 | *SOX6* | 0 | -2.45 | 6.68E-06 | 2.41 |  |
| ENSG00000128487 | *SPECC1* | 0 | -1.61 | 6.68E-06 | 5.38 |  |
| ENSG00000119938 | *PPP1R3C* | 0 | -1.90 | 6.68E-06 | 5.48 |  |
| ENSG00000157680 | *DGKI* | 0 | -2.52 | 6.68E-06 | 2.05 |  |
| ENSG00000072422 | *RHOBTB1* | 0 | -2.13 | 6.68E-06 | 5.20 |  |
| ENSG00000135333 | *EPHA7* | 0 | -3.63 | 8.58E-06 | 1.40 |  |
| ENSG00000182985 | *CADM1* | 0 | -3.49 | 9.26E-06 | 3.52 |  |
| ENSG00000143341 | *HMCN1* | 0 | -2.98 | 1.07E-05 | 0.85 |  |
| ENSG00000111110 | *PPM1H* | 0 | -2.35 | 1.25E-05 | 1.26 |  |
| ENSG00000159674 | *SPON2* | 0 | -3.79 | 1.50E-05 | 8.93 |  |
| ENSG00000141469 | *SLC14A1* | 0 | -3.07 | 1.65E-05 | 0.76 |  |
| ENSG00000197892 | *KIF13B* | 0 | -1.58 | 1.68E-05 | 4.08 |  |
| ENSG00000073910 | *FRY* | 0 | -2.45 | 1.73E-05 | 4.66 |  |
| ENSG00000179546 | *HTR1D* | 0 | -5.41 | 2.09E-05 | -0.59 |  |
| ENSG00000049540 | *ELN* | 0 | -5.00 | 2.16E-05 | 2.35 |  |
| ENSG00000134986 | *NREP* | 0 | -1.92 | 2.16E-05 | 5.87 |  |
| ENSG00000164251 | *F2RL1* | 0 | -2.44 | 2.24E-05 | 6.58 |  |
| ENSG00000150051 | *MKX* | 0 | -3.10 | 2.39E-05 | 1.53 |  |
| ENSG00000146038 | *DCDC2* | 0 | -2.53 | 2.39E-05 | 0.10 |  |
| ENSG00000101441 | *CST4* | 0 | -5.08 | 2.59E-05 | 0.00 |  |
| ENSG00000254726 | *MEX3A* | 0 | -2.97 | 2.59E-05 | 4.04 |  |
| ENSG00000198768 | *APCDD1L* | 0 | -2.22 | 2.59E-05 | -0.21 |  |
| ENSG00000100473 | *COCH* | 0 | -5.55 | 2.63E-05 | 3.33 |  |
| ENSG00000109265 | *KIAA1211* | 0 | -1.89 | 2.75E-05 | 3.68 |  |
| ENSG00000176595 | *KBTBD11* | 0 | -3.41 | 2.78E-05 | -0.63 |  |
| ENSG00000134243 | *SORT1* | 0 | -1.69 | 3.09E-05 | 4.75 |  |
| ENSG00000164099 | *PRSS12* | 0 | -1.79 | 3.09E-05 | 4.67 |  |
| ENSG00000166033 | *HTRA1* | 0 | -3.11 | 3.41E-05 | 6.87 |  |
| ENSG00000213190 | *MLLT11* | 0 | -1.47 | 3.41E-05 | 5.41 |  |
| ENSG00000106003 | *LFNG* | 0 | -2.31 | 3.41E-05 | 2.73 |  |
| ENSG00000102230 | *PCYT1B* | 0 | -3.08 | 3.43E-05 | -0.44 |  |
| ENSG00000166923 | *GREM1* | 0 | -2.13 | 3.73E-05 | 7.07 |  |
| ENSG00000236609 | *ZNF853* | 0 | -1.96 | 3.81E-05 | 0.45 |  |
| ENSG00000077274 | *CAPN6* | 0 | -2.00 | 4.62E-05 | 1.51 |  |
| ENSG00000121966 | *CXCR4* | 0 | -4.99 | 4.62E-05 | -0.79 |  |
| ENSG00000222032 | *AC112721.2* | 0 | -4.59 | 4.62E-05 | -1.80 |  |
| ENSG00000139971 | *C14orf37* | 0 | -1.82 | 4.62E-05 | 3.49 |  |
| ENSG00000117115 | *PADI2* | 0 | -5.17 | 4.62E-05 | -2.19 |  |
| ENSG00000095637 | *SORBS1* | 0 | -2.49 | 4.80E-05 | 1.66 |  |
| ENSG00000231298 | *LINC00704* | 0 | -4.88 | 4.83E-05 | -1.71 |  |
| ENSG00000114805 | *PLCH1* | 0 | -3.63 | 5.03E-05 | 3.48 |  |
| ENSG00000089472 | *HEPH* | 0 | -1.16 | 5.08E-05 | 4.36 |  |
| ENSG00000165434 | *PGM2L1* | 0 | -2.17 | 5.21E-05 | 3.93 |  |
| ENSG00000152952 | *PLOD2* | 0 | -1.67 | 5.28E-05 | 7.55 |  |
| ENSG00000165197 | *VEGFD* | 0 | -2.77 | 5.31E-05 | -0.65 |  |
| ENSG00000135362 | *PRR5L* | 0 | -3.70 | 6.38E-05 | 2.22 |  |
| ENSG00000107643 | *MAPK8* | 0 | -1.07 | 6.43E-05 | 5.66 |  |
| ENSG00000143494 | *VASH2* | 0 | -3.91 | 6.89E-05 | 1.20 |  |
| ENSG00000119714 | *GPR68* | 0 | -2.05 | 6.99E-05 | -0.01 |  |
| ENSG00000162267 | *ITIH3* | 0 | -3.00 | 7.01E-05 | 1.00 |  |
| ENSG00000222022 | *AC112721.1* | 0 | -5.37 | 7.04E-05 | -2.16 |  |
| ENSG00000166670 | *MMP10* | 0 | -4.80 | 7.04E-05 | 5.73 |  |
| ENSG00000099953 | *MMP11* | 0 | -3.27 | 7.04E-05 | 8.52 |  |
| ENSG00000198945 | *L3MBTL3* | 0 | -1.31 | 7.04E-05 | 4.63 |  |
| ENSG00000167244 | *IGF2* | 0 | -2.18 | 7.29E-05 | 4.92 |  |
| ENSG00000060718 | *COL11A1* | 0 | -4.02 | 7.33E-05 | 1.91 |  |
| ENSG00000116679 | *IVNS1ABP* | 0 | -1.15 | 7.33E-05 | 5.93 |  |
| ENSG00000145536 | *ADAMTS16* | 0 | -3.51 | 7.34E-05 | 0.28 |  |
| ENSG00000164796 | *CSMD3* | 0 | -3.64 | 7.34E-05 | 0.02 |  |
| ENSG00000092969 | *TGFB2* | 0 | -2.30 | 8.00E-05 | 2.77 |  |
| ENSG00000159251 | *ACTC1* | 0 | -4.50 | 8.00E-05 | 0.04 |  |
| ENSG00000197614 | *MFAP5* | 0 | -5.38 | 8.08E-05 | -0.99 |  |
| ENSG00000254645 | *RP11-396O20.2* | 0 | -5.66 | 8.15E-05 | -3.10 |  |
| ENSG00000100523 | *DDHD1* | 0 | -1.16 | 8.15E-05 | 5.58 |  |
| ENSG00000169047 | *IRS1* | 0 | -1.65 | 8.15E-05 | 6.01 |  |
| ENSG00000138623 | *SEMA7A* | 0 | -2.49 | 8.15E-05 | 4.97 |  |
| ENSG00000078401 | *EDN1* | 0 | -2.84 | 8.15E-05 | 0.05 |  |
| ENSG00000133460 | *SLC2A11* | 0 | -1.41 | 8.15E-05 | 4.05 |  |
| ENSG00000122641 | *INHBA* | 0 | -2.39 | 8.30E-05 | 5.24 |  |
| ENSG00000170044 | *ZPLD1* | 0 | -6.21 | 8.30E-05 | -1.21 |  |
| ENSG00000173894 | *CBX2* | 0 | -1.50 | 8.30E-05 | 2.25 |  |
| ENSG00000168268 | *NT5DC2* | 0 | -1.13 | 8.30E-05 | 6.54 |  |
| ENSG00000138435 | *CHRNA1* | 0 | -6.33 | 8.30E-05 | -1.97 |  |
| ENSG00000172331 | *BPGM* | 0 | -1.19 | 8.44E-05 | 5.20 |  |
| ENSG00000162944 | *RFTN2* | 0 | -1.93 | 9.48E-05 | 3.91 |  |
| ENSG00000259977 | *AL121578.2* | 0 | -3.52 | 9.83E-05 | -2.66 |  |
| ENSG00000146197 | *SCUBE3* | 0 | -1.77 | 9.86E-05 | 3.48 |  |
| ENSG00000110900 | *TSPAN11* | 0 | -3.70 | 1.00E-04 | 1.46 |  |
| ENSG00000131018 | *SYNE1* | 0 | -1.14 | 1.01E-04 | 5.94 |  |
| ENSG00000103175 | *WFDC1* | 0 | -3.39 | 1.01E-04 | 2.39 |  |
| ENSG00000131620 | *ANO1* | 0 | -2.75 | 1.05E-04 | -1.88 |  |
| ENSG00000118257 | *NRP2* | 0 | -1.48 | 1.05E-04 | 5.82 |  |
| ENSG00000120156 | *TEK* | 0 | -2.31 | 1.14E-04 | 3.41 |  |
| ENSG00000164823 | *OSGIN2* | 0 | -2.68 | 1.14E-04 | 3.67 |  |
| ENSG00000136378 | *ADAMTS7* | 0 | -1.65 | 1.14E-04 | 5.04 |  |
| ENSG00000136052 | *SLC41A2* | 0 | -1.32 | 1.14E-04 | 2.43 |  |
| ENSG00000033867 | *SLC4A7* | 0 | -1.01 | 1.19E-04 | 4.95 |  |
| ENSG00000250303 | *RP11-356J5.12* | 0 | -2.02 | 1.21E-04 | 2.64 |  |
| ENSG00000074590 | *NUAK1* | 0 | -2.22 | 1.26E-04 | 5.32 |  |
| ENSG00000162849 | *KIF26B* | 0 | -2.21 | 1.26E-04 | 5.23 |  |
| ENSG00000145244 | *CORIN* | 0 | -3.11 | 1.26E-04 | 3.03 |  |
| ENSG00000186369 | *LINC00643* | 0 | -4.59 | 1.27E-04 | -1.09 |  |
| ENSG00000127418 | *FGFRL1* | 0 | -1.07 | 1.27E-04 | 4.21 |  |
| ENSG00000175084 | *DES* | 0 | -1.88 | 1.27E-04 | 0.87 |  |
| ENSG00000187605 | *TET3* | 0 | -1.10 | 1.30E-04 | 4.16 |  |
| ENSG00000128606 | *LRRC17* | 0 | -2.85 | 1.32E-04 | 6.47 |  |
| ENSG00000006453 | *BAIAP2L1* | 0 | -1.83 | 1.34E-04 | 2.56 |  |
| ENSG00000111341 | *MGP* | 0 | -4.10 | 1.34E-04 | 3.42 |  |
| ENSG00000111799 | *COL12A1* | 0 | -1.56 | 1.34E-04 | 11.49 |  |
| ENSG00000114251 | *WNT5A* | 0 | -1.96 | 1.35E-04 | 9.40 |  |
| ENSG00000099889 | *ARVCF* | 0 | -1.63 | 1.35E-04 | 3.27 |  |
| ENSG00000137942 | *FNBP1L* | 0 | -1.27 | 1.35E-04 | 4.31 |  |
| ENSG00000136546 | *SCN7A* | 0 | -2.88 | 1.37E-04 | -1.12 |  |
| ENSG00000204767 | *FAM196B* | 0 | -2.54 | 1.37E-04 | 1.91 |  |
| ENSG00000145431 | *PDGFC* | 0 | -1.83 | 1.37E-04 | 6.29 |  |
| ENSG00000103187 | *COTL1* | 0 | -1.55 | 1.42E-04 | 7.21 |  |
| ENSG00000158186 | *MRAS* | 0 | -1.24 | 1.42E-04 | 5.55 |  |
| ENSG00000170624 | *SGCD* | 0 | -2.13 | 1.44E-04 | 3.07 |  |
| ENSG00000147180 | *ZNF711* | 0 | -1.27 | 1.44E-04 | 3.25 |  |
| ENSG00000170369 | *CST2* | 0 | -4.25 | 1.45E-04 | 1.04 |  |
| ENSG00000154556 | *SORBS2* | 0 | -3.86 | 1.46E-04 | -2.18 |  |
| ENSG00000115414 | *FN1* | 0 | -2.38 | 1.46E-04 | 12.03 |  |
| ENSG00000025423 | *HSD17B6* | 0 | -2.06 | 1.57E-04 | 1.48 |  |
| ENSG00000165929 | *TC2N* | 0 | -2.14 | 1.59E-04 | -1.70 |  |
| ENSG00000136603 | *SKIL* | 0 | -1.64 | 1.60E-04 | 5.17 |  |
| ENSG00000259370 | *RP11-1069G10.1* | 0 | -3.74 | 1.62E-04 | -2.74 |  |
| ENSG00000001617 | *SEMA3F* | 0 | -2.06 | 1.64E-04 | 0.67 |  |
| ENSG00000123243 | *ITIH5* | 0 | -2.96 | 1.68E-04 | -1.06 |  |
| ENSG00000162551 | *ALPL* | 0 | -4.61 | 1.71E-04 | -0.05 |  |
| ENSG00000128805 | *ARHGAP22* | 0 | -1.65 | 1.71E-04 | 4.18 |  |
| ENSG00000021645 | *NRXN3* | 0 | -3.09 | 1.75E-04 | 2.14 |  |
| ENSG00000088387 | *DOCK9* | 0 | -1.25 | 1.84E-04 | 4.13 |  |
| ENSG00000223764 | *RP11-54O7.3* | 0 | -2.72 | 1.85E-04 | -0.15 |  |
| ENSG00000272398 | *CD24* | 0 | -3.87 | 1.88E-04 | -1.90 |  |
| ENSG00000166707 | *ZCCHC18* | 0 | -1.92 | 1.89E-04 | 0.51 |  |
| ENSG00000255690 | *TRIL* | 0 | -2.12 | 1.92E-04 | 3.12 |  |
| ENSG00000138795 | *LEF1* | 0 | -2.10 | 1.96E-04 | 1.91 |  |
| ENSG00000118707 | *TGIF2* | 0 | -1.04 | 2.04E-04 | 3.40 |  |
| ENSG00000164300 | *SERINC5* | 0 | -1.28 | 2.06E-04 | 4.48 |  |
| ENSG00000178585 | *CTNNBIP1* | 0 | -1.01 | 2.10E-04 | 4.64 |  |
| ENSG00000100368 | *CSF2RB* | 0 | -2.99 | 2.21E-04 | 0.03 |  |
| ENSG00000151150 | *ANK3* | 0 | -2.24 | 2.35E-04 | 0.67 |  |
| ENSG00000197312 | *DDI2* | 0 | -1.26 | 2.35E-04 | 3.88 |  |
| ENSG00000182054 | *IDH2* | 0 | -1.07 | 2.35E-04 | 5.78 |  |
| ENSG00000101825 | *MXRA5* | 0 | -1.52 | 2.42E-04 | 7.55 |  |
| ENSG00000130508 | *PXDN* | 0 | -1.15 | 2.54E-04 | 9.99 |  |
| ENSG00000233098 | *CCDC144NL-AS1* | 0 | -2.96 | 2.56E-04 | 0.30 |  |
| ENSG00000260604 | *RP1-140K8.5* | 0 | -2.98 | 2.61E-04 | -1.08 |  |
| ENSG00000135074 | *ADAM19* | 0 | -2.07 | 2.68E-04 | 6.79 |  |
| ENSG00000115468 | *EFHD1* | 0 | -3.60 | 2.72E-04 | 2.08 |  |
| ENSG00000104081 | *BMF* | 0 | -2.38 | 2.72E-04 | 4.38 |  |
| ENSG00000116329 | *OPRD1* | 0 | -4.42 | 2.72E-04 | -3.48 |  |
| ENSG00000147036 | *LANCL3* | 0 | -1.25 | 2.72E-04 | 1.02 |  |
| ENSG00000110660 | *SLC35F2* | 0 | -1.42 | 2.72E-04 | 3.24 |  |
| ENSG00000159023 | *EPB41* | 0 | -1.21 | 2.90E-04 | 0.61 |  |
| ENSG00000168280 | *KIF5C* | 0 | -2.52 | 2.90E-04 | -1.94 |  |
| ENSG00000091831 | *ESR1* | 0 | -1.49 | 2.93E-04 | 4.26 |  |
| ENSG00000198018 | *ENTPD7* | 0 | -1.33 | 2.93E-04 | 3.67 |  |
| ENSG00000214944 | *ARHGEF28* | 0 | -1.33 | 2.96E-04 | 4.44 |  |
| ENSG00000135083 | *CCNJL* | 0 | -1.14 | 2.96E-04 | 2.05 |  |
| ENSG00000112559 | *MDFI* | 0 | -2.82 | 2.99E-04 | -2.44 |  |
| ENSG00000183729 | *NPBWR1* | 0 | -4.04 | 3.00E-04 | -3.56 |  |
| ENSG00000166341 | *DCHS1* | 0 | -1.22 | 3.01E-04 | 8.06 |  |
| ENSG00000085741 | *WNT11* | 0 | -4.48 | 3.01E-04 | 0.96 |  |
| ENSG00000144749 | *LRIG1* | 0 | -1.92 | 3.01E-04 | 5.55 |  |
| ENSG00000073060 | *SCARB1* | 0 | -1.08 | 3.01E-04 | 5.65 |  |
| ENSG00000106819 | *ASPN* | 0 | -2.49 | 3.33E-04 | 3.48 |  |
| ENSG00000246430 | *LINC00968* | 0 | -3.66 | 3.33E-04 | -2.67 |  |
| ENSG00000188707 | *ZBED6CL* | 0 | -1.23 | 3.33E-04 | 1.85 |  |
| ENSG00000281655 | *RP11-817J15.3* | 0 | -4.78 | 3.33E-04 | -3.24 |  |
| ENSG00000142623 | *PADI1* | 0 | -2.88 | 3.33E-04 | -1.13 |  |
| ENSG00000117009 | *KMO* | 0 | -2.49 | 3.33E-04 | 0.13 |  |
| ENSG00000168675 | *LDLRAD4* | 0 | -2.81 | 3.36E-04 | 1.08 |  |
| ENSG00000167178 | *ISLR2* | 0 | -1.21 | 3.36E-04 | 3.24 |  |
| ENSG00000172548 | *NIPAL4* | 0 | -2.87 | 3.38E-04 | -1.80 |  |
| ENSG00000188133 | *TMEM215* | 0 | -5.08 | 3.40E-04 | -4.03 |  |
| ENSG00000111087 | *GLI1* | 0 | -2.08 | 3.41E-04 | 1.99 |  |
| ENSG00000163827 | *LRRC2* | 0 | -2.04 | 3.42E-04 | 0.90 |  |
| ENSG00000138316 | *ADAMTS14* | 0 | -1.45 | 3.50E-04 | 4.25 |  |
| ENSG00000155760 | *FZD7* | 0 | -1.41 | 3.52E-04 | 4.42 |  |
| ENSG00000100592 | *DAAM1* | 0 | -1.23 | 3.52E-04 | 3.36 |  |
| ENSG00000096696 | *DSP* | 0 | -2.59 | 3.54E-04 | 4.91 |  |
| ENSG00000130787 | *HIP1R* | 0 | -1.33 | 3.56E-04 | 3.91 |  |
| ENSG00000101935 | *AMMECR1* | 0 | -1.08 | 3.57E-04 | 3.97 |  |
| ENSG00000153982 | *GDPD1* | 0 | -1.41 | 3.58E-04 | 0.98 |  |
| ENSG00000148848 | *ADAM12* | 0 | -2.02 | 3.58E-04 | 6.84 |  |
| ENSG00000230838 | *LINC01614* | 0 | -4.59 | 3.60E-04 | -2.62 |  |
| ENSG00000218336 | *TENM3* | 0 | -1.32 | 3.61E-04 | 2.20 |  |
| ENSG00000087245 | *MMP2* | 0 | -1.75 | 3.61E-04 | 11.21 |  |
| ENSG00000198739 | *LRRTM3* | 0 | -3.30 | 3.61E-04 | -2.01 |  |
| ENSG00000280178 | *AP000349.2* | 0 | -4.17 | 3.61E-04 | -4.07 |  |
| ENSG00000137166 | *FOXP4* | 0 | -1.33 | 3.61E-04 | 4.36 |  |
| ENSG00000152377 | *SPOCK1* | 0 | -1.28 | 3.65E-04 | 8.14 |  |
| ENSG00000065923 | *SLC9A7* | 0 | -1.13 | 3.72E-04 | 4.52 |  |
| ENSG00000103034 | *NDRG4* | 0 | -1.46 | 3.89E-04 | 3.96 |  |
| ENSG00000164638 | *SLC29A4* | 0 | -1.59 | 3.89E-04 | 2.47 |  |
| ENSG00000226087 | *AC106869.2* | 0 | -4.60 | 3.91E-04 | -3.36 |  |
| ENSG00000172318 | *B3GALT1* | 0 | -2.71 | 4.07E-04 | -0.32 |  |
| ENSG00000149428 | *HYOU1* | 0 | -1.14 | 4.13E-04 | 7.12 |  |
| ENSG00000175183 | *CSRP2* | 0 | -1.74 | 4.15E-04 | 4.68 |  |
| ENSG00000146950 | *SHROOM2* | 0 | -1.86 | 4.22E-04 | 3.81 |  |
| ENSG00000211448 | *DIO2* | 0 | -2.47 | 4.26E-04 | 4.61 |  |
| ENSG00000133874 | *RNF122* | 0 | -1.30 | 4.29E-04 | 2.31 |  |
| ENSG00000198780 | *FAM169A* | 0 | -1.40 | 4.33E-04 | 0.96 |  |
| ENSG00000151892 | *GFRA1* | 0 | -2.11 | 4.34E-04 | 1.48 |  |
| ENSG00000162493 | *PDPN* | 0 | -1.41 | 4.34E-04 | 6.20 |  |
| ENSG00000077063 | *CTTNBP2* | 0 | -2.06 | 4.47E-04 | 0.62 |  |
| ENSG00000112796 | *ENPP5* | 0 | -1.80 | 4.50E-04 | -0.91 |  |
| ENSG00000113209 | *PCDHB5* | 0 | -1.13 | 4.53E-04 | 1.34 |  |
| ENSG00000224020 | *MIR181A2HG* | 0 | -1.93 | 4.55E-04 | 0.01 |  |
| ENSG00000166123 | *GPT2* | 0 | -1.70 | 4.59E-04 | 2.95 |  |
| ENSG00000168685 | *IL7R* | 0 | -1.50 | 4.72E-04 | 5.04 |  |
| ENSG00000124766 | *SOX4* | 0 | -2.40 | 4.78E-04 | 7.51 |  |
| ENSG00000108821 | *COL1A1* | 0 | -1.39 | 4.83E-04 | 14.07 |  |
| ENSG00000110841 | *PPFIBP1* | 0 | -1.06 | 4.84E-04 | 5.20 |  |
| ENSG00000138311 | *ZNF365* | 0 | -2.01 | 4.84E-04 | 2.16 |  |
| ENSG00000246273 | *SBF2-AS1* | 0 | -1.31 | 4.89E-04 | 5.95 |  |
| ENSG00000140092 | *FBLN5* | 0 | -1.02 | 4.89E-04 | 8.28 |  |
| ENSG00000197584 | *KCNMB2* | 0 | -3.41 | 4.89E-04 | -1.03 |  |
| ENSG00000213949 | *ITGA1* | 0 | -1.92 | 4.93E-04 | 7.09 |  |
| ENSG00000197361 | *FBXL22* | 0 | -2.23 | 4.96E-04 | -0.47 |  |
| ENSG00000176371 | *ZSCAN2* | 0 | -1.09 | 5.11E-04 | 3.47 |  |
| ENSG00000183688 | *RFLNB* | 0 | -1.49 | 5.11E-04 | 5.79 |  |
| ENSG00000108176 | *DNAJC12* | 0 | -1.51 | 5.11E-04 | 0.62 |  |
| ENSG00000114631 | *PODXL2* | 0 | -1.43 | 5.18E-04 | 1.38 |  |
| ENSG00000172260 | *NEGR1* | 0 | -1.96 | 5.18E-04 | 3.60 |  |
| ENSG00000198523 | *PLN* | 0 | -4.23 | 5.18E-04 | -1.78 |  |
| ENSG00000174343 | *CHRNA9* | 0 | -4.04 | 5.18E-04 | -3.71 |  |
| ENSG00000156466 | *GDF6* | 0 | -3.60 | 5.28E-04 | -2.72 |  |
| ENSG00000137571 | *SLCO5A1* | 0 | -2.46 | 5.28E-04 | -1.68 |  |
| ENSG00000213420 | *GPC2* | 0 | -2.02 | 5.30E-04 | 1.99 |  |
| ENSG00000154380 | *ENAH* | 0 | -1.50 | 5.43E-04 | 6.11 |  |
| ENSG00000176658 | *MYO1D* | 0 | -1.19 | 5.43E-04 | 6.43 |  |
| ENSG00000277778 | *PGM5P2* | 0 | -1.90 | 5.45E-04 | -1.50 |  |
| ENSG00000065600 | *TMEM206* | 0 | -1.26 | 5.49E-04 | 3.60 |  |
| ENSG00000123612 | *ACVR1C* | 0 | -2.20 | 5.55E-04 | -0.30 |  |
| ENSG00000213626 | *LBH* | 0 | -1.61 | 5.56E-04 | 6.58 |  |
| ENSG00000263745 | *RP11-161I6.2* | 0 | -3.88 | 5.56E-04 | -4.50 |  |
| ENSG00000111859 | *NEDD9* | 0 | -3.02 | 5.59E-04 | 2.91 |  |
| ENSG00000115232 | *ITGA4* | 0 | -1.62 | 5.68E-04 | 6.32 |  |
| ENSG00000147044 | *CASK* | 0 | -1.18 | 5.70E-04 | 5.98 |  |
| ENSG00000208024 | *MIR199A2* | 0 | -1.88 | 5.73E-04 | -1.98 |  |
| ENSG00000165617 | *DACT1* | 0 | -1.41 | 5.73E-04 | 2.34 |  |
| ENSG00000119681 | *LTBP2* | 0 | -1.05 | 5.74E-04 | 10.21 |  |
| ENSG00000102996 | *MMP15* | 0 | -1.84 | 5.77E-04 | 1.52 |  |
| ENSG00000064692 | *SNCAIP* | 0 | -1.95 | 5.83E-04 | 5.41 |  |
| ENSG00000138771 | *SHROOM3* | 0 | -1.54 | 5.91E-04 | 4.77 |  |
| ENSG00000072133 | *RPS6KA6* | 0 | -1.48 | 5.91E-04 | 3.32 |  |
| ENSG00000162738 | *VANGL2* | 0 | -1.97 | 5.92E-04 | -0.56 |  |
| ENSG00000256812 | *CAPNS2* | 0 | -3.98 | 6.04E-04 | -4.41 |  |
| ENSG00000183876 | *ARSI* | 0 | -1.54 | 6.23E-04 | 1.85 |  |
| ENSG00000278709 | *NKILA* | 0 | -3.00 | 6.38E-04 | -2.46 |  |
| ENSG00000187498 | *COL4A1* | 0 | -1.82 | 6.54E-04 | 10.89 |  |
| ENSG00000105989 | *WNT2* | 0 | -2.32 | 6.59E-04 | 5.74 |  |
| ENSG00000259834 | *RP11-284N8.3* | 0 | -3.27 | 6.60E-04 | -1.98 |  |
| ENSG00000167552 | *TUBA1A* | 0 | -1.16 | 6.60E-04 | 8.21 |  |
| ENSG00000149380 | *P4HA3* | 0 | -1.75 | 6.64E-04 | 2.19 |  |
| ENSG00000178033 | *FAM26E* | 0 | -1.87 | 6.65E-04 | 2.63 |  |
| ENSG00000106070 | *GRB10* | 0 | -1.04 | 6.68E-04 | 4.61 |  |
| ENSG00000163328 | *GPR155* | 0 | -1.09 | 6.76E-04 | 2.10 |  |
| ENSG00000180263 | *FGD6* | 0 | -1.01 | 6.76E-04 | 3.16 |  |
| ENSG00000105664 | *COMP* | 0 | -5.57 | 6.77E-04 | -0.94 |  |
| ENSG00000150054 | *MPP7* | 0 | -2.55 | 6.99E-04 | -1.63 |  |
| ENSG00000127399 | *LRRC61* | 0 | -1.09 | 6.99E-04 | 2.63 |  |
| ENSG00000155926 | *SLA* | 0 | -1.63 | 7.18E-04 | 0.87 |  |
| ENSG00000164185 | *ZNF474* | 0 | -1.90 | 7.19E-04 | -1.18 |  |
| ENSG00000041982 | *TNC* | 0 | -2.44 | 7.23E-04 | 9.13 |  |
| ENSG00000157445 | *CACNA2D3* | 0 | -1.92 | 7.35E-04 | 2.15 |  |
| ENSG00000173391 | *OLR1* | 0 | -1.12 | 7.37E-04 | 3.17 |  |
| ENSG00000105976 | *MET* | 0 | -1.94 | 7.42E-04 | 3.46 |  |
| ENSG00000130176 | *CNN1* | 0 | -2.71 | 7.54E-04 | 4.30 |  |
| ENSG00000038427 | *VCAN* | 0 | -1.87 | 7.71E-04 | 8.17 |  |
| ENSG00000164588 | *HCN1* | 0 | -4.55 | 7.71E-04 | -3.92 |  |
| ENSG00000134871 | *COL4A2* | 0 | -1.32 | 7.96E-04 | 11.53 |  |
| ENSG00000155011 | *DKK2* | 0 | -3.26 | 8.10E-04 | -0.48 |  |
| ENSG00000144218 | *AFF3* | 0 | -1.93 | 8.11E-04 | 5.36 |  |
| ENSG00000120324 | *PCDHB10* | 0 | -1.17 | 8.12E-04 | 0.71 |  |
| ENSG00000206190 | *ATP10A* | 0 | -2.73 | 8.12E-04 | -0.27 |  |
| ENSG00000170571 | *EMB* | 0 | -1.48 | 8.14E-04 | -0.01 |  |
| ENSG00000158683 | *PKD1L1* | 0 | -2.71 | 8.14E-04 | -0.19 |  |
| ENSG00000251405 | *CTB-109A12.1* | 0 | -3.19 | 8.15E-04 | -2.31 |  |
| ENSG00000117525 | *F3* | 0 | -1.41 | 8.16E-04 | 6.74 |  |
| ENSG00000160145 | *KALRN* | 0 | -1.12 | 8.30E-04 | 2.75 |  |
| ENSG00000188322 | *SBK1* | 0 | -2.34 | 8.30E-04 | -1.55 |  |
| ENSG00000196159 | *FAT4* | 0 | -1.60 | 8.30E-04 | 5.71 |  |
| ENSG00000149294 | *NCAM1* | 0 | -2.31 | 8.41E-04 | 2.49 |  |
| ENSG00000228221 | *LINC00578* | 0 | -1.84 | 8.42E-04 | -2.96 |  |
| ENSG00000162415 | *ZSWIM5* | 0 | -1.13 | 8.54E-04 | 3.98 |  |
| ENSG00000179598 | *PLD6* | 0 | -1.01 | 8.56E-04 | 2.42 |  |
| ENSG00000106537 | *TSPAN13* | 0 | -1.75 | 8.69E-04 | 3.19 |  |
| ENSG00000139055 | *ERP27* | 0 | -2.98 | 8.73E-04 | -3.90 |  |
| ENSG00000121898 | *CPXM2* | 0 | -1.71 | 8.73E-04 | 0.85 |  |
| ENSG00000185972 | *CCIN* | 0 | -3.46 | 8.85E-04 | -2.10 |  |
| ENSG00000123213 | *NLN* | 0 | -1.04 | 9.15E-04 | 4.90 |  |
| ENSG00000118194 | *TNNT2* | 0 | -2.33 | 9.15E-04 | -2.99 |  |
| ENSG00000171033 | *PKIA* | 0 | -1.64 | 9.21E-04 | 1.66 |  |
| ENSG00000137801 | *THBS1* | 0 | -2.45 | 9.42E-04 | 10.16 |  |
| ENSG00000143195 | *ILDR2* | 0 | -2.08 | 9.42E-04 | -2.93 |  |
| ENSG00000136542 | *GALNT5* | 0 | -1.12 | 9.43E-04 | 5.48 |  |
| ENSG00000142149 | *HUNK* | 0 | -2.77 | 9.43E-04 | 0.42 |  |
| ENSG00000133985 | *TTC9* | 0 | -2.20 | 9.43E-04 | -1.78 |  |
| ENSG00000188042 | *ARL4C* | 0 | -1.70 | 9.43E-04 | 3.81 |  |
| ENSG00000102271 | *KLHL4* | 0 | -2.55 | 9.43E-04 | -0.76 |  |
| ENSG00000198797 | *BRINP2* | 0 | -3.35 | 9.43E-04 | -4.84 |  |
| ENSG00000139211 | *AMIGO2* | 0 | -1.99 | 9.44E-04 | 4.18 |  |
| ENSG00000090530 | *P3H2* | 0 | -1.25 | 9.68E-04 | 5.23 |  |
| ENSG00000111817 | *DSE* | 0 | -1.18 | 9.86E-04 | 5.38 |  |
| ENSG00000242258 | *LINC00996* | 0 | -2.65 | 9.94E-04 | -2.91 |  |
| ENSG00000113396 | *SLC27A6* | 0 | -1.48 | 9.96E-04 | -0.12 |  |
| ENSG00000187801 | *ZFP69B* | 0 | -1.45 | 1.02E-03 | 1.53 |  |
| ENSG00000136367 | *ZFHX2* | 0 | -1.68 | 1.02E-03 | 0.43 |  |
| ENSG00000240891 | *PLCXD2* | 0 | -1.92 | 1.03E-03 | 1.20 |  |
| ENSG00000113083 | *LOX* | 0 | -1.89 | 1.04E-03 | 8.46 |  |
| ENSG00000123500 | *COL10A1* | 0 | -2.70 | 1.04E-03 | 0.89 |  |
| ENSG00000156265 | *MAP3K7CL* | 0 | -2.01 | 1.04E-03 | 4.89 |  |
| ENSG00000052795 | *FNIP2* | 0 | -1.02 | 1.05E-03 | 3.69 |  |
| ENSG00000153993 | *SEMA3D* | 0 | -1.86 | 1.05E-03 | -0.04 |  |
| ENSG00000158050 | *DUSP2* | 0 | -1.78 | 1.05E-03 | -1.46 |  |
| ENSG00000189292 | *FAM150B* | 0 | -2.41 | 1.06E-03 | 0.77 |  |
| ENSG00000259969 | *RP11-999E24.3* | 0 | -1.79 | 1.09E-03 | 0.22 |  |
| ENSG00000182240 | *BACE2* | 0 | -1.10 | 1.09E-03 | 5.16 |  |
| ENSG00000136114 | *THSD1* | 0 | -1.14 | 1.15E-03 | 1.85 |  |
| ENSG00000158008 | *EXTL1* | 0 | -1.66 | 1.15E-03 | 2.12 |  |
| ENSG00000087589 | *CASS4* | 0 | -1.97 | 1.15E-03 | -0.25 |  |
| ENSG00000259727 | *RP11-1069G10.2* | 0 | -3.47 | 1.16E-03 | -3.92 |  |
| ENSG00000187634 | *SAMD11* | 0 | -2.01 | 1.18E-03 | 1.44 |  |
| ENSG00000105810 | *CDK6* | 0 | -1.52 | 1.21E-03 | 4.83 |  |
| ENSG00000143061 | *IGSF3* | 0 | -1.82 | 1.23E-03 | 4.61 |  |
| ENSG00000165323 | *FAT3* | 0 | -1.49 | 1.23E-03 | 0.20 |  |
| ENSG00000225783 | *MIAT* | 0 | -1.39 | 1.26E-03 | 5.32 |  |
| ENSG00000005471 | *ABCB4* | 0 | -1.69 | 1.28E-03 | 0.12 |  |
| ENSG00000214147 | *RP11-332P22.2* | 0 | -1.70 | 1.28E-03 | -0.94 |  |
| ENSG00000123572 | *NRK* | 0 | -1.60 | 1.30E-03 | 0.71 |  |
| ENSG00000198825 | *INPP5F* | 0 | -1.24 | 1.31E-03 | 6.04 |  |
| ENSG00000112852 | *PCDHB2* | 0 | -1.14 | 1.31E-03 | 0.13 |  |
| ENSG00000206052 | *DOK6* | 0 | -1.06 | 1.31E-03 | 1.88 |  |
| ENSG00000159216 | *RUNX1* | 0 | -1.58 | 1.31E-03 | 5.02 |  |
| ENSG00000259279 | *CTD-2033D15.1* | 0 | -2.56 | 1.33E-03 | -3.98 |  |
| ENSG00000072657 | *TRHDE* | 0 | -2.19 | 1.33E-03 | 3.51 |  |
| ENSG00000139174 | *PRICKLE1* | 0 | -1.41 | 1.33E-03 | 4.33 |  |
| ENSG00000134569 | *LRP4* | 0 | -1.39 | 1.36E-03 | 2.07 |  |
| ENSG00000267287 | *RP11-567M16.1* | 0 | -2.53 | 1.38E-03 | -1.69 |  |
| ENSG00000176399 | *DMRTA1* | 0 | -1.45 | 1.38E-03 | -0.53 |  |
| ENSG00000204362 | *RP11-380J14.1* | 0 | -2.20 | 1.39E-03 | -2.54 |  |
| ENSG00000198478 | *SH3BGRL2* | 0 | -1.60 | 1.42E-03 | 2.90 |  |
| ENSG00000233521 | *RP5-1172A22.1* | 0 | -2.54 | 1.42E-03 | -4.27 |  |
| ENSG00000055118 | *KCNH2* | 0 | -1.08 | 1.44E-03 | 1.37 |  |
| ENSG00000163145 | *C1QTNF7* | 0 | -1.07 | 1.45E-03 | 5.63 |  |
| ENSG00000132003 | *ZSWIM4* | 0 | -1.26 | 1.46E-03 | 3.80 |  |
| ENSG00000237356 | *AL163953.3* | 0 | -2.10 | 1.48E-03 | -0.58 |  |
| ENSG00000134198 | *TSPAN2* | 0 | -4.17 | 1.48E-03 | -1.84 |  |
| ENSG00000228495 | *LINC01013* | 0 | -4.48 | 1.48E-03 | -3.81 |  |
| ENSG00000279822 | *RP11-541M12.6* | 0 | -1.99 | 1.50E-03 | -1.19 |  |
| ENSG00000100311 | *PDGFB* | 0 | -1.66 | 1.56E-03 | 2.19 |  |
| ENSG00000184005 | *ST6GALNAC3* | 0 | -1.33 | 1.58E-03 | 2.78 |  |
| ENSG00000235649 | *MXRA5Y* | 0 | -1.55 | 1.58E-03 | -0.08 |  |
| ENSG00000260577 | *RP11-615I2.2* | 0 | -1.63 | 1.58E-03 | -1.80 |  |
| ENSG00000178776 | *C5orf46* | 0 | -3.84 | 1.59E-03 | -4.50 |  |
| ENSG00000086991 | *NOX4* | 0 | -2.56 | 1.60E-03 | 1.55 |  |
| ENSG00000231672 | *DIRC3* | 0 | -2.06 | 1.60E-03 | 2.21 |  |
| ENSG00000122877 | *EGR2* | 0 | -3.03 | 1.61E-03 | 3.89 |  |
| ENSG00000134013 | *LOXL2* | 0 | -1.21 | 1.62E-03 | 10.19 |  |
| ENSG00000134516 | *DOCK2* | 0 | -1.34 | 1.66E-03 | 1.74 |  |
| ENSG00000177791 | *MYOZ1* | 0 | -1.89 | 1.66E-03 | -1.79 |  |
| ENSG00000143126 | *CELSR2* | 0 | -1.00 | 1.67E-03 | 1.55 |  |
| ENSG00000106526 | *ACTR3C* | 0 | -1.14 | 1.67E-03 | -0.37 |  |
| ENSG00000258955 | *LINC00519* | 0 | -1.55 | 1.68E-03 | 0.36 |  |
| ENSG00000128656 | *CHN1* | 0 | -1.70 | 1.71E-03 | 5.92 |  |
| ENSG00000173898 | *SPTBN2* | 0 | -1.35 | 1.71E-03 | -0.86 |  |
| ENSG00000104881 | *PPP1R13L* | 0 | -1.39 | 1.71E-03 | 2.63 |  |
| ENSG00000120262 | *CCDC170* | 0 | -1.25 | 1.72E-03 | 1.53 |  |
| ENSG00000230500 | *MKX-AS1* | 0 | -2.08 | 1.73E-03 | -4.42 |  |
| ENSG00000259207 | *ITGB3* | 0 | -1.46 | 1.73E-03 | 4.06 |  |
| ENSG00000107518 | *ATRNL1* | 0 | -1.29 | 1.73E-03 | 3.34 |  |
| ENSG00000149201 | *CCDC81* | 0 | -2.33 | 1.74E-03 | 2.19 |  |
| ENSG00000116299 | *KIAA1324* | 0 | -1.36 | 1.80E-03 | 0.54 |  |
| ENSG00000111305 | *GSG1* | 0 | -2.98 | 1.80E-03 | -0.59 |  |
| ENSG00000152409 | *JMY* | 0 | -1.01 | 1.93E-03 | 4.67 |  |
| ENSG00000149968 | *MMP3* | 0 | -1.73 | 1.99E-03 | 8.35 |  |
| ENSG00000006016 | *CRLF1* | 0 | -3.70 | 1.99E-03 | -2.39 |  |
| ENSG00000168453 | *HR* | 0 | -1.59 | 2.02E-03 | -1.66 |  |
| ENSG00000233818 | *AP000695.4* | 0 | -2.32 | 2.02E-03 | -2.34 |  |
| ENSG00000177398 | *UMODL1* | 0 | -3.03 | 2.04E-03 | -4.95 |  |
| ENSG00000150556 | *LYPD6B* | 0 | -2.62 | 2.08E-03 | -3.91 |  |
| ENSG00000250091 | *DNAH10OS* | 0 | -1.45 | 2.08E-03 | 0.34 |  |
| ENSG00000260920 | *RP1-228H13.5* | 0 | -1.21 | 2.08E-03 | 2.31 |  |
| ENSG00000183696 | *UPP1* | 0 | -1.38 | 2.09E-03 | 5.81 |  |
| ENSG00000230630 | *DNM3OS* | 0 | -1.20 | 2.10E-03 | 5.00 |  |
| ENSG00000183010 | *PYCR1* | 0 | -1.52 | 2.13E-03 | 5.75 |  |
| ENSG00000047936 | *ROS1* | 0 | -4.27 | 2.14E-03 | -4.48 |  |
| ENSG00000154188 | *ANGPT1* | 0 | -1.49 | 2.15E-03 | 5.39 |  |
| ENSG00000243955 | *GSTA1* | 0 | -3.08 | 2.15E-03 | -3.96 |  |
| ENSG00000176532 | *PRR15* | 0 | -1.79 | 2.15E-03 | 0.25 |  |
| ENSG00000253668 | *RP11-463C14.1* | 0 | -2.04 | 2.16E-03 | -2.26 |  |
| ENSG00000236654 | *AC079780.3* | 0 | -1.73 | 2.18E-03 | -0.95 |  |
| ENSG00000149970 | *CNKSR2* | 0 | -1.85 | 2.19E-03 | 0.34 |  |
| ENSG00000255769 | *GOLGA2P10* | 0 | -1.07 | 2.20E-03 | -0.42 |  |
| ENSG00000250579 | *CTD-2297D10.2* | 0 | -3.62 | 2.22E-03 | -4.46 |  |
| ENSG00000170989 | *S1PR1* | 0 | -1.31 | 2.24E-03 | 2.36 |  |
| ENSG00000081923 | *ATP8B1* | 0 | -1.88 | 2.25E-03 | 4.31 |  |
| ENSG00000176490 | *DIRAS1* | 0 | -1.82 | 2.26E-03 | -1.29 |  |
| ENSG00000149633 | *KIAA1755* | 0 | -1.57 | 2.29E-03 | 2.55 |  |
| ENSG00000113248 | *PCDHB15* | 0 | -1.13 | 2.29E-03 | 0.63 |  |
| ENSG00000164509 | *IL31RA* | 0 | -2.07 | 2.30E-03 | 0.66 |  |
| ENSG00000133135 | *RNF128* | 0 | -2.27 | 2.30E-03 | -1.72 |  |
| ENSG00000279384 | *RP11-635L1.2* | 0 | -2.16 | 2.34E-03 | -2.27 |  |
| ENSG00000170558 | *CDH2* | 0 | -2.40 | 2.34E-03 | 4.43 |  |
| ENSG00000171435 | *KSR2* | 0 | -3.65 | 2.38E-03 | -4.20 |  |
| ENSG00000172403 | *SYNPO2* | 0 | -2.13 | 2.42E-03 | 6.31 |  |
| ENSG00000162591 | *MEGF6* | 0 | -1.87 | 2.44E-03 | 0.90 |  |
| ENSG00000135480 | *KRT7* | 0 | -1.45 | 2.48E-03 | 2.86 |  |
| ENSG00000109472 | *CPE* | 0 | -1.25 | 2.51E-03 | 6.01 |  |
| ENSG00000040275 | *SPDL1* | 0 | -1.07 | 2.54E-03 | 5.14 |  |
| ENSG00000186310 | *NAP1L3* | 0 | -1.59 | 2.55E-03 | 2.72 |  |
| ENSG00000053438 | *NNAT* | 0 | -1.41 | 2.57E-03 | 2.48 |  |
| ENSG00000136928 | *GABBR2* | 0 | -1.85 | 2.58E-03 | -0.03 |  |
| ENSG00000095739 | *BAMBI* | 0 | -1.54 | 2.60E-03 | 4.64 |  |
| ENSG00000197147 | *LRRC8B* | 0 | -1.12 | 2.62E-03 | 0.97 |  |
| ENSG00000124749 | *COL21A1* | 0 | -1.38 | 2.64E-03 | 3.56 |  |
| ENSG00000049192 | *ADAMTS6* | 0 | -1.76 | 2.65E-03 | 3.35 |  |
| ENSG00000272711 | *RP11-259N19.1* | 0 | -1.74 | 2.69E-03 | 0.56 |  |
| ENSG00000145506 | *NKD2* | 0 | -2.83 | 2.69E-03 | -1.57 |  |
| ENSG00000261115 | *TMEM178B* | 0 | -1.74 | 2.71E-03 | -1.37 |  |
| ENSG00000272789 | *RP11-286H15.1* | 0 | -2.04 | 2.73E-03 | -2.96 |  |
| ENSG00000155974 | *GRIP1* | 0 | -1.38 | 2.73E-03 | -0.64 |  |
| ENSG00000145777 | *TSLP* | 0 | -1.96 | 2.76E-03 | -1.33 |  |
| ENSG00000109705 | *NKX3-2* | 0 | -1.39 | 2.77E-03 | -0.91 |  |
| ENSG00000273312 | *RP11-425A6.5* | 0 | -2.22 | 2.77E-03 | -3.20 |  |
| ENSG00000174945 | *AMZ1* | 0 | -1.96 | 2.80E-03 | -0.22 |  |
| ENSG00000120328 | *PCDHB12* | 0 | -1.41 | 2.81E-03 | -1.61 |  |
| ENSG00000259345 | *RP11-624L4.1* | 0 | -1.77 | 2.82E-03 | -0.60 |  |
| ENSG00000113212 | *PCDHB7* | 0 | -1.15 | 2.83E-03 | 0.59 |  |
| ENSG00000005102 | *MEOX1* | 0 | -3.37 | 2.86E-03 | 1.95 |  |
| ENSG00000062038 | *CDH3* | 0 | -1.31 | 2.86E-03 | 1.97 |  |
| ENSG00000230479 | *AP000695.6* | 0 | -2.97 | 2.92E-03 | -2.80 |  |
| ENSG00000144366 | *GULP1* | 0 | -1.08 | 2.97E-03 | 5.41 |  |
| ENSG00000253837 | *RP11-177H13.2* | 0 | -1.18 | 3.02E-03 | 0.03 |  |
| ENSG00000115129 | *TP53I3* | 0 | -1.38 | 3.05E-03 | 6.46 |  |
| ENSG00000152127 | *MGAT5* | 0 | -1.12 | 3.11E-03 | 8.36 |  |
| ENSG00000232241 | *DYNLT3P1* | 0 | -1.37 | 3.12E-03 | -0.83 |  |
| ENSG00000078295 | *ADCY2* | 0 | -1.09 | 3.14E-03 | 0.47 |  |
| ENSG00000123329 | *ARHGAP9* | 0 | -3.44 | 3.16E-03 | -4.24 |  |
| ENSG00000228624 | *RP3-399L15.3* | 0 | -1.48 | 3.16E-03 | -0.71 |  |
| ENSG00000137285 | *TUBB2B* | 0 | -1.20 | 3.17E-03 | 0.11 |  |
| ENSG00000144810 | *COL8A1* | 0 | -1.99 | 3.17E-03 | 9.82 |  |
| ENSG00000235997 | *AC109642.1* | 0 | -1.88 | 3.19E-03 | -3.68 |  |
| ENSG00000131015 | *ULBP2* | 0 | -1.17 | 3.21E-03 | 0.57 |  |
| ENSG00000147883 | *CDKN2B* | 0 | -2.10 | 3.23E-03 | 4.68 |  |
| ENSG00000163491 | *NEK10* | 0 | -1.20 | 3.24E-03 | 1.01 |  |
| ENSG00000071282 | *LMCD1* | 0 | -1.19 | 3.27E-03 | 3.70 |  |
| ENSG00000152689 | *RASGRP3* | 0 | -2.27 | 3.27E-03 | 1.02 |  |
| ENSG00000149596 | *JPH2* | 0 | -1.70 | 3.27E-03 | 2.33 |  |
| ENSG00000162891 | *IL20* | 0 | -3.58 | 3.28E-03 | -3.94 |  |
| ENSG00000138758 | *SEPT11* | 0 | -1.25 | 3.29E-03 | 8.67 |  |
| ENSG00000132854 | *KANK4* | 0 | -3.38 | 3.31E-03 | -2.29 |  |
| ENSG00000154645 | *CHODL* | 0 | -2.03 | 3.39E-03 | 1.98 |  |
| ENSG00000126878 | *AIF1L* | 0 | -2.89 | 3.45E-03 | -1.08 |  |
| ENSG00000242715 | *CCDC169* | 0 | -1.50 | 3.53E-03 | -0.75 |  |
| ENSG00000169946 | *ZFPM2* | 0 | -1.25 | 3.53E-03 | -1.13 |  |
| ENSG00000060982 | *BCAT1* | 0 | -1.79 | 3.59E-03 | 5.32 |  |
| ENSG00000261295 | *RP11-524D16__A.3* | 0 | -1.17 | 3.66E-03 | 2.35 |  |
| ENSG00000108854 | *SMURF2* | 0 | -1.04 | 3.66E-03 | 6.15 |  |
| ENSG00000228612 | *HK2P1* | 0 | -1.35 | 3.66E-03 | 0.27 |  |
| ENSG00000183496 | *MEX3B* | 0 | -1.53 | 3.67E-03 | 3.45 |  |
| ENSG00000254221 | *PCDHGB1* | 0 | -1.07 | 3.69E-03 | 0.21 |  |
| ENSG00000259450 | *RP11-265N7.1* | 0 | -2.99 | 3.72E-03 | -3.45 |  |
| ENSG00000135269 | *TES* | 0 | -1.34 | 3.72E-03 | 5.07 |  |
| ENSG00000234690 | *AC073283.4* | 0 | -1.13 | 3.75E-03 | -1.85 |  |
| ENSG00000143473 | *KCNH1* | 0 | -1.89 | 3.78E-03 | 2.37 |  |
| ENSG00000178233 | *TMEM151B* | 0 | -2.62 | 3.90E-03 | -4.45 |  |
| ENSG00000107165 | *TYRP1* | 0 | -3.09 | 3.90E-03 | -2.85 |  |
| ENSG00000227268 | *KLLN* | 0 | -1.10 | 3.90E-03 | 0.52 |  |
| ENSG00000137673 | *MMP7* | 0 | -2.11 | 3.93E-03 | 2.34 |  |
| ENSG00000166396 | *SERPINB7* | 0 | -3.23 | 3.94E-03 | 0.91 |  |
| ENSG00000118523 | *CTGF* | 0 | -3.36 | 3.99E-03 | 7.56 |  |
| ENSG00000103742 | *IGDCC4* | 0 | -1.17 | 4.02E-03 | 4.27 |  |
| ENSG00000261379 | *RP11-395N3.1* | 0 | -2.53 | 4.03E-03 | -2.33 |  |
| ENSG00000183023 | *SLC8A1* | 0 | -1.62 | 4.14E-03 | 3.94 |  |
| ENSG00000185652 | *NTF3* | 0 | -1.09 | 4.19E-03 | 2.53 |  |
| ENSG00000184949 | *FAM227A* | 0 | -1.04 | 4.21E-03 | 1.66 |  |
| ENSG00000172264 | *MACROD2* | 0 | -1.36 | 4.23E-03 | -1.50 |  |
| ENSG00000102359 | *SRPX2* | 0 | -1.01 | 4.24E-03 | 6.92 |  |
| ENSG00000139926 | *FRMD6* | 0 | -1.13 | 4.27E-03 | 7.65 |  |
| ENSG00000120217 | *CD274* | 0 | -1.30 | 4.28E-03 | 3.64 |  |
| ENSG00000224963 | *U82695.9* | 0 | -2.24 | 4.33E-03 | -3.90 |  |
| ENSG00000070404 | *FSTL3* | 0 | -1.30 | 4.34E-03 | 3.54 |  |
| ENSG00000169583 | *CLIC3* | 0 | -1.29 | 4.42E-03 | 1.74 |  |
| ENSG00000203756 | *TMEM244* | 0 | -3.30 | 4.43E-03 | -4.81 |  |
| ENSG00000259495 | *RP11-210M15.2* | 0 | -2.03 | 4.44E-03 | -4.86 |  |
| ENSG00000154553 | *PDLIM3* | 0 | -1.52 | 4.47E-03 | -2.53 |  |
| ENSG00000133816 | *MICAL2* | 0 | -1.19 | 4.55E-03 | 6.84 |  |
| ENSG00000164741 | *DLC1* | 0 | -1.22 | 4.56E-03 | 6.73 |  |
| ENSG00000122574 | *WIPF3* | 0 | -1.64 | 4.58E-03 | -5.23 |  |
| ENSG00000140832 | *MARVELD3* | 0 | -3.90 | 4.66E-03 | -4.73 |  |
| ENSG00000140416 | *TPM1* | 0 | -1.73 | 4.73E-03 | 8.75 |  |
| ENSG00000076706 | *MCAM* | 0 | -1.78 | 4.76E-03 | 2.57 |  |
| ENSG00000150938 | *CRIM1* | 0 | -1.11 | 4.77E-03 | 7.35 |  |
| ENSG00000143882 | *ATP6V1C2* | 0 | -1.74 | 4.78E-03 | -1.03 |  |
| ENSG00000162614 | *NEXN* | 0 | -1.10 | 4.86E-03 | 5.66 |  |
| ENSG00000167767 | *KRT80* | 0 | -1.88 | 4.87E-03 | 0.42 |  |
| ENSG00000149591 | *TAGLN* | 0 | -2.01 | 4.91E-03 | 9.25 |  |
| ENSG00000101680 | *LAMA1* | 0 | -1.07 | 4.93E-03 | 5.65 |  |
| ENSG00000164604 | *GPR85* | 0 | -1.07 | 4.93E-03 | 0.56 |  |
| ENSG00000231426 | *RP5-899B16.1* | 0 | -1.71 | 4.98E-03 | -2.27 |  |
| ENSG00000117586 | *TNFSF4* | 0 | -1.46 | 5.02E-03 | 5.70 |  |
| ENSG00000280143 | *AP000892.6* | 0 | -2.11 | 5.03E-03 | 3.13 |  |
| ENSG00000173320 | *STOX2* | 0 | -2.03 | 5.07E-03 | -2.94 |  |
| ENSG00000272622 | *RP11-395N3.2* | 0 | -1.47 | 5.08E-03 | -0.17 |  |
| ENSG00000133808 | *MICALCL* | 0 | -1.87 | 5.09E-03 | -2.22 |  |
| ENSG00000180998 | *GPR137C* | 0 | -1.24 | 5.22E-03 | 1.26 |  |
| ENSG00000105880 | *DLX5* | 0 | -1.70 | 5.29E-03 | -4.48 |  |
| ENSG00000115363 | *EVA1A* | 0 | -1.00 | 5.31E-03 | 3.97 |  |
| ENSG00000272168 | *CASC15* | 0 | -1.85 | 5.34E-03 | -0.16 |  |
| ENSG00000197646 | *PDCD1LG2* | 0 | -1.23 | 5.42E-03 | 4.19 |  |
| ENSG00000177494 | *ZBED2* | 0 | -2.99 | 5.42E-03 | -3.71 |  |
| ENSG00000119915 | *ELOVL3* | 0 | -3.05 | 5.45E-03 | -4.60 |  |
| ENSG00000142224 | *IL19* | 0 | -1.47 | 5.52E-03 | -0.92 |  |
| ENSG00000104435 | *STMN2* | 0 | -1.61 | 5.53E-03 | -1.05 |  |
| ENSG00000136160 | *EDNRB* | 0 | -3.41 | 5.55E-03 | 2.58 |  |
| ENSG00000152092 | *ASTN1* | 0 | -1.66 | 5.56E-03 | -4.39 |  |
| ENSG00000131019 | *ULBP3* | 0 | -1.42 | 5.59E-03 | 0.25 |  |
| ENSG00000261143 | *ADAMTS7P3* | 0 | -1.34 | 5.62E-03 | -0.24 |  |
| ENSG00000113578 | *FGF1* | 0 | -1.49 | 5.62E-03 | 3.05 |  |
| ENSG00000123570 | *RAB9B* | 0 | -1.01 | 5.63E-03 | 0.38 |  |
| ENSG00000175600 | *SUGCT* | 0 | -1.20 | 5.70E-03 | 1.83 |  |
| ENSG00000237819 | *AC002454.1* | 0 | -2.56 | 5.79E-03 | -3.60 |  |
| ENSG00000166689 | *PLEKHA7* | 0 | -1.39 | 5.80E-03 | -2.04 |  |
| ENSG00000216775 | *RP1-152L7.5* | 0 | -1.12 | 5.92E-03 | 3.61 |  |
| ENSG00000046889 | *PREX2* | 0 | -1.49 | 5.93E-03 | 0.35 |  |
| ENSG00000169393 | *ELSPBP1* | 0 | -3.31 | 5.94E-03 | -4.93 |  |
| ENSG00000182771 | *GRID1* | 0 | -1.19 | 6.04E-03 | -2.72 |  |
| ENSG00000085276 | *MECOM* | 0 | -1.28 | 6.08E-03 | -0.61 |  |
| ENSG00000137968 | *SLC44A5* | 0 | -1.32 | 6.17E-03 | -1.86 |  |
| ENSG00000128165 | *ADM2* | 0 | -4.07 | 6.17E-03 | -0.83 |  |
| ENSG00000047662 | *FAM184B* | 0 | -1.96 | 6.17E-03 | -4.49 |  |
| ENSG00000017427 | *IGF1* | 0 | -1.53 | 6.18E-03 | -3.06 |  |
| ENSG00000103257 | *SLC7A5* | 0 | -3.04 | 6.18E-03 | 3.58 |  |
| ENSG00000101115 | *SALL4* | 0 | -2.68 | 6.29E-03 | -2.29 | |
| ENSG00000235770 | *LINC00607* | 0 | -2.78 | 6.31E-03 | -1.40 | |
| ENSG00000261189 | *RP3-512B11.3* | 0 | -2.17 | 6.35E-03 | -0.90 | |
| ENSG00000111424 | *VDR* | 0 | -1.27 | 6.36E-03 | 3.25 | |
| ENSG00000033122 | *LRRC7* | 0 | -2.07 | 6.43E-03 | -3.91 | |
| ENSG00000176971 | *FIBIN* | 0 | -2.83 | 6.48E-03 | 3.79 | |
| ENSG00000236393 | *RP11-320G24.1* | 0 | -1.40 | 6.49E-03 | 0.88 | |
| ENSG00000255725 | *TDGP1* | 0 | -1.02 | 6.57E-03 | 0.53 | |
| ENSG00000224025 | *RP11-274B18.3* | 0 | -1.38 | 6.59E-03 | -0.97 | |
| ENSG00000165495 | *PKNOX2* | 0 | -1.31 | 6.59E-03 | 2.58 | |
| ENSG00000111907 | *TPD52L1* | 0 | -1.04 | 6.75E-03 | -1.30 | |
| ENSG00000117069 | *ST6GALNAC5* | 0 | -1.14 | 6.76E-03 | 3.26 | |
| ENSG00000244694 | *PTCHD4* | 0 | -1.97 | 6.77E-03 | 2.20 | |
| ENSG00000248801 | *C8orf34-AS1* | 0 | -3.01 | 6.79E-03 | -4.32 | |
| ENSG00000177283 | *FZD8* | 0 | -1.76 | 6.86E-03 | 4.83 | |
| ENSG00000070193 | *FGF10* | 0 | -1.57 | 6.87E-03 | -2.55 | |
| ENSG00000170396 | *ZNF804A* | 0 | -1.04 | 6.96E-03 | 3.41 | |
| ENSG00000115525 | *ST3GAL5* | 0 | -1.02 | 7.00E-03 | 6.96 | |
| ENSG00000214517 | *PPME1* | 0 | -1.15 | 7.02E-03 | 6.39 | |
| ENSG00000175175 | *PPM1E* | 0 | -1.72 | 7.36E-03 | -3.19 | |
| ENSG00000240032 | *RP11-274H2.3* | 0 | -2.52 | 7.36E-03 | -2.94 | |
| ENSG00000102385 | *DRP2* | 0 | -1.06 | 7.36E-03 | 1.15 | |
| ENSG00000260428 | *SCX* | 0 | -2.54 | 7.46E-03 | -0.09 | |
| ENSG00000120949 | *TNFRSF8* | 0 | -1.92 | 7.47E-03 | -2.48 | |
| ENSG00000180611 | *MB21D2* | 0 | -1.11 | 7.57E-03 | 3.37 | |
| ENSG00000163092 | *XIRP2* | 0 | -4.85 | 7.80E-03 | -3.50 | |
| ENSG00000134917 | *ADAMTS8* | 0 | -1.16 | 8.08E-03 | -1.82 | |
| ENSG00000113070 | *HBEGF* | 0 | -2.19 | 8.19E-03 | 1.15 | |
| ENSG00000120332 | *TNN* | 0 | -2.75 | 8.20E-03 | -5.09 | |
| ENSG00000229373 | *LINC00452* | 0 | -2.13 | 8.22E-03 | -4.03 | |
| ENSG00000172554 | *SNTG2* | 0 | -1.26 | 8.22E-03 | -0.65 | |
| ENSG00000174403 | *C20orf166-AS1* | 0 | -3.80 | 8.22E-03 | -5.24 | |
| ENSG00000007062 | *PROM1* | 0 | -1.41 | 8.23E-03 | -4.03 | |
| ENSG00000240708 | *RP11-64C1.1* | 0 | -3.17 | 8.30E-03 | -4.08 | |
| ENSG00000184544 | *DHRS7C* | 0 | -3.08 | 8.35E-03 | -3.41 |  |
| ENSG00000158457 | *TSPAN33* | 0 | -1.49 | 8.38E-03 | -0.07 |  |
| ENSG00000134762 | *DSC3* | 0 | -1.62 | 8.46E-03 | -2.03 |  |
| ENSG00000106483 | *SFRP4* | 0 | -1.69 | 8.51E-03 | -4.19 |  |
| ENSG00000204967 | *PCDHA4* | 0 | -1.29 | 8.63E-03 | -1.32 |  |
| ENSG00000251493 | *FOXD1* | 0 | -1.01 | 8.72E-03 | 1.02 |  |
| ENSG00000274307 | *RP11-345J18.2* | 0 | -1.61 | 8.77E-03 | -3.46 |  |
| ENSG00000183508 | *FAM46C* | 0 | -1.39 | 8.78E-03 | 4.16 |  |
| ENSG00000171877 | *FRMD5* | 0 | -1.86 | 8.87E-03 | -2.84 |  |
| ENSG00000203706 | *SERTAD4-AS1* | 0 | -1.30 | 8.89E-03 | 2.59 |  |
| ENSG00000139044 | *B4GALNT3* | 0 | -1.94 | 9.00E-03 | -3.60 |  |
| ENSG00000087494 | *PTHLH* | 0 | -2.05 | 9.00E-03 | 1.53 |  |
| ENSG00000162630 | *B3GALT2* | 0 | -1.69 | 9.06E-03 | 1.16 |  |
| ENSG00000175832 | *ETV4* | 0 | -1.28 | 9.33E-03 | 3.97 |  |
| ENSG00000171621 | *SPSB1* | 0 | -1.06 | 9.35E-03 | 4.92 |  |
| ENSG00000203952 | *CCDC160* | 0 | -1.75 | 9.49E-03 | -4.65 |  |
| ENSG00000196758 | *AC079612.1* | 0 | -3.63 | 9.56E-03 | -4.30 |  |
| ENSG00000113361 | *CDH6* | 0 | -1.94 | 9.62E-03 | 1.69 |  |
| ENSG00000179841 | *AKAP5* | 0 | -1.65 | 9.69E-03 | 1.40 |  |
| ENSG00000017483 | *SLC38A5* | 0 | -1.16 | 9.72E-03 | 4.63 |  |
| ENSG00000279030 | *RP11-212I21.3* | 0 | -1.83 | 9.72E-03 | -3.86 |  |
| ENSG00000172575 | *RASGRP1* | 0 | -1.98 | 9.77E-03 | -1.54 |  |
| ENSG00000260101 | *RP11-568N6.1* | 0 | -1.23 | 9.81E-03 | -1.30 |  |
| ENSG00000165349 | *SLC7A3* | 0 | -1.08 | 9.88E-03 | 1.14 |  |
| ENSG00000171208 | *NETO2* | 0 | -1.08 | 9.95E-03 | 3.27 |  |

**Supplementary table 3**

**Biological functional terms enriched in the differentially expressed genes using Gene ontology analysis.**

| GO-Term/Gene Count | Gene | Fold enriched | Benjamini (p<0.05) |
| --- | --- | --- | --- |
| extracellular matrix organization/ 39 | *ABI3BP, ADAMTSL4, EGFLAM, COMP, CSGALNACT1, COL1A1, COL4A1, COL4A2, COL8A1, CL10A1, COL11A1, CRISPLD2, ELN, FN1, FBLN1, FBLN5, FOXF2, HPSE, HAPLN1, ITGA1, ITGA4, TGB3, ITGB4, ITGB5, ITGB8, ICAM4, LAMA1, LAMA4, LOX, MFAP5, NDNF, PXDN, PDGFB, TNC, THBS1, VCAM1, VCAN, VIT, VWA1* | 3.69 | 1.63E-08 |
| cell adhesion/ 61 | *ADAM12, CX3C1, CD24, CD36, CASS4, NUAK1, PPFIBP1, SPOCK1, ARVCF, CDH13, CDH2, CDH3, CDH6, CASK, CEACAM1, COMP, , EMILIN2, COL1A1, COL6A6, COL8A1, COL12A1, CSF3R, CTGF, DSC3, EFNB2, FN1, FBLN7, HAPLN1, ITGA4, ITGB3, ITGB4, ITGB5, ITGB8, ICAM4, LAMA1, LAMA4, LOXL2, MYBPH, NCAM1, NEDD9, NRP2, OLR1, PLXNC1, PTPRU, PCDHA4, PCDHB12, PCDHB15, PCDHB2, SELL, SCN1B, SORBS1, SORBS2, S1PR1, SPON1, SPON2, TNC, TNXB, THBS1, TINAGL1, VCAM1, VCAN* | 2.46 | 3.00E-07 |
| positive regulation of cell proliferation/ 57 | *BAMBI, GLI1, NKX3-1, SOX4, ST8SIA1, WNT2, ADRA1D, ADRA2A, AKR1C3, CDC25B, CLDN7, CSF1, CSF3, CTGF, CRLF1, DPP4, EDN1, EDNRB, EFNB2, FGF1, FGF20, FGF7, FGFR3, FN1, GREM1, HBEGF, HGF, ID4, IGF1, IGF2, IRS1, IL15, IL31RA, IL6R, IL6ST, LIFR, LEF1, NRG1, NTF3, NAMPT, OSR2, PTH1R, PTHLH, PGF, PDGFC, PDGFB, RAC2, RARB, SCX, SFRP1, SLC25A27, TNC, THBS1, TGFB2, TNFSF13B, TNFSF4, VEGFD* | 2.27 | 1.83E-05 |
| angiogenesis/ 35 | *ARHGAP22, TEK, TNFAIP2, ACVRL1, ADM2, ANGPT1, APOD, CEACAM1, COL4A2, COL8A1, CTGF, EMCN, EPAS1, FGF1, FGF10, FN1, ENPEP, HAND2, HS6ST1, HIF3A, JAG1, MMP2, NOV, NRXN3, NDNF, NRP2 ,PDE3B, PGF, PLXND1, PTGS2, S1PR1, SRPX2, TGFB2, TMEM100, VEGFD* | 2.91 | 3.57E-05 |
| collagen catabolic process/ 16 | *ADAMTS14, CTSL, COL1A1, COL4A1, COL4A2, COL6A6, COL8A1, COL10A1, COL11A1, COL12A1, MMP10, MMP11, MMP15, MMP2, MMP3, MMP7* | 4.63 | 8.35E-04 |
| positive regulation of gene expression/ 35 | *KLF4, NKX3-1, SMAD3, WNT11, ACTC1, ADM2, ANK3, APOB, CDH3, CSF1, CTGF, CDK6, EPHX2, FN1, FBLN1, FOXD1, HAND2, INHBA, ITGB8, IL7R, LEF1, MAPK8, NFIL3, OSR1, OSR2, PDGFB, RIMS2, SCX, SFRP4, STAR, TNC, TLR3, TGFB2, TWIST1, VDR* | 2.48 | 1.15E-03 |
| positive regulation of cell migration/ 28 | *CXCL16, CD274, F2RL1, GLI1, SMAD3, WNT11, ADRA2A, CDH13, CEMIP, F3, COL1A1, CSF1, EDN1, FGF1, GRB7, HBEGF, HGF, IGF1, LEF1, NTF3, PDGFC, PDGFB, ROR2, SEMA3D, SEMA3F, SEMA7A, S1PR1, THBS1* | 2.82 | 1.08E-03 |
| collagen fibril organization/ 12 | *ADAMTS14, COL1A1, COL11A1, COL12A1, FMOD, GREM1, LOXL2, LOX, MMP11, SCX, TNXB, TGFB2* | 5.70 | 2.04E-03 |
| positive regulation of peptidyl-tyrosine phosphorylation/ 17 | *CD36, LRP4, ANGPT1, CSF3, ENPP2, EFNA5, FGF10, FGF7, HGF, IGF1, IGF2, ITGB3, IL6R, NRG1, NTF3, PLPP3, PDGFB* | 3.84 | 2.69E-03 |
| endodermal cell differentiation/ 10 | *COL4A2, COL8A1, COL11A1, COL12A1, FN1, INHBA, ITGA4, ITGB5, MMP15, MMP2* | 6.86 | 2.68E-03 |
| signal transduction/ 98 | *AKAP5, CCL8, CD274, CD38, CD83, DLC1, FYB, GNG11, GPR85, GULP1, LANCL3, MET, NDP, RASGRP1, RASGRP2, ARHGAP15, ARHGAP20, ARHGAP22, ARHGAP44, ARHGAP6, ARHGAP9, S100A6, STAC, SP110, SPOCK1, STARD8, TEK, TNFRSF21, TNFRSF8, ASIC1, ACVRL1, ADRA2A, ANK1, ANK3, APOL3, ARRDC2, CRABP2, CHN1, CLIC2, CLIC3, CHRNA1, CHRNA9, CSF2RB, CSF3R, C3, EPAS1, ESR1, FGF1, FGF13, FGF20, FGF7, GRP, GREM1, GRB10, GRB7, HBEGF, HUNK, IGF1, IGFBP1, IGFBP2, IGFBP6, IRS1, IL15RA, IL15, IL19, IL7R, KALRN, KIF13B, LY75, MAPK10, NEDD9, NRXN3, NTF3, NAMPT, NOD1, PTCH2, PPARG, PLCXD2, PDE3B, PGF, PDPN, PTGES, RAC2, ROR2, RARB, RPS6KA6, SAV1, SECTM1, SIGIRR, SORL1, SALL4, SDC4, TENM3, TRHDE, TLR3, TNFSF13B, TNFSF4, VDR* | 1.56 | 3.05E-03 |
| homophilic cell adhesion via plasma membrane adhesion molecules/ 24 | *FAT3, FAT4, AMIGO2, CDH13, CDH2, CDH3, CDH6, CELSR2, CDHR3, CLSTN2, CEACAM1, CADM1, DCHS1, DSC3, PCDHA4, PCDHB10, PCDHB12, PCDHB15, PCDHB2, PCDHB5, PCDHB7, PCDHGB1, SDK1, TENM3* | 2.82 | 4.06E-03 |
| skeletal system development/ 22 | *NKX3-2, SOX4, ALPL, COMP, CHRD, COL1A1, COL10A1, COL12A1, DLX5, EXTL1, FGFR3, FGFRL1, HAPLN1, IGF1, IGF2, PTH1R, PTHLH, PRELP, TGFB2, TP63, VCAN, VDR* | 2.98 | 3.96E-03 |
| wound healing/ 16 | *SMAD3, VANGL2, WNT5A, CDH3, DSP, FGF10, FN1, ITGB3, LOX, MAP3K5, NRG1, SCARB1, SDC4, TNC, TGFB2, TPM1* | 3.71 | 5.52E-03 |
| cellular response to transforming growth factor beta stimulus/ 12 | *CLEC3B, NOX4, PPARGC1A, WNT2, WNT5A, ACVRL1, COL1A1, COL4A2, EDN1, SCX, SFRP1, STAR* | 4.54 | 1.15E-02 |
| epithelial to mesenchymal transition/ 10 | *S100A4, WNT11, WNT5A, FOXF2, HGF, LEF1, LOXL2, RFLNB, TGFB2, TGFBR3* | 5.45 | 1.33E-02 |
| positive regulation of epithelial cell proliferation/ 13 | *HTRA1, CYP7B1, DLX5, FGF1, FGF10, FGF7, HYAL1, IGF1, OSR1, OSR2, SFRP1, SCN5A, TWIST1* | 4.02 | 1.56E-02 |
| negative regulation of smooth muscle cell proliferation/ 9 | *KLF4, NDRG4, PPARGC1A, TNFAIP3, APOD, CTNNBIP1, IL15, NPR1, PPARG* | 5.75 | 2.21E-02 |
| positive regulation of GTPase activity/ 53 | *AGFG2, ADAP1, ADAP2, CCL8, CX3CL1, CD40, CDC42EP4, DLC1, FGD6, GFRA1, JAK3, LLGL2, MCF2L2, RASGRP1, RASGRP2, RASGRP3, ARHGAP15, ARHGAP20, ARHGAP22, ARHGAP44, ARHGAP6, ARHGAP9, ARHGEF28, STARD8, TEK, WNT11, ADGRB3, ALDH1A1, ANGPT1, CHN1, CSF2RB, DOCK2, DOCK8, DOCK9, FGF1, FGF10, FGF20, FGF7, FGFR3, FNBP1L, HBEGF, IRS1, KALRN, NCAM1, NRG1, NTRK1, PREX2, PDGFB, RGL3, RGS22, SFRP1, SPTBN2, S1PR1* | 1.74 | 2.23E-02 |
| face morphogenesis/ 9 | *CLDN5, COL1A1, CRISPLD2, DKK1, DLX5, LEF1, MMP2, SCX, TGFB2* | 5.56 | 2.58E-02 |
| positive regulation of angiogenesis/ 18 | *CX3CL1, GATA6, TEK, WNT5A, ACVRL1, ADM2, F3, C3, FGF1, GREM1, HGF, HYAL1, NTRK1, PGF, THBS1, TWIST1, VEGFD, VASH2* | 2.90 | 2.47E-02 |
| male gonad development/ 16 | *GATA6, NKX3-1, WT1, WNT2B, WNT5A, AKR1C3, ESR1, FSTL3, INHBA, MAMLD1, NR0B1, SFRP1, STAR, TLR3, TFAP2C, TGFB2* | 3.15 | 2.41E-02 |
| negative regulation of cell proliferation/ 40 | *ADAMTS8, DLC1, KLF4, NOX4, NKX3-1, NRK, RERG, SOX4, TNFRSF8, WT1, ACVRL1, ADARB1, CDH13, CDK6, CDKN2B, FGF10, FGFRL1, INHBA, IGFBP6, ITGA1, IFITM1, MSX2, NTRK1, PTH1R, PTHLH, P3H2, PTGES, PTGS2, PTPRU, RARB, RARRES1, RARRES3, SCIN, SFRP1, SFRP4, TNS2, TES, TGFB2, VDR* | 1.87 | 3.33E-02 |
| negative regulation of cell growth/ 18 | *RERG, SMAD3, WFDC1, WT1, WNT11, ACVRL1, BDKRB1, GREM1, HYAL1, INHBA, NPR1, NOV, OSGIN2, PPARG, SFRP1, SLIT2, TGFB2, TNK1* | 2.76 | 4.02E-02 |
| positive regulation of MAP kinase activity/ 12 | *CD24, CD40, DIRAS1, NOX4, NEK10, RASGRP1, ADRA2A, EDN1, FGF1, PDGFC, PDGFB, TPD52L1* | 3.77 | 3.99E-02 |
| positive regulation of endothelial cell proliferation/ 13 | *TEK, WNT2, WNT5A, ACVRL1, CDH13, F3, ITGB3, NRP2, PGF, PDGFB, STAT5A, VEGFD, VASH2* | 3.49 | 4.05E-02 |
| endochondral ossification/ 8 | *TEK, ALPL, CSGALNACT1, COL1A1, COL10A1, DLX5, FGFR3, SCX* | 5.70 | 4.67E-02 |
| activation of protein kinase B activity/ 8 | *WNT5A, ADRA2A, ADRA2C, FGF1, IGF1, NRG1, NTF3, PDGFB* | 5.70 | 4.67E-02 |
| positive regulation of Ras protein signal transduction/ 7 | *RASGRP1, CSF1, DGKI, FGF10, IGF1, NRG1, NTRK1* | 6.83 | 4.66E-02 |
| axon guidance/ 21 | *WNT5A, ANK3, DLX5, Drosophila, ENAH, EFNA5, EFNB2, FLRT3, FOXD1, KIF26B, KIF5C, MATN2, NCAM1, NRXN3, NRP2, NTRK1, SEMA3F, SLIT2, SCN1B, SPTBN2, SPON2, TGFB2* | 2.45 | 4.51E-02 |
| negative chemotaxis/ 9 | *EPHA7, EFNA5, FLRT3, ITGB3, NRP2, SEMA3D, SEMA3F, SEMA7A, SLIT2* | 4.91 | 4.37E-02 |
| inflammatory response/ 38 | *CCL8, CCRL2, CXCL2, CXCL3, CXCR4, CD14, CD40, F2RL1, GPR68, NOX4, TRIL, TNFAIP3, TNFRSF21, TNFRSF8, XCR1, AOX1, APOL3, BDKRB1, BDKRB2, CSF1, C3, EPHX2, GGT5, HRH1, HDAC9, HYAL1, IL15, IL19, LXN, LY75, NOD1, OLR1, PTX3, PTGS2, SEMA7A, TSPAN2, THBS1, TLR3* | 1.86 | 4.25E-02 |
| cellular response to lipopolysaccharide/17 | *CXCL16, CD14, CD36, CD40, PPARGC1A, TNFAIP3, WNT5A, ZFP36, CSF3, CMPK2, EDNRB, MAPK8, NR1H3, SPON2, STAR, TFPI, TNFSF4* | 2.79 | 4.14E-02 |
| cell morphogenesis/ 12 | *FRY, NOX4, GREM1, HGF, IL7R, NRG1, PDPN, SHROOM2, SHROOM3, TENM3, TGFB2, VDR* | 3.59 | 4.72E-02 |
| positive regulation of ERK1 and ERK2 cascade/ 22 | *BMPER, CCL8, CX3CL1, CD36, F2RL1, NOX4, NDRG4, RASGRP1, TEK, ANGPT1, CTGF, FAM150B, FGF1, FGF10, FGF20, FGFR3, HAND2, NTRK1, NOD1, PDGFC, PDGFB, SEMA7A* | 2.33 | 4.97E-02 |
| extracellular matrix organization/ 39 | *ABI3BP, ADAMTSL4, EGFLAM, COMP, CSGALNACT1, COL1A1, COL4A1, COL4A2, COL8A1, CL10A1, COL11A1, CRISPLD2, ELN, FN1, FBLN1, FBLN5, FOXF2, HPSE, HAPLN1, ITGA1, ITGA4, TGB3, ITGB4, ITGB5, ITGB8, ICAM4, LAMA1, LAMA4, LOX, MFAP5, NDNF, PXDN, PDGFB, TNC, THBS1, VCAM1, VCAN, VIT, VWA1* | 3.69 | 1.63E-08 |
| cell adhesion/ 61 | *ADAM12, CX3C1, CD24, CD36, CASS4, NUAK1, PPFIBP1, SPOCK1, ARVCF, CDH13, CDH2, CDH3, CDH6, CASK, CEACAM1, COMP, , EMILIN2, COL1A1, COL6A6, COL8A1, COL12A1, CSF3R, CTGF, DSC3, EFNB2, FN1, FBLN7, HAPLN1, ITGA4, ITGB3, ITGB4, ITGB5, ITGB8, ICAM4, LAMA1, LAMA4, LOXL2, MYBPH, NCAM1, NEDD9, NRP2, OLR1, PLXNC1, PTPRU, PCDHA4, PCDHB12, PCDHB15, PCDHB2, SELL, SCN1B, SORBS1, SORBS2, S1PR1, SPON1, SPON2, TNC, TNXB, THBS1, TINAGL1, VCAM1, VCAN* | 2.46 | 3.00E-07 |
| positive regulation of cell proliferation/ 57 | *BAMBI, GLI1, NKX3-1, SOX4, ST8SIA1, WNT2, ADRA1D, ADRA2A, AKR1C3, CDC25B, CLDN7, CSF1, CSF3, CTGF, CRLF1, DPP4, EDN1, EDNRB, EFNB2, FGF1, FGF20, FGF7, FGFR3, FN1, GREM1, HBEGF, HGF, ID4, IGF1, IGF2, IRS1, IL15, IL31RA, IL6R, IL6ST, LIFR, LEF1, NRG1, NTF3, NAMPT, OSR2, PTH1R, PTHLH, PGF, PDGFC, PDGFB, RAC2, RARB, SCX, SFRP1, SLC25A27, TNC, THBS1, TGFB2, TNFSF13B, TNFSF4, VEGFD* | 2.27 | 1.83E-05 |
| angiogenesis/ 35 | *ARHGAP22, TEK, TNFAIP2, ACVRL1, ADM2, ANGPT1, APOD, CEACAM1, COL4A2, COL8A1, CTGF, EMCN, EPAS1, FGF1, FGF10, FN1, ENPEP, HAND2, HS6ST1, HIF3A, JAG1, MMP2, NOV, NRXN3, NDNF, NRP2 ,PDE3B, PGF, PLXND1, PTGS2, S1PR1, SRPX2, TGFB2, TMEM100, VEGFD* | 2.91 | 3.57E-05 |
| collagen catabolic process/ 16 | *ADAMTS14, CTSL, COL1A1, COL4A1, COL4A2, COL6A6, COL8A1, COL10A1, COL11A1, COL12A1, MMP10, MMP11, MMP15, MMP2, MMP3, MMP7* | 4.63 | 8.35E-04 |
| positive regulation of gene expression/ 35 | *KLF4, NKX3-1, SMAD3, WNT11, ACTC1, ADM2, ANK3, APOB, CDH3, CSF1, CTGF, CDK6, EPHX2, FN1, FBLN1, FOXD1, HAND2, INHBA, ITGB8, IL7R, LEF1, MAPK8, NFIL3, OSR1, OSR2, PDGFB, RIMS2, SCX, SFRP4, STAR, TNC, TLR3, TGFB2, TWIST1, VDR* | 2.48 | 1.15E-03 |
| positive regulation of cell migration/ 28 | *CXCL16, CD274, F2RL1, GLI1, SMAD3, WNT11, ADRA2A, CDH13, CEMIP, F3, COL1A1, CSF1, EDN1, FGF1, GRB7, HBEGF, HGF, IGF1, LEF1, NTF3, PDGFC, PDGFB, ROR2, SEMA3D, SEMA3F, SEMA7A, S1PR1, THBS1* | 2.82 | 1.08E-03 |
| collagen fibril organization/ 12 | *ADAMTS14, COL1A1, COL11A1, COL12A1, FMOD, GREM1, LOXL2, LOX, MMP11, SCX, TNXB, TGFB2* | 5.70 | 2.04E-03 |
| positive regulation of peptidyl-tyrosine phosphorylation/ 17 | *CD36, LRP4, ANGPT1, CSF3, ENPP2, EFNA5, FGF10, FGF7, HGF, IGF1, IGF2, ITGB3, IL6R, NRG1, NTF3, PLPP3, PDGFB* | 3.84 | 2.69E-03 |
| endodermal cell differentiation/ 10 | *COL4A2, COL8A1, COL11A1, COL12A1, FN1, INHBA, ITGA4, ITGB5, MMP15, MMP2* | 6.86 | 2.68E-03 |
| signal transduction/ 98 | *AKAP5, CCL8, CD274, CD38, CD83, DLC1, FYB, GNG11, GPR85, GULP1, LANCL3, MET, NDP, RASGRP1, RASGRP2, ARHGAP15, ARHGAP20, ARHGAP22, ARHGAP44, ARHGAP6, ARHGAP9, S100A6, STAC, SP110, SPOCK1, STARD8, TEK, TNFRSF21, TNFRSF8, ASIC1, ACVRL1, ADRA2A, ANK1, ANK3, APOL3, ARRDC2, CRABP2, CHN1, CLIC2, CLIC3, CHRNA1, CHRNA9, CSF2RB, CSF3R, C3, EPAS1, ESR1, FGF1, FGF13, FGF20, FGF7, GRP, GREM1, GRB10, GRB7, HBEGF, HUNK, IGF1, IGFBP1, IGFBP2, IGFBP6, IRS1, IL15RA, IL15, IL19, IL7R, KALRN, KIF13B, LY75, MAPK10, NEDD9, NRXN3, NTF3, NAMPT, NOD1, PTCH2, PPARG, PLCXD2, PDE3B, PGF, PDPN, PTGES, RAC2, ROR2, RARB, RPS6KA6, SAV1, SECTM1, SIGIRR, SORL1, SALL4, SDC4, TENM3, TRHDE, TLR3, TNFSF13B, TNFSF4, VDR* | 1.56 | 3.05E-03 |
| homophilic cell adhesion via plasma membrane adhesion molecules/ 24 | *FAT3, FAT4, AMIGO2, CDH13, CDH2, CDH3, CDH6, CELSR2, CDHR3, CLSTN2, CEACAM1, CADM1, DCHS1, DSC3, PCDHA4, PCDHB10, PCDHB12, PCDHB15, PCDHB2, PCDHB5, PCDHB7, PCDHGB1, SDK1, TENM3* | 2.82 | 4.06E-03 |
| skeletal system development/ 22 | *NKX3-2, SOX4, ALPL, COMP, CHRD, COL1A1, COL10A1, COL12A1, DLX5, EXTL1, FGFR3, FGFRL1, HAPLN1, IGF1, IGF2, PTH1R, PTHLH, PRELP, TGFB2, TP63, VCAN, VDR* | 2.98 | 3.96E-03 |
| wound healing/ 16 | *SMAD3, VANGL2, WNT5A, CDH3, DSP, FGF10, FN1, ITGB3, LOX, MAP3K5, NRG1, SCARB1, SDC4, TNC, TGFB2, TPM1* | 3.71 | 5.52E-03 |
| cellular response to transforming growth factor beta stimulus/ 12 | *CLEC3B, NOX4, PPARGC1A, WNT2, WNT5A, ACVRL1, COL1A1, COL4A2, EDN1, SCX, SFRP1, STAR* | 4.54 | 1.15E-02 |
| epithelial to mesenchymal transition/ 10 | *S100A4, WNT11, WNT5A, FOXF2, HGF, LEF1, LOXL2, RFLNB, TGFB2, TGFBR3* | 5.45 | 1.33E-02 |
| positive regulation of epithelial cell proliferation/ 13 | *HTRA1, CYP7B1, DLX5, FGF1, FGF10, FGF7, HYAL1, IGF1, OSR1, OSR2, SFRP1, SCN5A, TWIST1* | 4.02 | 1.56E-02 |
| negative regulation of smooth muscle cell proliferation/ 9 | *KLF4, NDRG4, PPARGC1A, TNFAIP3, APOD, CTNNBIP1, IL15, NPR1, PPARG* | 5.75 | 2.21E-02 |
| positive regulation of GTPase activity/ 53 | *AGFG2, ADAP1, ADAP2, CCL8, CX3CL1, CD40, CDC42EP4, DLC1, FGD6, GFRA1, JAK3, LLGL2, MCF2L2, RASGRP1, RASGRP2, RASGRP3, ARHGAP15, ARHGAP20, ARHGAP22, ARHGAP44, ARHGAP6, ARHGAP9, ARHGEF28, STARD8, TEK, WNT11, ADGRB3, ALDH1A1, ANGPT1, CHN1, CSF2RB, DOCK2, DOCK8, DOCK9, FGF1, FGF10, FGF20, FGF7, FGFR3, FNBP1L, HBEGF, IRS1, KALRN, NCAM1, NRG1, NTRK1, PREX2, PDGFB, RGL3, RGS22, SFRP1, SPTBN2, S1PR1* | 1.74 | 2.23E-02 |
| face morphogenesis/ 9 | *CLDN5, COL1A1, CRISPLD2, DKK1, DLX5, LEF1, MMP2, SCX, TGFB2* | 5.56 | 2.58E-02 |
| positive regulation of angiogenesis/ 18 | *CX3CL1, GATA6, TEK, WNT5A, ACVRL1, ADM2, F3, C3, FGF1, GREM1, HGF, HYAL1, NTRK1, PGF, THBS1, TWIST1, VEGFD, VASH2* | 2.90 | 2.47E-02 |
| male gonad development/ 16 | *GATA6, NKX3-1, WT1, WNT2B, WNT5A, AKR1C3, ESR1, FSTL3, INHBA, MAMLD1, NR0B1, SFRP1, STAR, TLR3, TFAP2C, TGFB2* | 3.15 | 2.41E-02 |
| negative regulation of cell proliferation/ 40 | *ADAMTS8, DLC1, KLF4, NOX4, NKX3-1, NRK, RERG, SOX4, TNFRSF8, WT1, ACVRL1, ADARB1, CDH13, CDK6, CDKN2B, FGF10, FGFRL1, INHBA, IGFBP6, ITGA1, IFITM1, MSX2, NTRK1, PTH1R, PTHLH, P3H2, PTGES, PTGS2, PTPRU, RARB, RARRES1, RARRES3, SCIN, SFRP1, SFRP4, TNS2, TES, TGFB2, VDR* | 1.87 | 3.33E-02 |
| negative regulation of cell growth/ 18 | *RERG, SMAD3, WFDC1, WT1, WNT11, ACVRL1, BDKRB1, GREM1, HYAL1, INHBA, NPR1, NOV, OSGIN2, PPARG, SFRP1, SLIT2, TGFB2, TNK1* | 2.76 | 4.02E-02 |
| positive regulation of MAP kinase activity/ 12 | *CD24, CD40, DIRAS1, NOX4, NEK10, RASGRP1, ADRA2A, EDN1, FGF1, PDGFC, PDGFB, TPD52L1* | 3.77 | 3.99E-02 |

**Supplementary table 4**

**Biological functional terms enriched in the upregulated and downregulated genes using Gene Ontology and KEGG analysis.**

**Terms enriched in the upregulated genes using Gene ontology analysis.**

| GO-Term/Gene Count | Gene | Fold enriched | Benjamini (p<0.05) |
| --- | --- | --- | --- |
| inflammatory response/ 27 | *CCL8, CCRL2, CXCL2, CXCL3, CD14, CD40, TNFAIP3, TNFRSF21, XCR1, AOX1, APOL3, BDKRB1, BDKRB2, CSF1, C3, EPHX2, GGT5, HRH1, HDAC9 HYAL1, IL15, LXN, LY75, NOD1, PTX3, PTGS2, TLR3* | 2.61 | 4.10E-02 |
| positive regulation of cell proliferation/ 30 | *NKX3-1, ST8SIA1, ADRA1D, ADRA2A, AKR1C3, CDC25B, CLDN7, CSF1, CSF3, DPP4, EFNB2, FGF20, FGF7, FGFR3, HGF, ID4, IL15, IL6R, IL6ST, LIFR, NRG1, NAMPT, OSR2, PTH1R, PGF, RAC2, RARB, SFRP1, SLC25A27, TNFSF13B* | 2.36 | 4.12E-02 |
| response to interferon-gamma/ 7 | *CXCL16, GCH1, IFITM1, IFITM2, IFITM3, KYNU, SNCA* | 10.69 | 2.97E-02 |
| signal transduction/ 56 | *CCL8, CD38, CD83, FYB, GNG11, NDP, RASGRP2, ARHGAP15, ARHGAP20, ARHGAP44, ARHGAP6, S100A6, STAC, SP110, STARD8, TNFRSF21, ASIC1 ACVRL1, ADRA2A, ANK1, APOL3, ARRDC2, CRABP2, CLIC2, CSF3R, C3, EPAS1, FGF13, FGF20, FGF7, GRP,, GRB7, IGFBP1, IGFBP2, IGFBP6, IL15RA, IL15, LY75, MAPK10, NAMPT, NOD1, PTCH2, PPARG, PDE3B, PGF, PTGES, RAC2, ROR2, RARB, SAV1, SECTM1, SIGIRR, SORL1, SDC4, TLR3, TNFSF13B* | 1.77 | 2.56E-02 |
| ureteric bud development/ 8 | *BMPER, SMAD3, WT1, OSR1, RARB, SFRP1, SLIT2, SDC4* | 7.72 | 3.28E-02 |
| immune response/ 27 | *OAS1, CCL8, CXCL2, CXCL3, CX3CL1, CD36, CD40, FYB, SMAD3, TNFRSF21, CSF3, C1R, C3, ENPP2, IFI6, IFITM2, IFITM3, IL1R1, IL15, LY75, HLA-DPA1 MBP, NFIL3, SECTM1, TGFBR3, TINAGL1, TNFSF13B* | 2.35 | 3.98E-02 |
| extracellular matrix organization/ 17 | *ABI3BP, ADAMTSL4, EGFLAM, CSGALNACT1, CRISPLD2, FBLN1, FOXF2, HPSE, HAPLN1, ITGB4, ITGB8, ICAM4, LAMA4, NDNF, VCAM1, VIT, VWA1* | 3.18 | 3.46E-02 |
| negative regulation of smooth muscle cell proliferation/ 7 | *KLF4, PPARGC1A, TNFAIP3, APOD, IL15, NPR1, PPARG* | 8.85 | 3.50E-02 |
| angiogenesis/18 | *TNFAIP2, ACVRL1, APOD, CEACAM1, EMCN, EPAS1, ENPEP, HAND2, HS6ST1 HIF3A, JAG1, NOV, NDNF, PDE3B, PGF, PLXND1, PTGS2, TMEM100* | 2.96 | 3.80E-02 |
| cellular response to tumor necrosis factor/ 12 | *CCL8, CX3CL1 NKX3-1, PPARGC1A, TNFRSF21, ZFP36, APOB, HYAL1, IL18BP, MAP3K5, SFRP1, VCAM1* | 4.00 | 5.14E-02 |
| positive regulation of osteoblast differentiation/ 9 | *CEBPD, HGF, ID4, IFITM1, IL6R, IL6ST, JAG1, MSX2, TP63* | 5.50 | 4.82E-02 |
| male gonad development/ 11 | *GATA6, NKX3-1, WT1, WNT2B, AKR1C3, MAMLD1, NR0B1, SFRP1, STAR, TLR3, TFAP2C* | 4.29 | 5.00E-02 |

**Terms enriched in the upregulated genes using KEGG-pathway analysis.**

| KEGG-PATHWAY/ Gene count | Gene | Fold enriched | Benjamini (p<0.05) |
| --- | --- | --- | --- |
| Pathways in cancer/ 29 | *GNG11, NFKBIA, NKX3-1, RASGRP2, SMAD3, WNT2B, ADCY4, BIRC7, BDKRB1, BDKRB2, CSF3R, DAPK2, EPAS1, FGF13, FGF20, FGF7, FGFR3, HGF, LAMA4, LPAR3, MAPK10, NTRK1, PTCH2, PPARG, PGF, PTGS2, RAC2, RARB, STAT5A* | 2.26 | 1.53E-02 |
| Complement and coagulation cascades/11 | *BDKRB1, BDKRB2, C1R, C2, C3, CFB, CFD, CFI, MASP1, PLAT, TFPI* | 4.87 | 7.92E-03 |
| TNF signaling pathway/ 13 | *CXCL2, CXCL3, CX3CL1, JUNB, NFKBIA, TNFAIP3, CSF1, IL15, MAPK10, MAP3K5, PTGS2, SOCS3, VCAM1* | 3.75 | 1.19E-02 |
| Pathways in cancer/ 29 | *GNG11, NFKBIA, NKX3-1, RASGRP2, SMAD3, WNT2B, ADCY4, BIRC7, BDKRB1, BDKRB2, CSF3R, DAPK2, EPAS1, FGF13, FGF20, FGF7, FGFR3, HGF, LAMA4, LPAR3, MAPK10, NTRK1, PTCH2, PPARG, PGF, PTGS2, RAC2, RARB, STAT5A* | 2.26 | 1.53E-02 |
| Complement and coagulation cascades/11 | *BDKRB1, BDKRB2, C1R, C2, C3, CFB, CFD, CFI, MASP1, PLAT, TFPI* | 4.87 | 7.92E-03 |

**Terms enriched in the downregulated genes using Gene ontology analysis.**

| GO-Term/Gene Count | Gene | Fold enriched | Benjamini (p<0.05) |
| --- | --- | --- | --- |
| cell adhesion/ 38 | *ADAM12, CD24, CASS4, NUAK1, PPFIBP1 SPOCK1, ARVCF, CDH2, CDH3, CDH6, CASK, COMP, COL1A1, COL8A1, COL12A1, CTGF, DSC3, FN1, ITGA4, ITGB3, ITGB5, LAMA1, LOXL2, NCAM1, NEDD9, NRP2, OLR1, PCDHA4, PCDHB12, PCDHB15, PCDHB2, SORBS1, SORBS2, S1PR1, SPON2, TNC THBS1 VCAN* | 3.10 | 4.78E-06 |
| collagen catabolic process/ 14 | *ADAMTS14, COL1A1, COL4A1, COL4A2, COL8A1, COL10A1, COL11A1, COL12A1, MMP10, MMP11, MMP15, MMP2, MMP3 MMP7* | 8.20 | 1.21E-05 |
| endodermal cell differentiation/ 10 | *COL4A2, COL8A1, COL11A1 COL12A1, FN1, INHBA, ITGA4, ITGB5, MMP15, MMP2* | 13.88 | 1.42E-05 |
| homophilic cell adhesion via plasma membrane adhesion molecules/ 20 | *FAT3, FAT4, AMIGO2, CDH2, CDH3, CDH6, CELSR2 CLSTN2, CADM1, DCHS1, DSC3, PCDHA4, PCDHB10, PCDHB12, PCDHB15, PCDHB2, PCDHB5, PCDHB7, PCDHGB1, TENM3* | 4.74 | 2.41E-05 |
| extracellular matrix organization/ 22 | *COMP, COL1A1, COL4A1, COL4A2, COL8A1, COL10A1, COL11A1, ELN, FN1, FBLN5, ITGA1, ITGA4, ITGB3, ITGB5 LAMA1, LOX, MFAP5 PXDN, PDGFB, TNC, THBS1, VCAN* | 4.21 | 2.88E-05 |
| collagen fibril organization/ 10 | *ADAMTS14, COL1A1, COL11A1, COL12A1, GREM1, LOXL2, LOX, MMP11, SCX, TGFB2* | 9.61 | 2.41E-04 |
| positive regulation of cell migration/ 19 | *CD274, F2RL1, GLI1, WNT11, F3, COL1A1, EDN1, FGF1, HBEGF IGF1, LEF1, NTF3, PDGFC, PDGFB, SEMA3D, SEMA3F, SEMA7A S1PR1, THBS1* | 3.87 | 6.83E-04 |
| skeletal system development/ 16 | *NKX3-2, SOX4, ALPL, COMP, COL1A1, COL10A1, COL12A1, DLX5, EXTL1, FGFRL1, IGF1, IGF2, PTHLH, TGFB2 VCAN VDR* | 4.38 | 1.05E-03 |
| wound healing/ 12 | *VANGL2, WNT5A, CDH3, DSP, FGF10, FN1, ITGB3, LOX, SCARB1, TNC, TGFB2, TPM1* | 5.62 | 2.10E-03 |
| positive regulation of MAP kinase activity/ 10 | *CD24,DIRAS1, NOX4, NEK10, RASGRP1, EDN1, FGF1, PDGFC, PDGFB, TPD52L1* | 6.35 | 5.34E-03 |
| positive regulation of endothelial cell migration/ 9 | *TEK, WNT5A ANGPT1, EDN1, FGF1, ITGB3, NRP2, SCARB1, THBS1* | 7.33 | 5.16E-03 |
| cell morphogenesis/10 | *FRY, NOX4, GREM1 IL7R PDPN SHROOM2, SHROOM3, TENM3, TGFB2, VDR* | 6.05 | 6.68E-03 |
| positive regulation of fibroblast proliferation/ 9 | *WNT2, WNT5A, CDK6, ESR1, FGF10, FN1, IGF1, PDGFC, PDGFB* | 6.25 | 1.44E-02 |
| extracellular matrix disassembly/ 10 | *HTRA1, CAPNS2, ELN, FN1, MMP10, MMP11, MMP15, MMP2, MMP3, MMP7* | 4.93 | 2.87E-02 |
| positive regulation of gene expression/ 19 | *WNT11, ACTC1, ADM2, ANK3, CDH3, CTGF, CDK6, FN1, FOXD1, INHBA, IL7R LEF1, MAPK8, PDGFB, SCX, SFRP4, TNC, TGFB2, VDR* | 2.72 | 3.52E-02 |
| angiogenesis/ 17 | *ARHGAP22, TEK, ADM2, ANGPT1, COL4A2, COL8A1, CTGF, FGF1, FGF10, FN1, MMP2, NRXN3, NRP2, S1PR1, SRPX2, TGFB2, VEGFD* | 2.86 | 4.45E-02 |
| positive regulation of cell proliferation/ 27 | *BAMBI, GLI1, SOX4 WNT2, CTGF, CRLF1, EDN1, EDNRB, FGF1, FN1, GREM1, HBEGF, IGF1, IGF2, IRS1, IL31RA, LEF1, NTF3, PTHLH, PDGFC, PDGFB, SCX, TNC, THBS1, TGFB2, TNFSF4, VEGFD* | 2.17 | 4.19E-02 |
| axon guidance/ 14 | *WNT5A, ANK3, DLX5, ENAH, FOXD1, KIF26B, KIF5C, NCAM1, NRXN3, NRP2, SEMA3F, SPTBN2, SPON2, TGFB2* | 3.30 | 4.10E-02 |

**Terms enriched in the downregulated genes using KEGG-pathway analysis.**

| KEGG-PATHWAY/ Gene count | Gene | Fold enriched | Benjamini (p<0.05) |
| --- | --- | --- | --- |
| Dilated cardiomyopathy/ 14 | *ACTC1, ADCY2, CACNA2D3, DES, IGF1, ITGA1, ITGA4, ITGB3, ITGB5, PLN, SGCD, TGFB2, TPM1, TNNT2* | 6.03 | 7.75E-05 |
| receptor interaction/ 14 | *COMP, COL1A1, COL4A1, COL4A2, COL11A1, FN1, ITGA1, ITGA4, ITGB3, ITGB5, LAMA1, TNC, TNN, THBS1* | 5.82 | 5.89E-05 |
| Focal adhesion/ 20 | *MET, COMP, COL1A1, COL4A1, COL4A2, COL11A1 FN1, IGF1, ITGA1, ITGA4) , ITGB3, ITGB5, LAMA1, MAPK8, PDGFC PDGFB, TNC, TNN, THBS1, VEGFD* | 3.51 | 2.17E-04 |
| Wnt signaling pathway/ 16 | *BAMBI, VANGL2, WNT11, WNT2 WNT5A, CTNNBIP1, DKK2, DAAM1, FZD7, GPC4, LEF1, MMP7, MAPK8, NKD2, PRICKLE1 SFRP4* | 4.19 | 2.52E-04 |
| PI3K-Akt signaling pathway/ 26 | *MET, TEK, ANGPT1, COMP, COL1A1, COL4A1, COL4A2, COL11A1, CDK6, FGF1, FGF10, FN1, IGF1, IRS1, ITGA1, ITGA4, ITGB3, ITGB5, IL7R, LAMA1, PDGFC, PDGFB, TNC, TNN, THBS1, VEGFD* | 2.73 | 2.92E-04 |
| Hypertrophic cardiomyopathy (HCM)/ 12 | *ACTC1, CACNA2D3, DES, IGF1, ITGA1, ITGA4, ITGB3, ITGB5, SGCD, TGFB2, TPM1, TNNT2* | 5.57 | 2.66E-04 |
| Pathways in cancer/ 27 | *CXCR4, GLI1, MECOM, MET, RASGRP1, RASGRP3, WNT11, WNT2, WNT5A, ADCY2, COL4A1, COL4A2, CDK6, CDKN2B, EDNRB, FGF1, FGF10, FN1, FZD7, IGF1, LAMA1, LEF1, MMP2, MAPK8, PDGFB, TGFB2, VEGFD* | 2.49 | 6.83E-04 |
| Proteoglycans in cancer/ 17 | *MET, WNT11, WNT2, WNT5A, ANK3, ESR1, FN1, FZD7, HBEGF, IGF1, IGF2, ITGB3, ITGB5, MMP2, MRAS, THBS1* | 3.08 | 2.85E-03 |
| Arrhythmogenic right ventricular cardiomyopathy (ARVC)/ 10 | *CDH2, CACNA2D3, DES, DSP, ITGA1, ITGA4, ITGB3, ITGB5, LEF1, SGCD* | 5.10 | 2.83E-03 |
| TGF-beta signaling pathway/ 9 | *BAMBI, SMURF2, TGIF2, ACVR1C, CDKN2B, GDF6, INHBA, THBS1, TGFB2* | 3.88 | 4.06E-02 |
| Protein digestion and absorption/ 9 | *COL1A1, COL4A1, COL4A2, COL10A1, COL11A1, COL12A1, COL21A1, ELN, SLC8A1* | 3.70 | 4.94E-02 |

**Supplementary table 5**

**Exosomal gene expression in control and A83-01-treated eMSCs are listed in the top exosomal markers in the Exocarta database**

| EnsemblGeneID | Exosome Genes | Fold change log_2_ | | FDR | Ave Expr |
| --- | --- | --- | --- | --- | --- |
|  |  | **Control** | **A83-01** |  |  |
| ENSG00000104537 | *ANXA13* | 0 | 2.783 | 1.81E-02 | -5.52 |
| ENSG00000143412 | *ANXA9* | 0 | 1.738 | 1.02E-02 | -1.47 |
| ENSG00000109511 | *ANXA10* | 0 | 1.579 | 2.26E-01 | -5.31 |
| ENSG00000140678 | *ITGAM/CD11c* | 0 | 1.341 | 2.98E-01 | -3.54 |
| ENSG00000140678 | *ITGAX/CD11b* | 0 | 1.341 | 2.98E-01 | -3.54 |
| ENSG00000105514 | *RAB3D* | 0 | 1.213 | 6.93E-03 | 3.984 |
| ENSG00000148175 | *STOM* | 0 | 1.18 | 1.15E-03 | 8.19 |
| ENSG00000172270 | *BSG* | 0 | 0.827 | 4.00E-03 | 10.10 |
| ENSG00000122359 | *ANXA11* | 0 | 0.803 | 1.69E-04 | 8.45 |
| ENSG00000110651 | *CD81* | 0 | 0.783 | 9.68E-04 | 10.07 |
| ENSG00000204632 | *HLA-G* | 0 | 0.748 | 1.20E-02 | 1.38 |
| ENSG00000091409 | *ITGA6* | 0 | 0.672 | 5.66E-02 | 7.38 |
| ENSG00000163631 | *ALB* | 0 | 0.586 | 5.16E-01 | -4.30 |
| ENSG00000137575 | *SDCBP* | 0 | 0.568 | 6.39E-03 | 8.24 |
| ENSG00000137575 | *SDCBP* | 0 | 0.568 | 6.39E-03 | 8.24 |
| ENSG00000143545 | *RAB13* | 0 | 0.564 | 5.49E-02 | 7.045 |
| ENSG00000076944 | *STXBP2* | 0 | 0.542 | 7.55E-02 | 1.216 |
| ENSG00000204389 | *HSPA1A* | 0 | 0.522 | 3.72E-03 | 5.07 |
| ENSG00000137312 | *FLOT1* | 0 | 0.52 | 1.88E-03 | 7.67 |
| ENSG00000137312 | *FLOT1* | 0 | 0.520 | 1.88E-03 | 7.67 |
| ENSG00000135046 | *ANXA1* | 0 | 0.452 | 5.88E-02 | 9.11 |
| ENSG00000149925 | *ALDOA* | 0 | 0.410 | 1.49E-02 | 9.75 |
| ENSG00000175899 | *A2M* | 0 | 0.386 | 1.88E-01 | 8.16 |
| ENSG00000134333 | *LDHA* | 0 | 0.381 | 1.45E-01 | 8.97 |
| ENSG00000135404 | *CD63* | 0 | 0.375 | 4.13E-02 | 10.59 |
| ENSG00000111640 | *GAPDH* | 0 | 0.336 | 6.74E-02 | 10.72 |
| ENSG00000163931 | *TKT* | 0 | 0.332 | 5.92E-02 | 8.72 |
| ENSG00000010278 | *CD9* | 0 | 0.320 | 9.09E-02 | 7.73 |
| ENSG00000108679 | *LGALS3BP* | 0 | 0.283 | 4.21E-02 | 9.84 |
| ENSG00000160948 | *VPS28* | 0 | 0.261 | 3.57E-02 | 6.531 |
| ENSG00000148180 | *GSN* | 0 | 0.245 | 1.27E-01 | 6.09 |
| ENSG00000156508 | *EEF1A1* | 0 | 0.241 | 1.41E-01 | 8.41 |
| ENSG00000132589 | *FLOT2* | 0 | 0.211 | 5.37E-02 | 6.14 |
| ENSG00000049245 | *VAMP3* | 0 | 0.201 | 1.11E-01 | 7.076 |
| ENSG00000116266 | *STXBP3* | 0 | 0.186 | 1.81E-01 | 5.827 |
| ENSG00000075785 | *RAB7A* | 0 | 0.172 | 1.93E-01 | 8.37 |
| ENSG00000164924 | *YWHAZ* | 0 | 0.167 | 2.11E-01 | 8.16 |
| ENSG00000170248 | *PDCD6IP/ALIX* | 0 | 0.167 | 1.61E-01 | 7.53 |
| ENSG00000138279 | *ANXA7* | 0 | 0.158 | 1.94E-01 | 7.00 |
| ENSG00000213719 | *CLIC1* | 0 | 0.156 | 3.17E-01 | 8.71 |
| ENSG00000089685 | *BIRC5* | 0 | 0.152 | 5.95E-01 | 5.33 |
| ENSG00000111669 | *TPI1* | 0 | 0.150 | 1.51E-01 | 7.90 |
| ENSG00000067225 | *PKM* | 0 | 0.139 | 3.55E-01 | 10.89 |
| ENSG00000172354 | *GNB2* | 0 | 0.127 | 2.42E-01 | 7.54 |
| ENSG00000103496 | *STX4* | 0 | 0.121 | 2.84E-01 | 5.629 |
| ENSG00000127314 | *RAP1B* | 0 | 0.118 | 3.01E-01 | 4.56 |
| ENSG00000079805 | *DNM2* | 0 | 0.107 | 3.37E-01 | 7.019 |
| ENSG00000005893 | *LAMP2* | 0 | 0.103 | 4.27E-01 | 7.98 |
| ENSG00000196975 | *ANXA4* | 0 | 0.101 | 3.56E-01 | 6.63 |
| ENSG00000165527 | *ARF6* | 0 | 0.094 | 5.77E-01 | 6.892 |
| ENSG00000122958 | *VPS26A* | 0 | 0.086 | 4.48E-01 | 6.040 |
| ENSG00000164111 | *ANXA5* | 0 | 0.078 | 6.58E-01 | 10.25 |
| ENSG00000134247 | *PTGFRN* | 0 | 0.077 | 7.51E-01 | 8.21 |
| ENSG00000087460 | *GNAS* | 0 | 0.077 | 6.57E-01 | 10.31 |
| ENSG00000087460 | *GNAS* | 0 | 0.077 | 6.57E-01 | 10.31 |
| ENSG00000196262 | *PPIA* | 0 | 0.076 | 6.26E-01 | 6.24 |
| ENSG00000108219 | *TSPAN14* | 0 | 0.075 | 6.76E-01 | 7.527 |
| ENSG00000167658 | *EEF2* | 0 | 0.075 | 5.58E-01 | 11.02 |
| ENSG00000117450 | *PRDX1* | 0 | 0.073 | 6.95E-01 | 8.89 |
| ENSG00000100380 | *ST13* | 0 | 0.072 | 5.74E-01 | 6.20 |
| ENSG00000100568 | *VTI1B* | 0 | 0.049 | 6.75E-01 | 5.91 |
| ENSG00000167461 | *RAB8A* | 0 | 0.044 | 7.18E-01 | 6.05 |
| ENSG00000136238 | *RAC1* | 0 | 0.041 | 7.31E-01 | 7.95 |
| ENSG00000108953 | *YWHAE* | 0 | 0.041 | 7.11E-01 | 8.40 |
| ENSG00000163399 | *ATP1A1* | 0 | 0.041 | 7.96E-01 | 8.82 |
| ENSG00000002549 | *LAP3* | 0 | 0.027 | 9.09E-01 | 5.437 |
| ENSG00000129925 | *TMEM8A* | 0 | 0.020 | 8.93E-01 | 6.370 |
| ENSG00000167553 | *TUBA1C* | 0 | 0.013 | 9.68E-01 | 7.20 |
| ENSG00000119541 | *VPS4B* | 0 | 0.011 | 9.51E-01 | 5.939 |
| ENSG00000136830 | *FAM129B* | 0 | 0.007 | 9.73E-01 | 8.501 |
| ENSG00000137807 | *KIF23* | 0 | -0.001 | 9.98E-01 | 5.698 |
| ENSG00000182149 | *IST1* | 0 | -0.016 | 8.94E-01 | 6.971 |
| ENSG00000174903 | *RAB1B* | 0 | -0.021 | 8.47E-01 | 6.970 |
| ENSG00000111716 | *LDHB* | 0 | -0.022 | 8.80E-01 | 8.99 |
| ENSG00000111716 | *LDHB* | 0 | -0.022 | 8.80E-01 | 8.99 |
| ENSG00000128245 | *YWHAH* | 0 | -0.030 | 9.03E-01 | 7.12 |
| ENSG00000070831 | *CDC42* | 0 | -0.033 | 8.20E-01 | 7.12 |
| ENSG00000109113 | *RAB34* | 0 | -0.034 | 8.02E-01 | 7.227 |
| ENSG00000111540 | *RAB5B* | 0 | -0.037 | 7.31E-01 | 7.70 |
| ENSG00000080371 | *RAB21* | 0 | -0.042 | 7.76E-01 | 5.546 |
| ENSG00000143761 | *ARF1* | 0 | -0.049 | 6.88E-01 | 8.91 |
| ENSG00000108828 | *VAT1* | 0 | -0.051 | 7.76E-01 | 8.733 |
| ENSG00000009844 | *VTA1* | 0 | -0.056 | 6.67E-01 | 6.039 |
| ENSG00000102144 | *PGK1* | 0 | -0.061 | 6.55E-01 | 8.09 |
| ENSG00000124209 | *RAB22A* | 0 | -0.069 | 5.91E-01 | 6.021 |
| ENSG00000108774 | *RAB5C* | 0 | -0.071 | 5.07E-01 | 5.90 |
| ENSG00000111737 | *RAB35* | 0 | -0.080 | 5.05E-01 | 5.750 |
| ENSG00000108424 | *KPNB1* | 0 | -0.083 | 5.86E-01 | 8.24 |
| ENSG00000168003 | *SLC3A2* | 0 | -0.086 | 8.28E-01 | 7.37 |
| ENSG00000166913 | *YWHAB* | 0 | -0.093 | 4.24E-01 | 8.21 |
| ENSG00000166913 | *YWHAB* | 0 | -0.093 | 4.24E-01 | 8.21 |
| ENSG00000105974 | *CAV1* | 0 | -0.097 | 7.95E-01 | 9.89 |
| ENSG00000182718 | *ANXA2* | 0 | -0.106 | 6.81E-01 | 9.42 |
| ENSG00000084733 | *RAB10* | 0 | -0.113 | 3.71E-01 | 6.928 |
| ENSG00000026508 | *CD44* | 0 | -0.114 | 4.71E-01 | 10.573 |
| ENSG00000074319 | *TSG101* | 0 | -0.125 | 3.28E-01 | 6.01 |
| ENSG00000074800 | *ENO1* | 0 | -0.135 | 4.08E-01 | 9.63 |
| ENSG00000114353 | *GNAI2* | 0 | -0.136 | 2.78E-01 | 8.74 |
| ENSG00000092531 | *SNAP23* | 0 | -0.138 | 4.23E-01 | 5.807 |
| ENSG00000080824 | *HSP90AA1* | 0 | -0.141 | 2.64E-01 | 9.15 |
| ENSG00000169710 | *FASN* | 0 | -0.153 | 4.66E-01 | 7.02 |
| ENSG00000069329 | *VPS35* | 0 | -0.156 | 1.52E-01 | 7.064 |
| ENSG00000150753 | *CCT5* | 0 | -0.156 | 3.44E-01 | 7.44 |
| ENSG00000129472 | *RAB2B* | 0 | -0.163 | 2.63E-01 | 4.723 |
| ENSG00000123416 | *TUBA1B* | 0 | -0.167 | 5.67E-01 | 8.72 |
| ENSG00000151532 | *VTI1A* | 0 | -0.168 | 1.81E-01 | 4.32 |
| ENSG00000144566 | *RAB5A* | 0 | -0.169 | 1.98E-01 | 6.44 |
| ENSG00000118193 | *KIF14* | 0 | -0.172 | 5.71E-01 | 3.973 |
| ENSG00000096384 | *HSP90AB1* | 0 | -0.184 | 1.46E-01 | 9.11 |
| ENSG00000067560 | *RHOA* | 0 | -0.189 | 2.43E-01 | 8.96 |
| ENSG00000134308 | *YWHAQ* | 0 | -0.190 | 2.33E-01 | 7.98 |
| ENSG00000103966 | *EHD4* | 0 | -0.197 | 5.31E-01 | 5.21 |
| ENSG00000131473 | *ACLY* | 0 | -0.200 | 2.04E-01 | 8.15 |
| ENSG00000073969 | *NSF* | 0 | -0.206 | 8.77E-02 | 5.845 |
| ENSG00000119396 | *RAB14* | 0 | -0.211 | 1.53E-01 | 6.79 |
| ENSG00000120438 | *TCP1* | 0 | -0.212 | 7.73E-02 | 7.03 |
| ENSG00000181789 | *COPG1* | 0 | -0.220 | 1.39E-01 | 8.159 |
| ENSG00000132341 | *RAN* | 0 | -0.224 | 1.29E-01 | 7.27 |
| ENSG00000108518 | *PFN1* | 0 | -0.228 | 2.17E-01 | 8.91 |
| ENSG00000104388 | *RAB2A* | 0 | -0.238 | 7.82E-02 | 7.185 |
| ENSG00000129083 | *COPB1* | 0 | -0.240 | 7.08E-02 | 8.105 |
| ENSG00000166598 | *HSP90B1* | 0 | -0.254 | 2.07E-01 | 10.29 |
| ENSG00000163468 | *CCT3* | 0 | -0.262 | 4.89E-02 | 7.87 |
| ENSG00000165280 | *VCP* | 0 | -0.266 | 2.71E-02 | 9.04 |
| ENSG00000197102 | *DYNC1H1* | 0 | -0.270 | 5.11E-02 | 9.096 |
| ENSG00000138069 | *RAB1A* | 0 | -0.272 | 2.77E-02 | 7.78 |
| ENSG00000130985 | *UBA1* | 0 | -0.276 | 3.18E-02 | 8.67 |
| ENSG00000057608 | *GDI2* | 0 | -0.290 | 2.30E-02 | 7.75 |
| ENSG00000057608 | *GDI2* | 0 | -0.290 | 2.30E-02 | 7.75 |
| ENSG00000101444 | *AHCY* | 0 | -0.303 | 1.37E-02 | 6.37 |
| ENSG00000075624 | *ACTB* | 0 | -0.307 | 3.71E-01 | 11.60 |
| ENSG00000122218 | *COPA* | 0 | -0.310 | 2.08E-02 | 8.479 |
| ENSG00000184009 | *ACTG1* | 0 | -0.315 | 3.21E-01 | 11.42 |
| ENSG00000184009 | *ACTG1* | 0 | -0.315 | 3.21E-01 | 11.42 |
| ENSG00000085117 | *CD82* | 0 | -0.327 | 6.51E-02 | 5.30 |
| ENSG00000138772 | *ANXA3* | 0 | -0.333 | 4.53E-01 | 2.69 |
| ENSG00000141367 | *CLTC* | 0 | -0.374 | 1.53E-02 | 9.45 |
| ENSG00000213177 | *PRDX2P4* | 0 | -0.397 | 7.99E-02 | 0.95 |
| ENSG00000175582 | *RAB6A* | 0 | -0.399 | 2.20E-02 | 6.76 |
| ENSG00000099246 | *RAB18* | 0 | -0.410 | 6.74E-03 | 6.825 |
| ENSG00000140545 | *MFGE8* | 0 | -0.440 | 4.59E-02 | 8.41 |
| ENSG00000172757 | *CFL1* | 0 | -0.442 | 5.40E-02 | 9.70 |
| ENSG00000172757 | *CFL1* | 0 | -0.442 | 5.40E-02 | 9.70 |
| ENSG00000044574 | *HSPA5* | 0 | -0.442 | 4.62E-02 | 9.51 |
| ENSG00000197043 | *ANXA6* | 0 | -0.445 | 1.85E-02 | 9.16 |
| ENSG00000155380 | *SLC16A1* | 0 | -0.458 | 6.74E-03 | 6.47 |
| ENSG00000153310 | *FAM49B* | 0 | -0.481 | 2.87E-02 | 4.223 |
| ENSG00000078369 | *GNB1* | 0 | -0.486 | 4.76E-03 | 8.62 |
| ENSG00000197702 | *PARVA* | 0 | -0.493 | 3.03E-02 | 7.844 |
| ENSG00000172115 | *CYCS* | 0 | -0.496 | 2.41E-02 | 5.53 |
| ENSG00000166226 | *CCT2* | 0 | -0.510 | 3.84E-03 | 7.43 |
| ENSG00000130402 | *ACTN4* | 0 | -0.514 | 4.92E-02 | 8.89 |
| ENSG00000130402 | *ACTN4* | 0 | -0.514 | 4.92E-02 | 8.89 |
| ENSG00000072274 | *TFRC* | 0 | -0.556 | 1.85E-01 | 5.73 |
| ENSG00000072274 | *TFRC* | 0 | -0.556 | 1.85E-01 | 5.73 |
| ENSG00000147065 | *MSN* | 0 | -0.600 | 6.68E-03 | 8.64 |
| ENSG00000170017 | *ALCAM* | 0 | -0.604 | 1.49E-02 | 7.706 |
| ENSG00000109971 | *HSPA8/HSC70* | 0 | -0.672 | 9.28E-03 | 9.23 |
| ENSG00000092820 | *EZR* | 0 | -0.684 | 2.12E-02 | 6.65 |
| ENSG00000170027 | *YWHAG* | 0 | -0.688 | 8.30E-04 | 7.83 |
| ENSG00000196924 | *FLNA* | 0 | -0.698 | 3.81E-02 | 10.94 |
| ENSG00000150093 | *ITGB1/CD29* | 0 | -0.917 | 1.33E-03 | 9.74 |
| ENSG00000150093 | *ITGB1* | 0 | -0.917 | 1.33E-03 | 9.74 |
| ENSG00000167552 | *TUBA1A* | 0 | -1.16 | 6.60E-04 | 8.21 |
| ENSG00000117525 | *F3(TF)/CD142* | 0 | -1.408 | 8.16E-04 | 6.74 |
| ENSG00000127324 | *TSPAN8* | 0 | -2.193 | 1.35E-01 | -4.82 |
| ENSG00000137801 | *THBS1* | 0 | -2.45 | 9.42E-04 | 10.16 |

**Supplementary table 6**

**MicroRNA expression in A83-01-treated and control eMSCs**

| Genes | Log_2_ Fold change | | FDR | Avg Expr |
| --- | --- | --- | --- | --- |
|  | **Control** | **A83-01** |  |  |
| *MIR4697HG* | 0 | 3.09 | 7.02E-03 | -5.23 |
| *MIR145* | 0 | 2.44 | 1.04E-02 | -4.18 |
| *MIR4500HG* | 0 | 2.19 | 5.92E-02 | -4.93 |
| *MIR3622A* | 0 | 2.06 | 6.54E-02 | -4.45 |
| *MIR155HG* | 0 | 2.05 | 7.52E-05 | 2.19 |
| *MIR6821* | 0 | 1.92 | 4.48E-02 | -5.02 |
| *MIR4257* | 0 | 1.67 | 2.52E-02 | -5.59 |
| *MIR199A1* | 0 | 1.65 | 1.54E-02 | -3.26 |
| *MIR6784* | 0 | 1.51 | 3.73E-02 | -4.29 |
| *MIR221* | 0 | 1.46 | 3.14E-02 | -1.09 |
| *MIR2116* | 0 | 1.34 | 1.70E-01 | -5.80 |
| *MIR450B* | 0 | 1.31 | 6.62E-02 | -4.13 |
| *MIR222HG* | 0 | 1.21 | 2.85E-03 | 3.61 |
| *MIR92B* | 0 | 1.19 | 2.79E-03 | 0.02 |
| *MIR4271* | 0 | 1.18 | 3.95E-01 | -5.97 |
| *MIR3142HG* | 0 | 1.05 | 4.29E-02 | -0.85 |
| *MIR6772* | 0 | 0.94 | 2.87E-01 | -4.14 |
| *MIR647* | 0 | 0.91 | 1.12E-01 | -2.54 |
| *MIR7111* | 0 | 0.88 | 2.41E-01 | -3.88 |
| *MIR503* | 0 | 0.75 | 3.58E-02 | -0.99 |
| *MIR106B* | 0 | 0.74 | 4.07E-01 | -4.35 |
| *MIR6753* | 0 | 0.69 | 3.71E-01 | -2.70 |
| *MIR296* | 0 | 0.62 | 6.52E-01 | -4.27 |
| *MIR23B* | 0 | 0.60 | 3.26E-01 | -4.85 |
| *MIR3189* | 0 | 0.59 | 3.85E-01 | -2.17 |
| *MIR503HG* | 0 | 0.58 | 4.05E-03 | 6.39 |
| *MIR4458HG* | 0 | 0.51 | 6.21E-02 | 2.32 |
| *MIR570* | 0 | 0.50 | 4.21E-01 | -2.21 |
| *MIR3911* | 0 | 0.49 | 1.24E-01 | -0.77 |
| *MIR4737* | 0 | 0.47 | 5.42E-01 | -3.75 |
| *MIR3681HG* | 0 | 0.47 | 3.79E-01 | -2.35 |
| *MIR22HG* | 0 | 0.47 | 2.71E-02 | 5.87 |
| *MIR646HG* | 0 | 0.44 | 2.40E-01 | -0.60 |
| *MIR210HG* | 0 | 0.43 | 1.58E-01 | 1.98 |
| *MIR6757* | 0 | 0.42 | 6.80E-01 | -4.53 |
| *MIR940* | 0 | 0.41 | 2.47E-01 | -1.10 |
| *MIR4730* | 0 | 0.40 | 8.28E-01 | -5.84 |
| *MIR6820* | 0 | 0.38 | 2.73E-01 | 1.89 |
| *MIR27B* | 0 | 0.37 | 5.10E-01 | -2.88 |
| *MIRLET7BHG* | 0 | 0.36 | 1.05E-01 | 3.55 |
| *MIR193BHG* | 0 | 0.33 | 3.53E-01 | -0.56 |
| *MIR641* | 0 | 0.29 | 7.23E-01 | -3.58 |
| *MIR93* | 0 | 0.28 | 7.94E-01 | -4.64 |
| *MIR616* | 0 | 0.24 | 6.29E-01 | -3.24 |
| *MIR103A2* | 0 | 0.21 | 7.33E-01 | -2.80 |
| *MIR1282* | 0 | 0.18 | 8.71E-01 | -2.95 |
| *MIR34A* | 0 | 0.16 | 5.42E-01 | 4.44 |
| *MIR29A* | 0 | 0.16 | 5.25E-01 | 2.80 |
| *MIR24-2* | 0 | 0.14 | 6.75E-01 | 3.60 |
| *MIR137HG* | 0 | 0.08 | 9.18E-01 | -2.68 |
| *MIR4701* | 0 | 0.07 | 9.35E-01 | -4.16 |
| *MIR25* | 0 | 0.05 | 9.21E-01 | -1.29 |
| *MIR2052HG* | 0 | 0.04 | 9.72E-01 | -4.83 |
| *MIR1254-1* | 0 | 0.03 | 9.69E-01 | -2.74 |
| *MIR23A* | 0 | 0.03 | 9.79E-01 | -4.94 |
| *MIR635* | 0 | 0.02 | 9.75E-01 | -2.11 |
| *MIR762* | 0 | -0.01 | 9.91E-01 | -4.06 |
| *MIR497HG* | 0 | -0.05 | 9.27E-01 | -0.88 |
| *MIR6087* | 0 | -0.05 | 9.77E-01 | -4.33 |
| *MIR100HG* | 0 | -0.09 | 5.44E-01 | 6.21 |
| *MIR4435-2HG* | 0 | -0.13 | 6.05E-01 | 5.20 |
| *MIR31HG* | 0 | -0.16 | 8.16E-01 | -2.64 |
| *MIR4453* | 0 | -0.20 | 4.02E-01 | 0.90 |
| *MIR6835* | 0 | -0.24 | 7.69E-01 | -3.34 |
| *MIRLET7D* | 0 | -0.24 | 7.63E-01 | -3.25 |
| *MIR4263* | 0 | -0.28 | 8.28E-01 | -4.05 |
| *MIR4653* | 0 | -0.30 | 7.56E-01 | -3.76 |
| *MIR4292* | 0 | -0.39 | 5.87E-01 | -2.34 |
| *MIR3682* | 0 | -0.43 | 5.58E-01 | -2.53 |
| *MIR621* | 0 | -0.48 | 3.03E-01 | -1.15 |
| *MIR186* | 0 | -0.56 | 2.42E-01 | -2.55 |
| *MIR378J* | 0 | -0.61 | 6.66E-01 | -4.25 |
| *MIR99AHG* | 0 | -0.75 | 4.05E-03 | 1.82 |
| *MIR600HG* | 0 | -0.81 | 4.50E-02 | -1.04 |
| *MIR6894* | 0 | -0.82 | 4.82E-01 | -3.74 |
| *MIR17HG* | 0 | -0.82 | 6.44E-02 | -1.72 |
| *MIR130B* | 0 | -1.05 | 1.06E-01 | -2.82 |
| *MIR133A1HG* | 0 | -1.65 | 3.10E-02 | -5.10 |
| *MIR199A2* | 0 | -1.88 | 5.73E-04 | -1.98 |
| *MIR181A2HG* | 0 | -1.93 | 4.55E-04 | 0.01 |
| *MIR7161* | 0 | -2.37 | 5.61E-02 | -5.38 |
